# Knowledge, Beliefs, and Practices Regarding Fibromyalgia Among Family Medicine Physicians and Residents in Saudi Arabia: A Cross-Sectional Study

**DOI:** 10.3390/healthcare14193201

**Published:** 2026-09-28

**Authors:** Abdulrahman Ali M. Khormi, Abdullah AlDhuwaihy, Fatma K. Al Dammas, Naif Jamal Aynusah, Abdullah Abolkhair, Abdulaziz B. Alanazi, Mohammed A. Alnoshan, Mohammed Abdullah Almurshid, Abdulrahman AlOsleb, Mohammed A. Omair

**Affiliations:** 1 Department of Internal Medicine, College of Medicine, Prince Sattam Bin Abdulaziz University, Al-Kharj 11942, Saudi Arabia; aa.khormi@psau.edu.sa; 2 College of Medicine, King Saud University, Riyadh 11472, Saudi Arabia; drabdulazizalanazi44@gmail.com (A.B.A.); malnoshan445@gmail.com (M.A.A.); mohammed.puchino@gmail.com (M.A.A.); dr.alosleb@gmail.com (A.A.); 3 Department of Anesthesia, College of Medicine, King Saud University Medical City, King Saud University, Riyadh 11472, Saudi Arabia; faldammas@ksu.edu.sa; 4 Department of Anesthesia, King Faisal Specialist Hospital and Research Center, Riyadh 11211, Saudi Arabia; nayef.ainousah@hotmail.com (N.J.A.); drabolkhair@gmail.com (A.A.); 5 Rheumatology Unit, Department of Medicine, King Saud University, Riyadh 11472, Saudi Arabia; momair@ksu.edu.sa

**Keywords:** fibromyalgia, health knowledge, attitudes, practice, physicians, family, primary health care, surveys and questionnaires, Saudi Arabia

## Abstract

**Background/Objectives:** Fibromyalgia (FM) is common but frequently underdiagnosed, and family medicine physicians are usually the first clinicians consulted by affected patients. This study assessed the knowledge, beliefs, and clinical practices regarding FM among family medicine physicians and residents in Saudi Arabia. **Methods:** An analytical cross-sectional online survey was conducted between July and December 2025 using convenience sampling and a questionnaire adapted from a previously published instrument. Knowledge (0–18) and belief (0–6) scores were reported descriptively, compared across groups with *t*-tests and analysis of variance, and explored with multivariable linear regression. **Results:** Of 113 participants (29.4% of the calculated target of 384), 60.2% were junior residents. The mean knowledge score was 9.7 ± 3.32 (53.6% of the maximum) and the mean belief score 4.1 ± 1.23 (67.5%). Only 39.8% knew the American College of Rheumatology criteria, 47.8% regarded FM as primarily psychological, and 39.8% felt confident differentiating FM from similar conditions; non-steroidal anti-inflammatory drugs were the most frequently selected drug class (62.8%). Belief scores were higher in academic centres (4.7 ± 1.19 vs. 3.9 ± 1.18; *p* = 0.002). The regression model was not significant overall (F (13, 99) = 1.476; *p* = 0.140), and its two nominally significant coefficients did not persist after accounting for collinearity. **Conclusions:** In this exploratory convenience sample, recognition of FM coexisted with limited command of diagnostic criteria, low diagnostic confidence, and guideline-discordant prescribing. The findings are not nationally representative but identify plausible targets for criteria-based training and continuing medical education.

## 1. Introduction

Fibromyalgia (FM) is a chronic disorder of central pain processing characterised by widespread musculoskeletal pain, fatigue, non-restorative sleep, and cognitive dysfunction [1,2,3]. It affects approximately 2–3% of the general population, predominantly women [4,5], and a pooled prevalence of 13.4% has been reported in Saudi Arabia [6]. Patients with FM incur healthcare costs about three times those of matched controls [7], yet FM remains among the most frequently underdiagnosed conditions in primary care [8].

The American College of Rheumatology (ACR) 1990 classification criteria required widespread pain and tenderness at 11 or more of 18 tender points [9]; the 2010 criteria replaced the tender-point examination with the Widespread Pain Index and Symptom Severity Scale [10], and the 2016 revision added a generalised pain criterion [11]. Diagnosis nevertheless remains difficult because there is no objective biomarker and symptoms overlap with those of other conditions [5], and patients consult a mean of 3.7 physicians before diagnosis [12]. European Alliance of Associations for Rheumatology’s (EULAR) recommendations prioritise patient education, exercise, and cognitive behavioural therapy, reserving amitriptyline, duloxetine, and pregabalin for persistent symptoms and advising against non-steroidal anti-inflammatory drugs (NSAIDs), corticosteroids, and strong opioids [13]; real-world practice, however, frequently diverges from these recommendations [14].

Physician knowledge gaps are well documented [15,16]. In Tabuk, Saudi Arabia, 42.3% of primary care physicians identified localised pain as a cardinal feature of FM and 86.5% considered NSAIDs, corticosteroids, or opioids appropriate [17]. Similar deficits have been reported among Egyptian family physicians [18] and specialty physicians [19], Saudi medical practitioners nationally [20], and doctors worldwide, of whom only 10% applied the ACR criteria [21]. Recent data on physicians remain scarce, and uncertainty persists about which specialty should lead FM care [22]. Existing Saudi studies are confined to single regions [17], mixed practitioner groups [15,20], students and physicians in Riyadh [23], or physical therapists [24].

This exploratory study therefore addressed three questions among family medicine physicians and residents in Saudi Arabia: (i) What level of FM knowledge and what beliefs about FM do they report? (ii) How closely do their reported practices align with the ACR criteria and EULAR recommendations? (iii) Which characteristics are associated with the knowledge score? No formal a priori hypotheses were specified.

## 2. Materials and Methods

### 2.1. Study Design, Setting, and Duration

An analytical cross-sectional study was conducted between July and December 2025 among family medicine physicians and residents working in the Ministry of Health, and military, academic, and primary healthcare facilities in Saudi Arabia. This study is reported in accordance with the Strengthening the Reporting of Observational Studies in Epidemiology (STROBE) statement [25] and, for the web-based survey, the Checklist for Reporting Results of Internet E-Surveys (CHERRIES) [26]; completed checklists are provided in Supplementary Materials Sections S1 and S2.

### 2.2. Participants

Eligible participants were family medicine consultants (board-certified), senior residents (postgraduate year three or beyond, R3+), and junior residents (postgraduate years one and two, R1–R2) of the Saudi Board of Family Medicine programme of any sex or nationality. The term “resident” is used in preference to “trainee” because all participants in the training categories were qualified physicians; interns, students, and allied health professionals were not eligible. Participants who declined electronic consent were excluded.

### 2.3. Recruitment and Survey Administration

The survey link was distributed through online professional networks and social media groups of family medicine physicians and residents centred in Riyadh. The survey was open, voluntary, and unincentivised, and region of practice was not recorded. Because the link could be forwarded, the number of physicians who viewed it is unknown; view, participation, and completion rates therefore could not be calculated, and duplicate submissions could not be excluded with certainty. Physicians who did not use these platforms could not be reached, a limitation of coverage rather than an eligibility criterion. Potential selection, geographic, and social desirability biases are discussed in Section 4.3.

### 2.4. Questionnaire and Scoring

Data were collected with a self-administered Google Forms (Google LLC, Mountain View, CA, USA) questionnaire adapted from the English-language instrument of Alatawi et al., which was developed for primary care physicians in Saudi Arabia [17]; the study aim was explained on the introductory page of the form. It was administered in English, the language of medical education and practice in Saudi Arabia; so, no translation was required. The knowledge, belief, and practice items were used without modification; only the sociodemographic response categories were adapted to Saudi family medicine settings. The questionnaire covered (i) sociodemographic and professional characteristics; (ii) knowledge of the ACR criteria, the 1990 tender-point requirement, clinical features, and treatment options; and (iii) beliefs and practices, including acceptance of FM as a distinct entity, beliefs about its nature, self-rated diagnostic confidence, perceived diagnostic status, sources of knowledge, and preferred managing service. The full item wording is shown in the Results (Section 3).

No psychometric validation data are published for the original instrument [17]. Before distribution, the questionnaire was reviewed by the research team, including a consultant rheumatologist and consultant anaesthetists with a chronic pain practice, for content and face validity; no psychometric revalidation or reliability analysis was undertaken. One point was awarded for each correct or positive response, giving maximum knowledge and belief scores of 18 and 6, respectively. Equal item weighting was used because no empirical weighting exists, consistent with the source instrument and comparable surveys [17,18,20]. As no validated cut-off values exist, scores are reported descriptively as mean ± standard deviation (SD) and as a percentage of the maximum, without categorical labels.

### 2.5. Study Size

Using the single-proportion formula (95% confidence level, 5% margin of error, assumed proportion 0.5), the target sample size was 384. The achieved sample of 113 (29.4% of the target) is addressed in Section 4.3.

### 2.6. Statistical Analysis

Analyses were performed with IBM SPSS Statistics version 23 (IBM Corp., Armonk, NY, USA). Categorical variables are presented as frequencies and percentages and continuous variables as mean ± SD. Scores were compared using independent-sample *t*-tests and one-way analysis of variance (ANOVA). The knowledge score was approximately symmetrical (skewness 0.36; kurtosis −0.34), with non-significant within-group Shapiro–Wilk tests in most groups; the belief score, a six-point count, departed from normality (Shapiro–Wilk *p* < 0.001).

A multivariable linear regression model was fitted with the knowledge score as the dependent variable and sex, age group, nationality, professional position, years of clinical experience, and hospital type as predictors (reference categories: male, <28 years, Saudi, consultant, <2 years, and non-academic centre). Standardised residuals were approximately normally distributed (mean 0.000; SD 0.940; range −2.50 to 2.55) and showed no pattern against predicted values (Durbin–Watson 2.02). Collinearity was assessed with tolerance and variance inflation factors (VIF), and because professional position and experience are structurally related, sensitivity models omitting each in turn were fitted. Full diagnostics are provided in Supplementary Materials Sections S3 and S4. A *p* value of ≤0.05 was considered statistically significant.

### 2.7. Ethical Considerations

This study was approved by the Institutional Review Board of King Saud University (approval number E-25-9895; 26 June 2025) and conducted in accordance with the Declaration of Helsinki [27]. Electronic informed consent was obtained from all participants; participation was voluntary and anonymous, participants could withdraw at any time without consequence, and no incentives were offered. Responses were stored in a protected electronic format accessible only to the research team.

## 3. Results

### 3.1. Participants

A total of 113 family medicine physicians and residents participated, corresponding to 29.4% of the calculated target. Most were male (57.5%), aged 28–35 years (38.1%), and Saudi (92.9%); 60.2% were junior residents, 12.4% senior residents, and 27.4% consultants, and 61.9% had less than 5 years of clinical experience (Table 1).

Overall, 24.8% of participants worked in academic and training centres and 75.2% in other centres, most commonly governmental or health cluster facilities (29.2%) and university hospitals (23.0%) (Figure 1).

### 3.2. Knowledge of Fibromyalgia

Only 39.8% of participants knew the ACR diagnostic criteria, and 66.7% of these used them in practice. Among those who did not use the criteria (*n* = 68), the most common reasons were that they were hard to master (27.9%), unreliable (19.1%), or complicated (16.2%). The 1990 tender-point requirement (11 of 18) was identified by 58.4%. Most participants (87.6%) favoured combined pharmacological and non-pharmacological management, and cognitive behavioural therapy (64.6%) and physiotherapy (54.9%) were the most frequently selected non-pharmacological options (Table 2).

Fatigue (85.8%) and widespread pain (84.1%) were the most frequently recognised features, whereas cognitive difficulties (43.4%) and morning stiffness (34.5%) were recognised less often, and 29.2% incorrectly selected localised pain (Figure 2).

NSAIDs were the most frequently selected drug class (62.8%), followed by tricyclic antidepressants (45.1%), muscle relaxants (43.4%), selective serotonin reuptake inhibitors (SSRIs; 38.9%), and duloxetine, a serotonin–noradrenaline reuptake inhibitor (SNRI; 38.1%). Gabapentin was selected by 18.6%, prednisolone by 7.1%, and opioids by 2.7% (Figure 3).

### 3.3. Beliefs and Practices

Most participants accepted FM as a distinct clinical entity (83.2%) and recognised its female predominance (89.4%), but 47.8% regarded it as primarily psychological. Confidence in recognising FM symptoms was reported by 49.6% and in differentiating FM from similar conditions by 39.8%. Most considered FM missed (50.4%) or underdiagnosed (40.7%), citing lack of knowledge (66.1%), similarity to other diseases (57.8%), and lack of training programmes (45.0%). Knowledge had been acquired mainly during postgraduate training (62.8%), and only 8.0% cited continuing medical education (CME). A multidisciplinary team was considered best placed to manage FM by 47.8%, rheumatologists by 22.1%, and family medicine physicians by 17.7% (Table 3).

### 3.4. Scores and Associated Factors

The mean knowledge score was 9.7 ± 3.32 of 18 (53.6% of the maximum) and the mean belief score 4.1 ± 1.23 of 6 (67.5%). Neither score differed significantly by sex, age group, nationality, professional position, or clinical experience (all *p* > 0.05). Belief scores were higher among physicians in academic and training centres than in other centres (4.7 ± 1.19 vs. 3.9 ± 1.18; t (111) = 3.228, *p* = 0.002), whereas knowledge scores did not differ (10.1 ± 3.28 vs. 9.5 ± 3.35; *p* = 0.437) (Table 4).

The multivariable regression model was not statistically significant (F (13, 99) = 1.476, *p* = 0.140; R^2^ = 0.162; adjusted R^2^ = 0.052). Two coefficients reached the 5% threshold: junior resident status (B = −4.24; 95% confidence interval [CI] −7.56 to −0.93; *p* = 0.013) and more than 15 years of experience (B = −6.87; 95% CI −12.81 to −0.92; *p* = 0.024). These were among the most collinear predictors (VIF 7.22 and 8.50; tolerance 0.139 and 0.118), and neither remained significant in sensitivity models omitting the collinear variable (junior resident status: B = −1.97, 95% CI −4.62 to 0.68, *p* = 0.143; more than 15 years: B = −2.87, 95% CI −8.34 to 2.61, *p* = 0.302). Neither reduced model was significant overall (*p* = 0.196 and *p* = 0.661), and no other variable was associated with the knowledge score (Table 5).

## 4. Discussion

In this exploratory survey of 113 family medicine physicians and residents, acceptance of FM as a legitimate clinical entity outpaced command of the criteria-based diagnostic and therapeutic knowledge needed to act on it. Because participation reached 29.4% of the target and recruitment centred on Riyadh, the findings are hypothesis-generating rather than representative.

Awareness of the ACR criteria (39.8%) was lower than in Tabuk (75.0%) [17] but consistent with other recent estimates: 31.1% among Saudi medical practitioners [20], 65.5% of Egyptian specialty physicians being unfamiliar with the criteria [19], and only 10% of doctors worldwide applying them [21]. One-third of our criteria-aware participants did not apply the criteria, citing complexity and difficulty of use, a pattern also reported in Egypt, where 52.8% of criteria-aware physicians did not apply them [19]. Awareness alone therefore does not ensure application, and usability at the point of care appears to be a barrier.

Fatigue and widespread pain were widely recognised, as among Egyptian family physicians [18], but cognitive symptoms and morning stiffness were not, and 29.2% selected localised pain, a misconception also reported in Tabuk (42.3%) [17]. Misattributing localised pain risks overdiagnosis in regional pain syndromes, whereas overlooking cognitive complaints neglects a feature that contributes to the Symptom Severity Scale [2,11]. Prescribing showed a similar gap: NSAIDs were the most frequently selected agents (62.8%) despite limited efficacy in centrally mediated pain [2,14], whereas agents with a stronger evidence base, such as duloxetine (38.1%) and gabapentin (18.6%), were selected less often [13]. Comparable preferences for NSAIDs and other non-recommended agents have been reported in Tabuk [17], Egypt [18], and internationally [21], suggesting that teaching may still reflect a peripheral, inflammatory model of pain.

Encouragingly, 87.6% endorsed multimodal management, as recommended by EULAR [13], comparing favourably with Tabuk (61.5%) [17] and approaching Egyptian physicians (94.2%) [19]. The gap therefore lies less in treatment philosophy than in pharmacological detail. Only 8.0% cited CME as their main source of FM knowledge; because CME is the principal route by which practising physicians update their knowledge as criteria evolve, this may partly explain the mismatch between recognition of FM and knowledge of its criteria.

Although 83.2% accepted FM as a distinct condition, 47.8% regarded it as primarily psychological, similar to the 51% reported in a recent meta-analysis [8] and the 49.6% reported among Egyptian physicians [19]; this belief may encourage stigmatisation and delay evidence-based care. Diagnostic confidence was lower than in Tabuk (59.6% and 55.8%) [17] but higher than among Egyptian specialty physicians [19]. Participants identified lack of knowledge and training as the leading barriers to diagnosis, consistent with earlier Saudi data showing limited formal education on FM [15] and with the lower criteria knowledge of general practitioners than of rheumatologists reported internationally [8].

Belief scores were higher in academic and training centres, possibly reflecting greater specialist collaboration, teaching, and exposure to current evidence. As a cross-sectional association, however, this cannot establish causality, and differences in career stage or case mix could account for it. Only 17.7% of participants considered family medicine physicians best placed to manage FM, echoing referral patterns in Saudi Arabia [15,17] and Egypt [18,19] and recent uncertainty about which specialty should lead FM care [22]. Because most patients first present in primary care, this diffusion of responsibility may itself delay diagnosis.

The regression analysis did not identify independent correlates of knowledge. The model was not significant overall, and its two nominally significant coefficients—junior resident status and more than 15 years of experience—were highly collinear, lost significance in sensitivity analyses, and were not significant in bivariate comparisons; they are therefore best regarded as artefacts of collinearity. Given the limited power and sparse strata, this is inconclusive rather than a null finding. Whether knowledge reflects recency of criteria-based training rather than seniority, as the successive revisions of the criteria [9,10,11], an earlier report of residents outperforming consultants [17], and more frequent criteria use by younger physicians [19] might suggest, requires an adequately powered study.

### 4.1. Implications for Practice and Education

These implications are suggested, not established, by an exploratory cross-sectional survey. Embedding the ACR criteria in clinical decision support may help criteria-aware physicians apply them; structured, criteria-based FM teaching could be integrated into undergraduate and postgraduate family medicine curricula; targeted CME may benefit physicians who have completed training; guidance on the limited role of NSAIDs and opioids should be disseminated more actively; and multidisciplinary pathways with clear referral criteria may clarify professional responsibility.

### 4.2. Implications for Research

Measuring genuine attitudes will require a validated Likert-type instrument rather than the binary items used here. Qualitative studies with family medicine physicians and residents could clarify why criteria-aware physicians do not apply the criteria, why many regard FM as psychological, and which training formats and content they value, thereby identifying priorities for educational programmes. Probability-based national sampling that records region of practice, and before-and-after evaluation of criteria-based training are also needed.

### 4.3. Strengths and Limitations

This is among the first studies to examine FM knowledge, beliefs, and practices specifically among Saudi family medicine physicians and residents, and it used an instrument previously applied in Saudi primary care, facilitating regional comparison.

Several limitations apply. First, the achieved sample was 29.4% of the target, reducing precision and power, particularly in sparse strata (11 participants with more than 15 years of experience and 8 non-Saudi respondents); null findings are therefore inconclusive. Second, because the survey was an open, forwardable link, no response rate could be calculated and non-response bias could not be assessed; its direction is uncertain, as physicians interested in chronic pain and those who felt poorly informed may have self-selected differently. Third, convenience sampling through Riyadh-centred online networks excluded physicians who did not use these platforms, and region of practice was not recorded; so, the findings are not nationally representative. Fourth, responses were self-reported and may reflect social desirability. Fifth, the questionnaire was not psychometrically validated in this population, equal item weighting may not reflect clinical importance, and the belief score departed from normality; so, its single significant group difference warrants confirmation with a rank-based test. Sixth, strong collinearity between professional position and experience (VIF 7.22 and 8.50) precluded estimation of their independent effects. Seventh, 60.2% of participants were junior residents; so, the pooled knowledge score (53.6% of the maximum) probably underestimates that of board-certified family physicians; the position-stratified estimates in Table 4 are more informative. Finally, the cross-sectional design precludes causal inference.

## 5. Conclusions

In this exploratory convenience sample of family medicine physicians and residents in Saudi Arabia, most participants recognised FM as a distinct condition, but familiarity with the diagnostic criteria was limited, diagnostic confidence was low, prescribing diverged from guideline recommendations, and the role of primary care was uncertain. No characteristic was independently associated with knowledge once collinearity was accounted for. Given the limited sample and recruitment concentrated in Riyadh, the findings cannot be generalised nationally, but they support structured, criteria-based training, targeted CME, and clearer referral pathways whose effectiveness should be evaluated in adequately powered, representative studies.

## Figures and Tables

**Figure 1:**
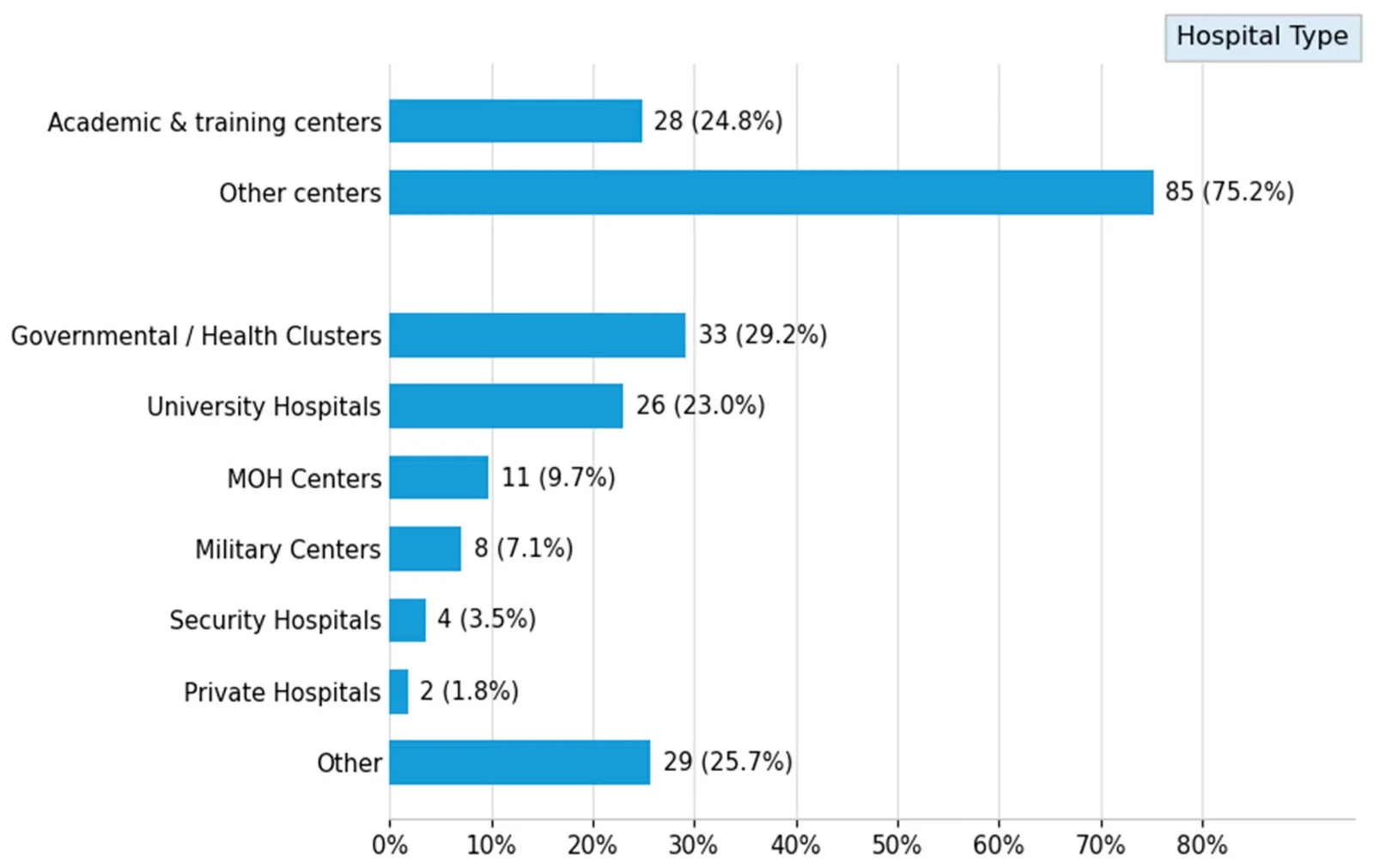
Distribution of participants by current hospital.

**Figure 2:**
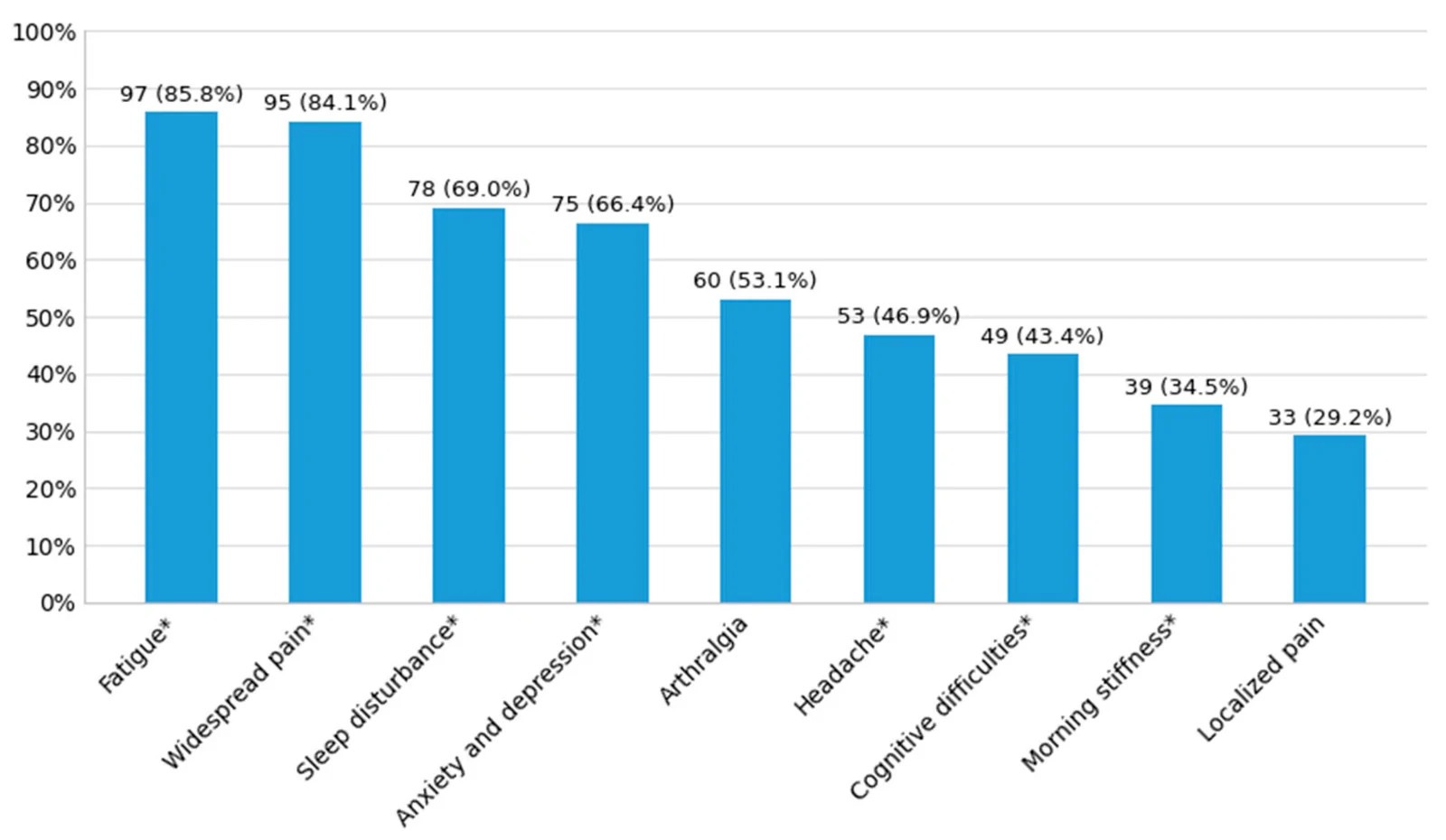
Knowledge about the symptoms of fibromyalgia. * Correct answer; items marked with an asterisk contributed to the total knowledge score.

**Figure 3:**
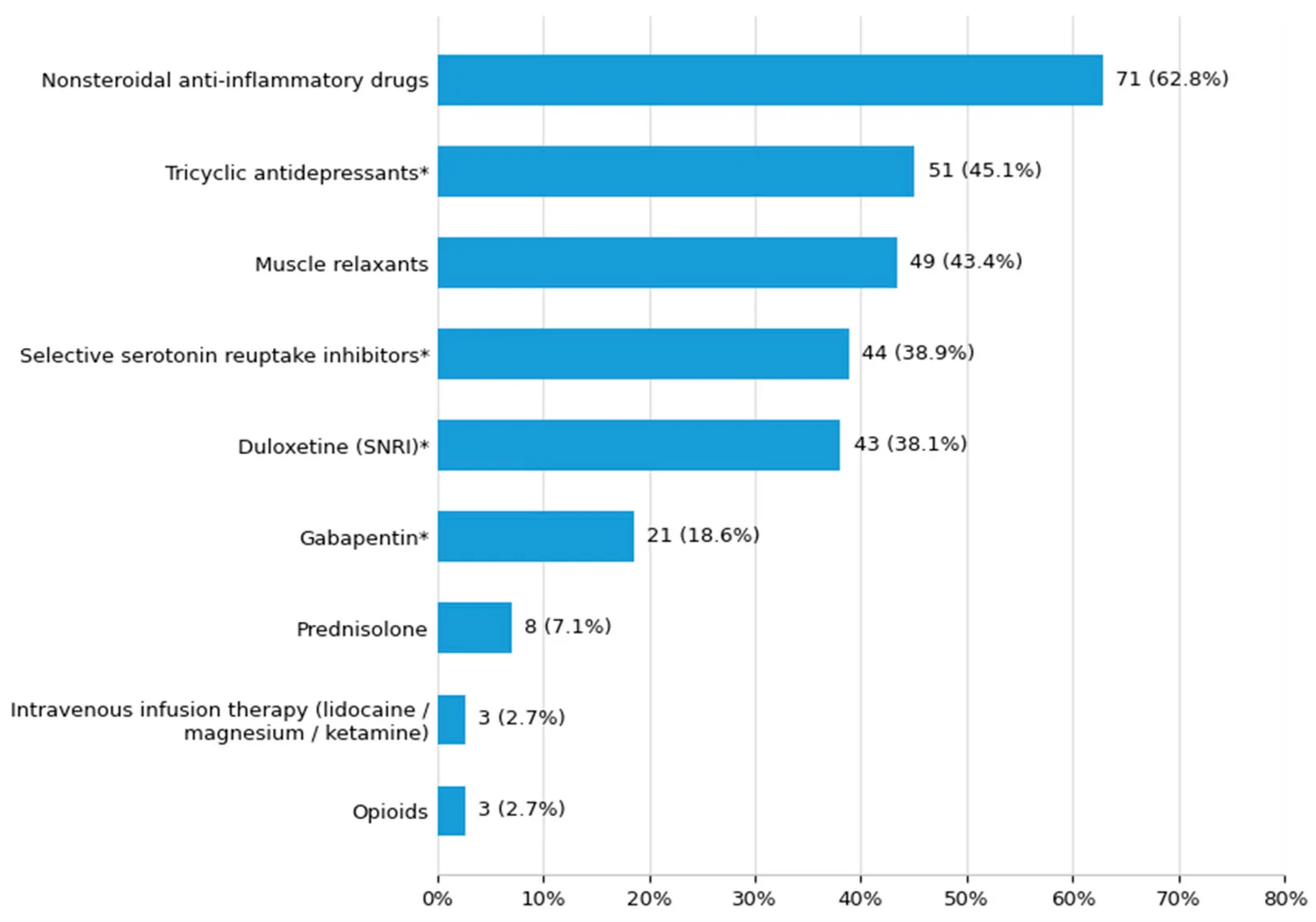
Pharmacological management of fibromyalgia. NSAIDs, non-steroidal anti-inflammatory drugs; SSRI, selective serotonin reuptake inhibitor; SNRI, serotonin–noradrenaline reuptake inhibitor. Duloxetine was presented to respondents as a separate option from the SSRI class. * Response keyed as correct.

**Table 1:** Socio-demographic characteristics of the study participants (n = 113).

Variable	Categories	n (%)
**Gender**	Male	65 (57.5)
	Female	48 (42.5)
**Age (years)**	<28	37 (32.7)
	28–35	43 (38.1)
	36–45	23 (20.4)
	46–55	4 (3.5)
	>55	6 (5.3)
**Nationality**	Saudi	105 (92.9)
	Non-Saudi	8 (7.1)
**Professional position**	Junior resident (R1–R2)	68 (60.2)
	Senior resident	14 (12.4)
	Consultant	31 (27.4)
**Clinical experience (years)**	<2	38 (33.6)
	2–5	32 (28.3)
	6–10	15 (13.3)
	11–15	17 (15)
	>15	11 (9.7)

**Table 2:** Knowledge of fibromyalgia among family medicine physicians and residents.

Item	Response Options	n (%)
Do you know the ACR criteria 1990, ACR 2010, or revised ACR 2016 for diagnosis of FM?	Yes *	45 (39.8)
	No	68 (60.2)
If you answered “Yes” to the above, did you use it to make a diagnosis? (n = 45)	Yes	30 (66.7)
	No	15 (33.3)
If you answered “No” to the above, why don’t you use it? (n = 68)	Complicated, hard to handle	11 (16.2)
	Hard to master	19 (27.9)
	Not reliable	13 (19.1)
	No reason	25 (36.8)
How many tender points were required by the ACR criteria 1990?	5/18	13 (11.5)
	11/18 *	66 (58.4)
	15/18	19 (16.8)
	16/18	15 (13.3)
Which of the following treatment strategies do you practise in your clinic?	Pharmacological	4 (3.5)
	Non-pharmacological	10 (8.8)
	Both treatments should be used simultaneously *	99 (87.6)
Which of the following non-pharmacological approaches do you practise in your clinic?	Physiotherapy *	62 (54.9)
	Cognitive behavioural therapy *	73 (64.6)
	Hydrotherapy	12 (10.6)
	Aerobic exercise *	50 (44.2)
	Graded physical exercise	50 (44.2)

* Correct answer. ACR, American College of Rheumatology; FM, fibromyalgia. Items marked with an asterisk contributed to the total knowledge score.

**Table 3:** Beliefs and practices regarding fibromyalgia among family medicine physicians and residents.

Item	Response Options	n (%)
1. Do you accept fibromyalgia as a separate and distinct clinical entity?	Yes *	94 (83.2)
	No	19 (16.8)
2. Do you think that fibromyalgia is primarily a psychological illness?	Yes	54 (47.8)
	No *	59 (52.2)
3. In your opinion, fibromyalgia is more prevalent in which sex?	Male	12 (10.6)
	Female *	101 (89.4)
4. Are you confident in recognising symptoms of fibromyalgia?	Yes *	56 (49.6)
	No	57 (50.4)
5. Are you confident in differentiating fibromyalgia from other similar diseases?	Yes *	45 (39.8)
	No	68 (60.2)
6. In your opinion, fibromyalgia is:	Appropriately diagnosed	4 (3.5)
	Missed *	57 (50.4)
	Over-diagnosed	6 (5.3)
	Under-diagnosed *	46 (40.7)
7. If you think it is over-, under- or missed diagnosed, what is the reason behind that in your opinion? (n = 109) †	Lack of knowledge	72 (66.1)
	Lack of training programmes	49 (45)
	Similarities to other diseases such as hypothyroidism and RA	63 (57.8)
	Other reasons (please specify)	5 (4.6)
8. If you answered “Other reasons”, please specify (n = 5) †	Diagnosed by exclusion with nonspecific symptoms and no objective investigations	3 (60)
	Not widely discussed	1 (20)
	Patient refusal of diagnosis	1 (20)
9. Where have you learned most about fibromyalgia? †	CME programmes	9 (8)
	Post-graduation training	71 (62.8)
	Undergraduate knowledge	33 (29.2)
10. Which of the following services would best manage patients with fibromyalgia? †	Family medicine physician	20 (17.7)
	Multidisciplinary team	54 (47.8)
	Neurologist	1 (0.9)
	Pain management physician	8 (7.1)
	Psychiatrist	5 (4.4)
	Rheumatologist	25 (22.1)

* Indicates the response scored as positive. Items 1–6 contributed to the total belief score (maximum 6); items marked † were descriptive and were not scored. Items 4 and 5 assessed self-rated confidence (self-efficacy) rather than knowledge. CME, continuing medical education; RA, rheumatoid arthritis.

**Table 4:** Association between demographic factors and physicians’ knowledge and beliefs about fibromyalgia.

Variable	n	Knowledge Score Mean ± SD	Test Statistic	*p* Value	Belief Score Mean ± SD	Test Statistic	*p* Value
**Gender:**							
Male	65	9.3 ± 3.19	t (111) = −1.146	0.254	4.1 ± 1.28	t (111) = 0.547	0.586
Female	48	10.1 ± 3.49			4.0 ± 1.18		
**Age (years):**							
<28	37	9.4 ± 3.08	F (4, 108) = 0.503	0.733	3.9 ± 1.11	F (4, 108) = 1.254	0.293
28–35	43	9.7 ± 3.67			4.1 ± 1.31		
36–45	23	10.0 ± 3.25			4.1 ± 1.28		
46–55	4	11.3 ± 4.57			5.3 ± 0.96		
>55	6	8.5 ± 1.64			3.8 ± 1.17		
**Nationality:**							
Saudi	105	9.8 ± 3.33	t (111) = 1.235	0.219	4.0 ± 1.23	t (111) = −1.066	0.289
Non-Saudi	8	8.3 ± 3.20			4.5 ± 1.20		
**Professional position:**							
Junior resident	68	9.2 ± 3.26	F (2, 110) = 2.228	0.113	3.9 ± 1.16	F (2, 110) = 1.773	0.175
Senior resident	14	10.9 ± 3.77			4.6 ± 1.28		
Consultant	31	10.2 ± 3.12			4.1 ± 1.34		
**Clinical experience:**							
<2 years	38	9.5 ± 2.85	F (4, 108) = 0.315	0.867	4.0 ± 1.18	F (4, 108) = 0.163	0.957
2–5 years	32	9.7 ± 3.97			4.0 ± 1.20		
6–10 years	15	9.9 ± 3.58			4.2 ± 1.47		
11–15 years	17	10.2 ± 3.53			4.1 ± 1.27		
>15 years	11	8.9 ± 2.39			4.2 ± 1.25		
**Hospital type:**							
Academic and training	28	10.1 ± 3.28	t (111) = 0.779	0.437	4.7 ± 1.19	t (111) = 3.228	0.002
Other centres	85	9.5 ± 3.35			3.9 ± 1.18		

Reference group: gender (male), age group (< 28 years), nationality (Saudi), position (consultants), experience (< 2 years), hospital type (other centres). SD, standard deviation.

**Table 5:** Multivariable linear regression analysis of factors associated with knowledge score, with collinearity statistics.

Variable	B	95% CI for B	*p* Value	Tolerance	VIF
**Female gender**	1.22	−0.15 to 2.58	0.080	0.799	1.25
**Age group:**					
**28–35 years**	0.01	−2.19 to 2.21	0.995	0.320	3.12
**36–45 years**	1.34	−2.78 to 5.46	0.520	0.132	7.55
**46–55 years**	3.98	−1.58 to 9.55	0.159	0.345	2.90
**>55 years**	1.74	−4.40 to 7.88	0.575	0.192	5.20
**Non-Saudi nationality**	−0.96	−4.10 to 2.18	0.544	0.562	1.78
**Professional position:**					
**Junior resident**	−4.24	−7.56 to −0.93	0.013	0.139	7.22
**Senior resident**	−0.66	−3.56 to 2.24	0.655	0.400	2.50
**Clinical experience:**					
**2–5 years**	−0.15	−2.34 to 2.03	0.889	0.378	2.65
**6–10 years**	−3.37	−6.92 to 0.18	0.063	0.251	3.98
**11–15 years**	−4.53	−9.52 to 0.46	0.075	0.115	8.73
**>15 years**	−6.87	−12.81 to −0.92	0.024	0.118	8.50
**Academic hospital**	0.98	−0.79 to 2.76	0.275	0.622	1.61

Reference group: gender (male), age group (<28 years), nationality (Saudi), position (consultant), experience (<2 years), hospital type (other centres). B, unstandardised regression coefficient; CI, confidence interval; VIF, variance inflation factor. The model as a whole was not statistically significant: F (13, 99) = 1.476, *p* = 0.140; *R*^2^ = 0.162; adjusted *R*^2^ = 0.052. Coefficients for junior resident status and >15 years of experience did not remain significant in sensitivity models omitting the collinear variable (see Section 3).

## Data Availability

The data presented in this study are available on reasonable request from the corresponding author. The data are not publicly available because they contain professional information that could compromise the privacy of individual participants.

## Supplementary Materials

The following supporting information can be downloaded at: https://www.mdpi.com/article/10.3390/healthcare14193201/s1, Section S1: STROBE checklist for cross-sectional studies; Section S2: CHERRIES checklist for internet e-surveys; Section S3: Normality and distributional diagnostics (IBM SPSS output); Section S4: Multivariable regression output, collinearity and residual diagnostics, and sensitivity models (IBM SPSS output).

## Abbreviations

The following abbreviations are used in this manuscript:

ACR	American College of Rheumatology
ANOVA	Analysis of variance
CHERRIES	Checklist for Reporting Results of Internet E-Surveys
CI	Confidence interval
CME	Continuing medical education
EULAR	European Alliance of Associations for Rheumatology
FM	Fibromyalgia
NSAID	Non-steroidal anti-inflammatory drug
SD	Standard deviation
SNRI	Serotonin–noradrenaline reuptake inhibitor
SSRI	Selective serotonin reuptake inhibitor
STROBE	Strengthening the Reporting of Observational Studies in Epidemiology
VIF	Variance inflation factor
