# Supplementary material for: Knowledge, Beliefs, and Practices Regarding Fibromyalgia Among Family Medicine Physicians and Residents in Saudi Arabia: A Cross-Sectional Study
Source: Healthcare (Basel). 2026 Sep 28;14(19):3201. doi: 10.3390/healthcare14193201 (PMC13664930; doi:10.3390/healthcare14193201)

Supplementary Materials

Knowledge, Beliefs, and Practices Regarding Fibromyalgia Among Family Medicine Physicians and Residents in Saudi Arabia: A Cross-Sectional Study (healthcare-4543679)

This document combines all four supplementary files cited in the manuscript.

File	Content	Pages in this document
**S1**	STROBE checklist for cross-sectional studies	2–4
**S2**	CHERRIES checklist for internet e-surveys	5–7
**S3**	Normality and distributional diagnostics (IBM SPSS output)	8–59
**S4**	Multivariable regression output, collinearity and residual diagnostics, and sensitivity models (IBM SPSS output)	60–82

Supplementary File S1. STROBE Statement — checklist for cross-sectional studies

Manuscript ID healthcare-4543679. “Knowledge, Beliefs, and Practices Regarding Fibromyalgia Among Family Medicine Physicians and Residents in Saudi Arabia: A Cross-Sectional Study”. Page numbers refer to the revised manuscript submitted with this checklist. Where an item could not be reported, the reason is stated rather than the item being marked as addressed.

Item	Recommendation topic	How it is addressed in the manuscript	Location (revised manuscript)
**TITLE AND ABSTRACT**			
1a	Study design in title or abstract	The cross-sectional design is stated in the title and in the first sentence of the Abstract Methods.	Title, p. 1; Abstract, p. 1
1b	Informative and balanced abstract	Structured abstract (Background / Methods / Results / Conclusions) reporting design, setting, period, population, scoring, analysis, principal findings with SD and 95% CI, and a conclusion restricted to the convenience sample.	Abstract, p. 1
**INTRODUCTION**			
2	Background / rationale	Burden and underdiagnosis of FM, evolution of the ACR criteria, guideline-recommended management, documented knowledge gaps regionally and internationally, and the gap in Saudi family-medicine-specific data.	Section 1, p. 2
3	Objectives, including prespecified hypotheses	Study aim stated, followed by three explicit research questions. It is stated that the study was exploratory and that no formal a priori hypotheses were prespecified.	Section 1, p. 2, final paragraph
**METHODS**			
4	Study design	Quantitative, analytical cross-sectional study; stated at the start of the Methods.	Section 2.1, p. 2
5	Setting, locations, dates	MOH, military, academic and primary healthcare facilities in Saudi Arabia; recruitment period July–December 2025; online distribution centred on Riyadh-based networks.	Sections 2.1 and 2.3, pp. 2–3
6a	Eligibility criteria, sources and methods of selection	Family medicine physicians and residents of both sexes and all nationalities practising in Saudi Arabia; consultant, senior resident and junior resident defined explicitly; only non-consenting individuals excluded. Coverage limitation of online-only distribution stated separately from eligibility.	Section 2.2, p. 2
7	Variables	Outcome variables (knowledge score, belief score) and all six explanatory variables defined, with reference categories specified.	Sections 2.4 and 2.6, p. 3
8	Data sources / measurement	Self-administered online questionnaire (Google Forms) adapted from a previously published English-language instrument; content of each section described; scoring rule and maxima stated; validity and reliability status reported; full item	Section 2.4, p. 3; Tables 2–3; Figures 2–3

Item	Recommendation topic	How it is addressed in the manuscript	Location (revised manuscript)
		wording reproduced in Tables 2–3 and Figures 2–3.	
9	Bias	Dedicated subsection naming self-selection bias, geographic non-representativeness and social desirability bias; non-response bias addressed in the limitations.	Section 2.3, p. 3; Section 4.3, p. 12
10	Study size	Single-proportion calculation (95% confidence, 5% margin of error, p = 0.5) giving a target of 384; achieved sample of 113 reported as 29.4% of target.	Section 2.5, p. 3
11	Quantitative variables	Knowledge and belief scores handled as continuous sums; categorical predictors grouped as shown in Table 1; no post hoc categorisation of scores, and the absence of validated cut-offs is stated.	Sections 2.4 and 2.6, p. 3
12a	Statistical methods	Descriptive statistics; independent-samples t-test and one-way ANOVA; multivariable linear regression with reference categories specified; distributional assumptions described (within-group for t-test/ANOVA, residuals for regression).	Section 2.6, p. 3
12b	Subgroup / interaction analyses	None performed; results are presented for the whole sample with stratified descriptive comparisons only.	Section 2.6, p. 3
12c	Missing data	No missing values were present for any analysed variable (SPSS case-processing summaries, Supplementary Files S3 and S4); no imputation was performed.	Supplementary Files S3 and S4
12d	Sampling strategy	Convenience sampling through open online distribution; addressed as a limitation.	Sections 2.3 and 2.5, p. 3; Section 4.3, p. 12
12e	Sensitivity analyses	None performed in this version. Collinearity diagnostics and a sensitivity analysis excluding one of the two collinear variables are offered to the editorial office (see Reply to Reviewer 4, Response 4).	Section 4.3, p. 12
**RESULTS**			
13a–c	Participants	113 participants enrolled, corresponding to 29.4% of the calculated target. Because the survey was an open forwardable link, the number of individuals approached or viewing the link cannot be established and no flow diagram or response rate can be constructed; this is stated explicitly.	Section 3.1, p. 4; Section 2.3, p. 3
14a	Descriptive data	Sex, age group, nationality, professional position, years of clinical experience and facility type reported for all 113 participants.	Section 3.1, p. 4; Table 1, p. 4; Figure 1, p. 5
14b	Missing data for each variable	No missing data; all submitted responses were complete.	Section 3.1, p. 4

Item	Recommendation topic	How it is addressed in the manuscript	Location (revised manuscript)
15	Outcome data	Item-level frequencies for all knowledge, belief and practice items; mean knowledge and belief scores with SD and percentage of maximum.	Sections 3.2–3.4, pp. 5–8; Tables 2–3; Figures 2–3
16a	Main results	Unadjusted comparisons with test statistics and p values (Table 4); multivariable coefficients with 95% CI and p values, and reference categories stated (Table 5).	Section 3.4, pp. 8–10; Tables 4–5
16b	Category boundaries	Age, experience and position categories reported as collected; score categories deliberately not created, as no validated cut-offs exist.	Table 1, p. 4; Section 2.4, p. 3
17	Other analyses	None beyond those reported.	Section 3.4, pp. 8–10
**DISCUSSION**			
18	Key results	Summarised with reference to the three research questions in the opening paragraph of the Discussion.	Section 4, p. 11
19	Limitations	Eight limitations reported: sample shortfall and power; non-response bias; convenience sampling and Riyadh concentration; self-report; absence of psychometric validation and equal item weighting; collinearity of position and experience; training-stage composition of the sample; cross-sectional design and absence of causal inference.	Section 4.3, p. 12
20	Interpretation	Findings interpreted against contemporary Arab-region and international comparators; implications framed as directions rather than established conclusions.	Section 4, pp. 11–12; Sections 4.1–4.2, p. 12
21	Generalisability	Explicitly stated that the findings describe a convenience sample and should not be generalised to Saudi family medicine as a whole.	Section 4.3 and Section 5, p. 12
**OTHER INFORMATION**			
22	Funding	Stated: no external funding received.	Funding statement, p. 13

STROBE: von Elm E, Altman DG, Egger M, Pocock SJ, Gøtzsche PC, Vandenbroucke JP; STROBE Initiative. The Strengthening the Reporting of Observational Studies in Epidemiology (STROBE) statement: guidelines for reporting observational studies. Lancet 2007;370:1453–1457.

Supplementary File S2. CHERRIES checklist for reporting results of internet e-surveys

Manuscript ID healthcare-4543679. “Knowledge, Beliefs, and Practices Regarding Fibromyalgia Among Family Medicine Physicians and Residents in Saudi Arabia: A Cross-Sectional Study”. Page numbers refer to the revised manuscript submitted with this checklist. Items that could not be reported for this survey are marked as such, with the reason given.

CHERRIES item	Response for this study	Location (revised manuscript)
**DESIGN**		
Describe survey design	Cross-sectional, self-administered web-based survey of family medicine physicians and residents; convenience sampling through open online distribution.	Sections 2.1, 2.3 and 2.5, pp. 2–3
**IRB APPROVAL AND INFORMED CONSENT PROCESS**		
IRB approval	Institutional Review Board of King Saud University, Riyadh (approval number E-25-9895; 26 June 2025).	Section 2.7, p. 4; IRB statement, p. 13
Informed consent	Electronic informed consent obtained from every participant before access to the questionnaire; those declining consent were excluded.	Sections 2.2 and 2.7, pp. 2, 4
Data protection	Responses stored in a protected electronic format accessible only to the research team; data held confidentially and anonymously and used solely for research.	Sections 2.4 and 2.7, pp. 3–4
**DEVELOPMENT AND PRE-TESTING**		
Development and testing	Adapted from a previously published English-language instrument; no items added, removed or reworded; adaptation limited to sociodemographic response categories. Reviewed before distribution by the research team (including a consultant rheumatologist and consultant anaesthetists with a chronic pain practice) for content and face validity. No psychometric revalidation was performed; no pilot testing is reported. Reported as a limitation.	Section 2.4, p. 3; Section 4.3, p. 12
**RECRUITMENT PROCESS AND DESCRIPTION OF THE SAMPLE**		
Open vs closed survey	Open survey. The link was not restricted to a pre-specified invitation list and could be forwarded.	Section 2.3, p. 3
Contact mode	Participants were reached through online professional networks and social media groups of family medicine physicians and residents.	Section 2.3, p. 3
Advertising the survey	Disseminated through professional networks and social media groups, with dissemination centred on Riyadh-based networks. Individual platforms are not itemised in the manuscript.	Section 2.3, p. 3
**SURVEY ADMINISTRATION**		
Web / e-mail	Web-based; questionnaire hosted on Google Forms.	Section 2.4, p. 3

CHERRIES item	Response for this study	Location (revised manuscript)
Context	Study aim explained on the introductory interface of the form.	Section 2.4, p. 3
Mandatory / voluntary	Voluntary. Participants were free to withdraw at any point without consequence.	Sections 2.3 and 2.7, pp. 3–4
Incentives	None offered.	Sections 2.3 and 2.7, pp. 3–4
Time / date	July to December 2025.	Section 2.1, p. 2
Randomisation of items or questionnaires	Not used; items were presented in a fixed order.	Section 2.4, p. 3
Adaptive questioning	Used for two conditional items only (use of the ACR criteria, asked of those aware of them, n = 45; reasons for non-use, n = 68), and for the free-text follow-up to the “other reasons” option (n = 5). Denominators are stated in the tables.	Tables 2–3, pp. 5–8
Number of items and screens	Three sections (sociodemographic, knowledge, belief and practice) comprising 18 scored knowledge items and 6 scored belief items plus descriptive items; full item wording reproduced in Tables 2–3 and Figures 2–3.	Section 2.4, p. 3; Tables 2–3; Figures 2–3
Completeness check / review step	No missing values were present for any analysed variable (Supplementary Files S3 and S4). Whether respondents could review answers before submission was not documented.	Supplementary Files S3 and S4
**RESPONSE RATES**		
View rate, participation rate, completion rate	Not calculable. Because the link was open and forwardable, the number of individuals who viewed the first page is unknown; this is stated explicitly in the Methods and revisited in the limitations. The achieved sample of 113 is reported against the calculated target of 384 (29.4%).	Sections 2.3 and 2.5, p. 3; Section 4.3, p. 12
**PREVENTING MULTIPLE ENTRIES FROM THE SAME INDIVIDUAL**		
Cookies, IP check, log file analysis, registration	No technical duplicate-prevention measure is documented. Multiple submissions by the same respondent could not be excluded with certainty; stated in the Methods.	Section 2.3, p. 3
**ANALYSIS**		
Handling of incomplete questionnaires	Not applicable; no missing values were present for any analysed variable, and no imputation was required.	Supplementary Files S3 and S4
Questionnaires with atypical timestamps	No responses were excluded; all 113 were analysed.	Tables 1–5
Statistical correction	None applied. No weighting or propensity adjustment was used; the resulting limitations on representativeness are stated in full.	Section 2.6, p. 3; Section 4.3, p. 12

CHERRIES: Eysenbach G. Improving the quality of web surveys: the Checklist for Reporting Results of Internet E-Surveys (CHERRIES). J Med Internet Res 2004;6(3):e34. doi:10.2196/jmir.6.3.e34

```
EXAMINE VARIABLES=Knowledge_score Attitude_score
  /PLOT HISTOGRAM NPPLOT
  /STATISTICS DESCRIPTIVES
  /CINTERVAL 95
  /MISSING LISTWISE
  /NOTOTAL.
```

Explore

Notes

Output Created		29-MAR-2026 10:51:34
Comments		
Input	Data	C:\Users\Mosaab Lap\Desktop\SPSS Files\SPSS 419\Data 419.sav
	Active Dataset	DataSet3
	Filter	<none>
	Weight	<none>
	Split File	<none>
	N of Rows in Working Data File	113
Missing Value Handling	Definition of Missing	User-defined missing values for dependent variables are treated as missing.
	Cases Used	Statistics are based on cases with no missing values for any dependent variable or factor used.
Syntax		EXAMINE VARIABLES=Knowledge_score Attitude_score<br>/PLOT HISTOGRAM NPPLOT<br>/STATISTICS DESCRIPTIVES<br>/CINTERVAL 95<br>/MISSING LISTWISE<br>/NOTOTAL.
Resources	Processor Time	00:00:01.41
	Elapsed Time	00:00:11.51

Case Processing Summary

	Cases					
	Valid		Missing		Total	
	N	Percent	N	Percent	N	Percent
Knowledge_score	113	100.0%	0	0.0%	113	100.0%
Attitude_score	113	100.0%	0	0.0%	113	100.0%

Descriptives

			Statistic	Std. Error
Knowledge_score	Mean		9.65	.313
	95% Confidence Interval for Mean	Lower Bound	9.03	
		Upper Bound	10.27	
	5% Trimmed Mean		9.60	
	Median		9.00	
	Variance		11.052	
	Std. Deviation		3.324	
	Minimum		3	
	Maximum		18	
	Range		15	
	Interquartile Range		5	
	Skewness		.364	.227
	Kurtosis		-.344-	.451
Attitude_score	Mean		4.05	.116
	95% Confidence Interval for Mean	Lower Bound	3.82	
		Upper Bound	4.28	
	5% Trimmed Mean		4.07	
	Median		4.00	
	Variance		1.515	
	Std. Deviation		1.231	
	Minimum		1	
	Maximum		6	
	Range		5	
	Interquartile Range		2	
	Skewness		.014	.227
	Kurtosis		-.823-	.451

Tests of Normality

	Kolmogorov-Smirnov[a]			Shapiro-Wilk		
	Statistic	df	Sig.	Statistic	df	Sig.
Knowledge_score	.108	113	.002	.971	113	.014
Attitude_score	.176	113	.000	.919	113	.000

a. Lilliefors Significance Correction

Knowledge_score

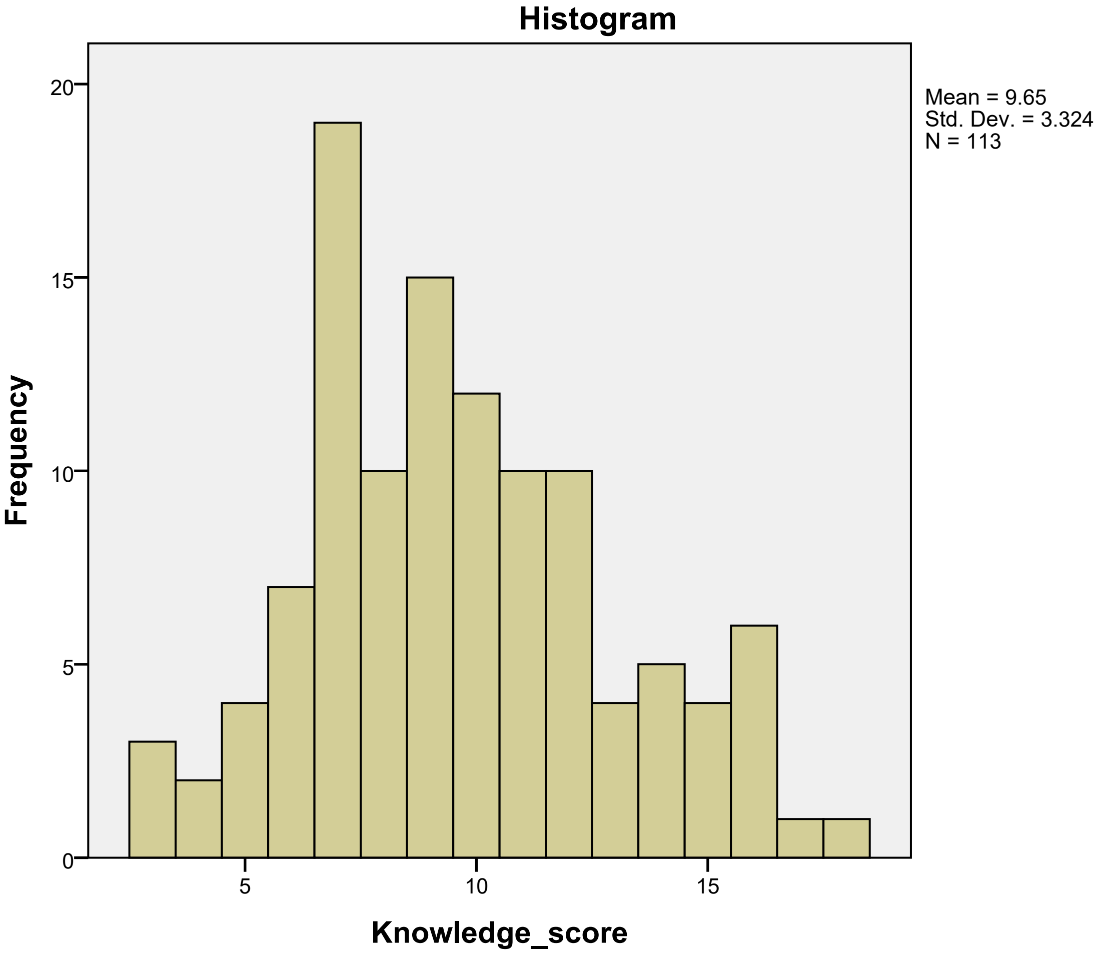
Histogram
Mean = 9.65
Std. Dev. = 3.324
N = 113
Frequency
Knowledge_score
0
5
10
15
20
5
10
15

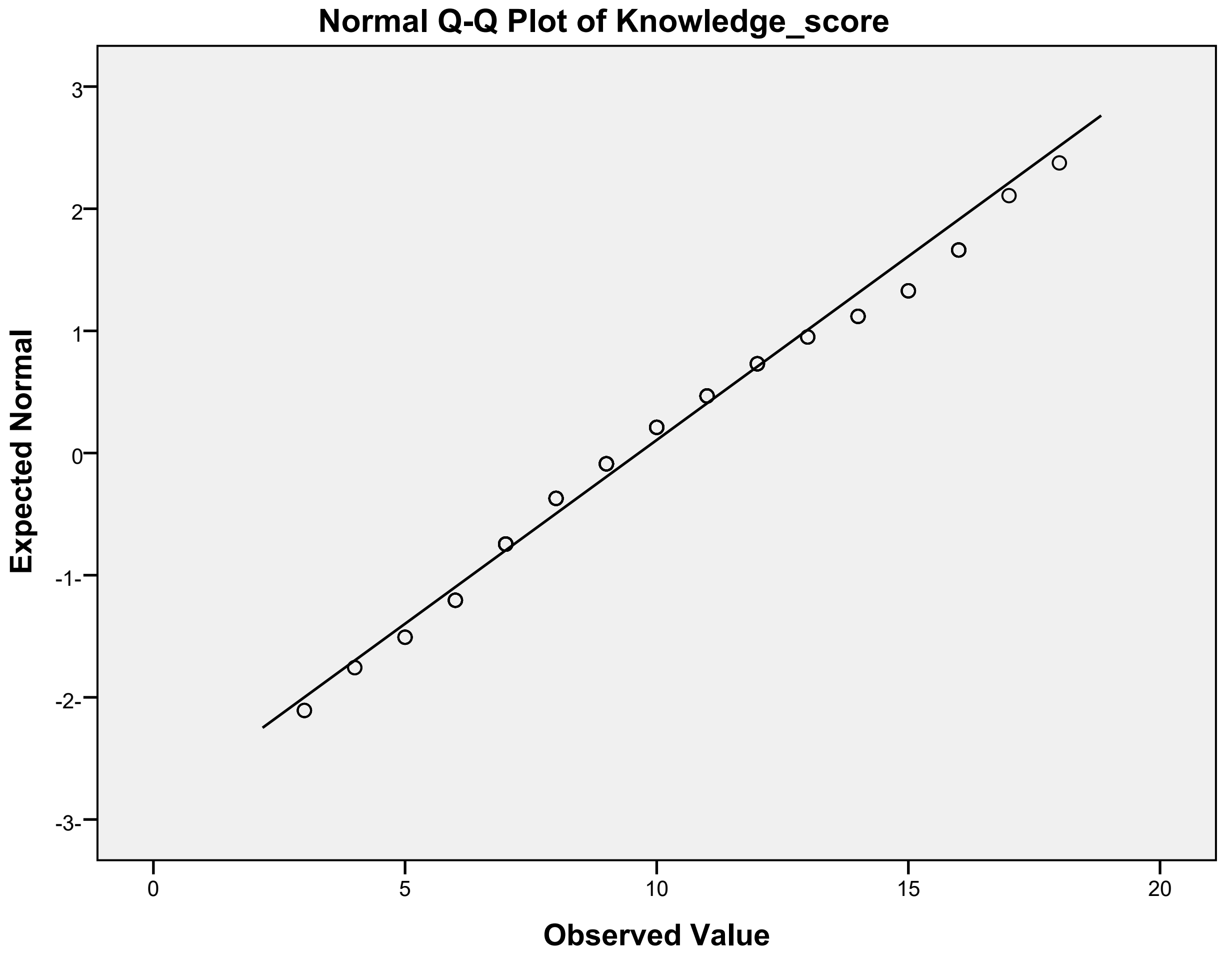
Normal Q-Q Plot of Knowledge_score
Expected Normal
Observed Value
3
2
1
0
-1
-2
-3
0
5
10
15
20

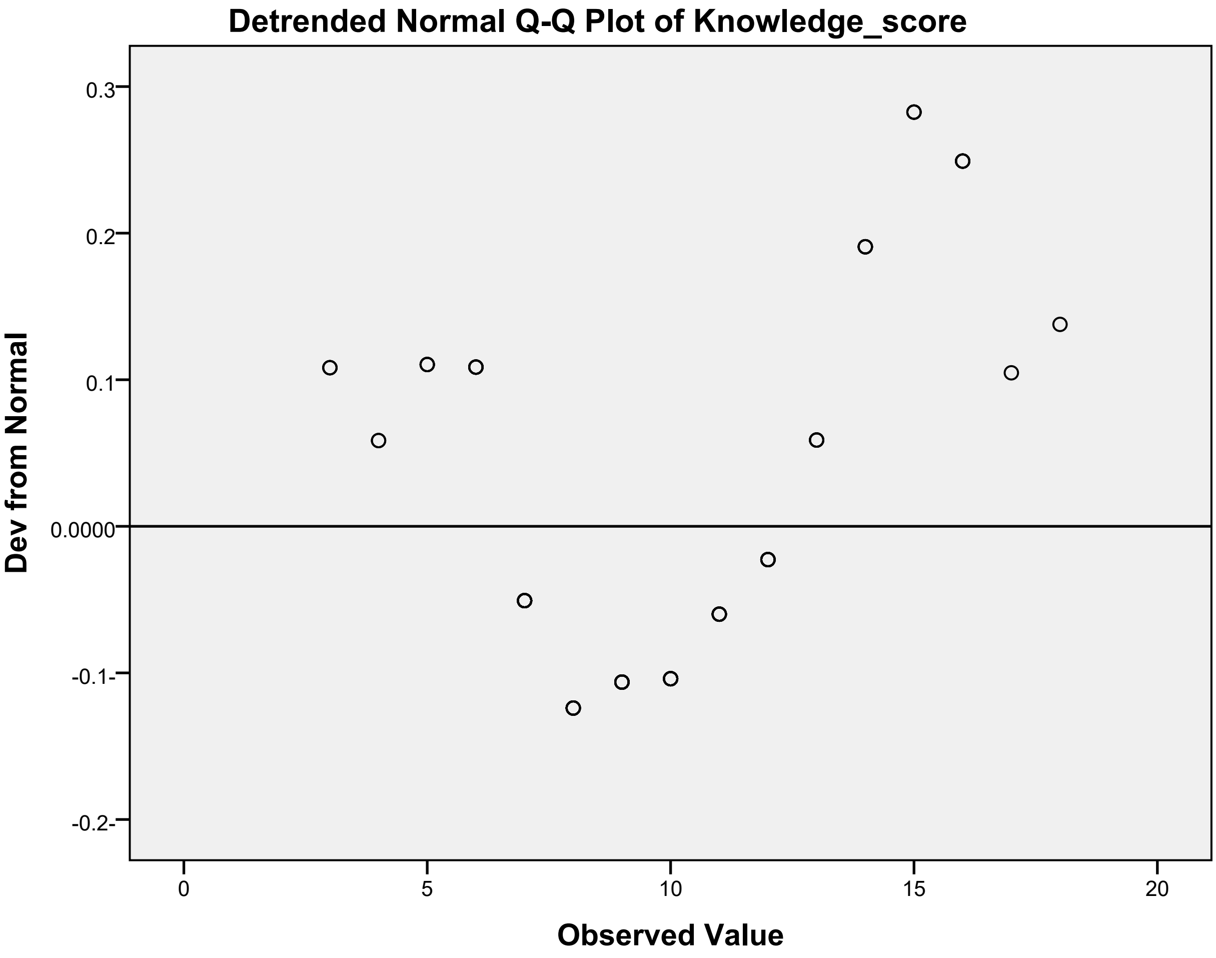

Attitude_score

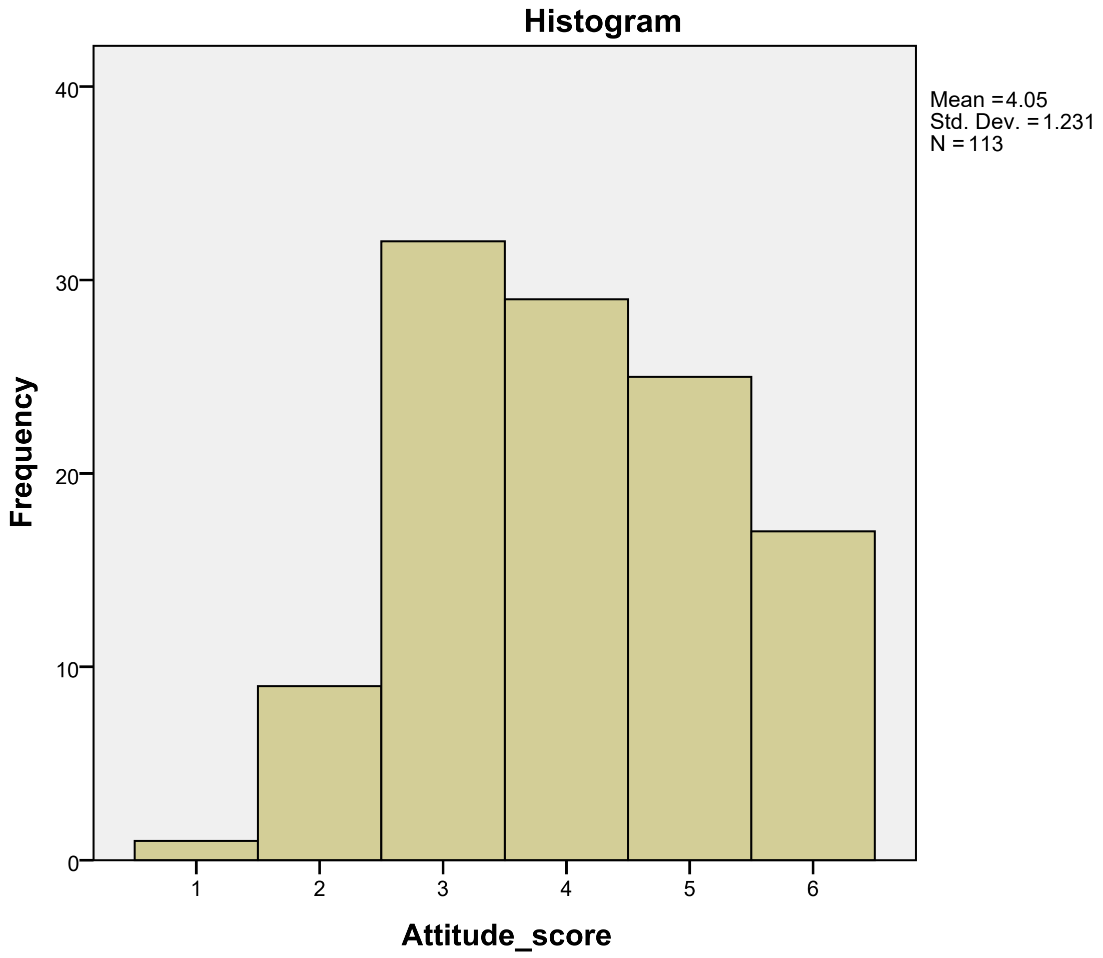
Histogram
40
30
20
10
0
Frequency
1
2
3
4
5
6
Attitude_score
Mean =4.05
Std. Dev. =1.231
N =113

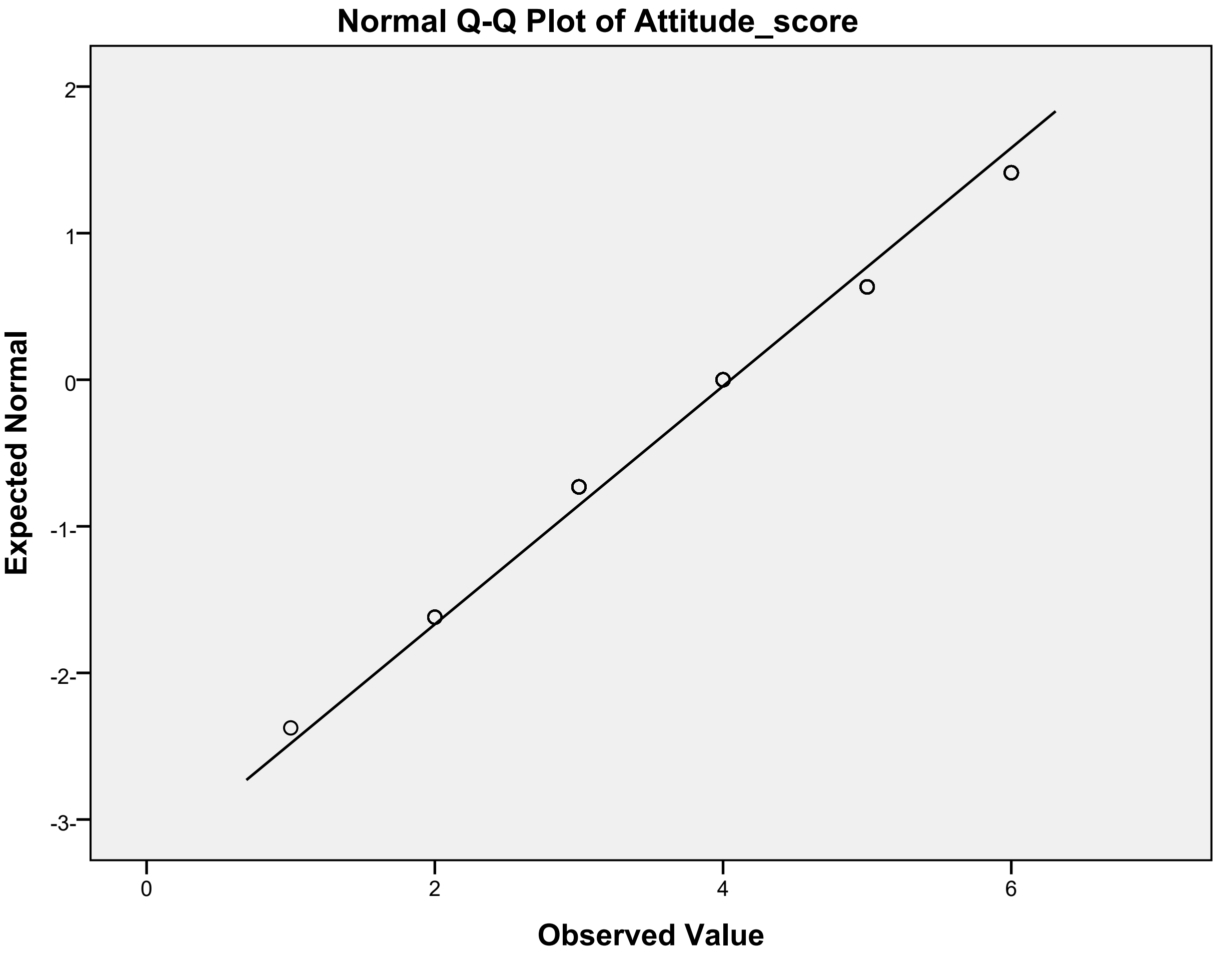

Normal Q-Q Plot of Attitude_score
Expected Normal
2
1
0
-1
-2
-3
0
2
4
6
Observed Value

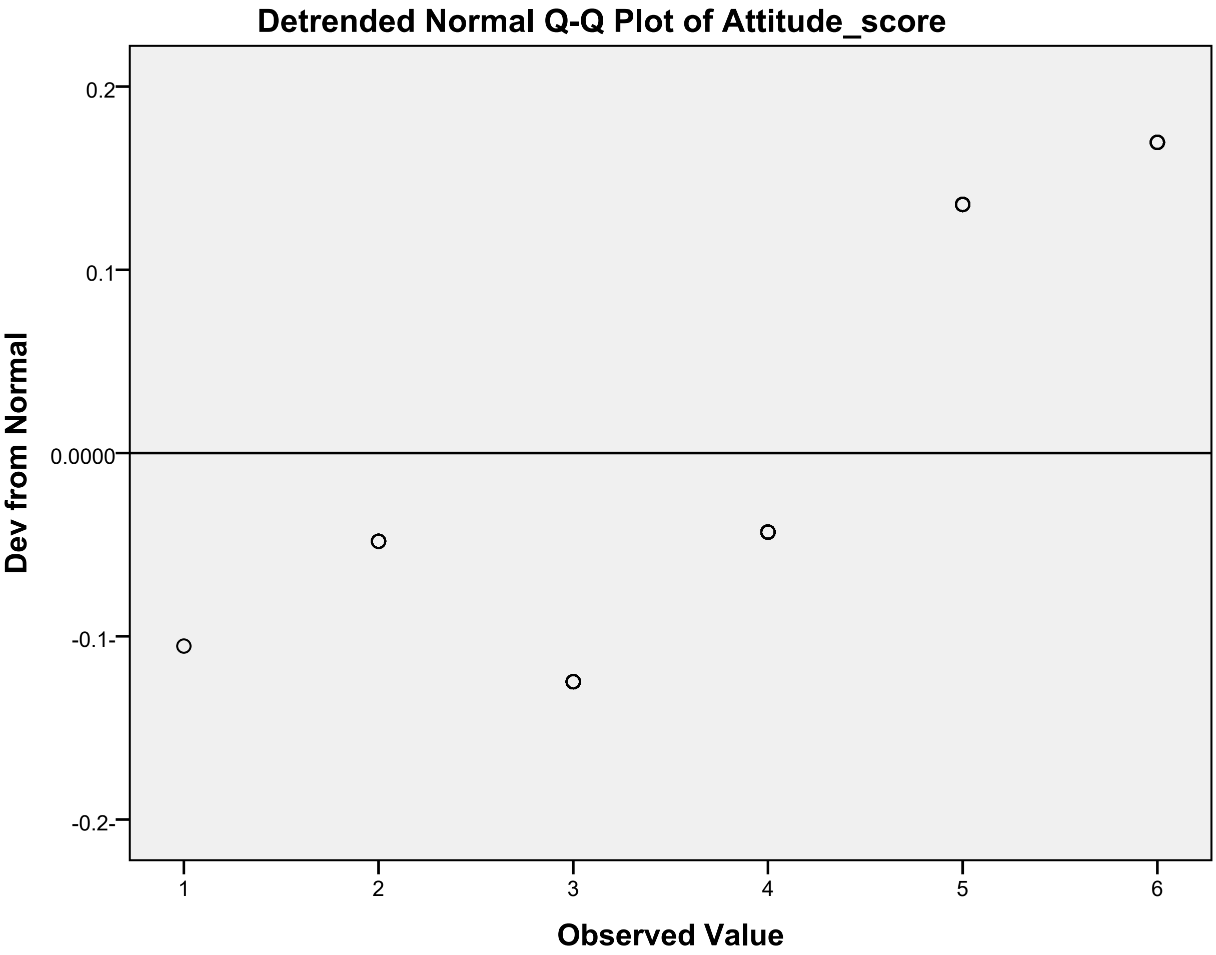
Detrended Normal Q-Q Plot of Attitude_score
Dev from Normal
0.2
0.1
0.0000
-0.1
-0.2
1
2
3
4
5
6
Observed Value

```
EXAMINE VARIABLES=Knowledge_score BY Gender Nationality hospital_cat2
  /PLOT HISTOGRAM NPPLOT
  /STATISTICS DESCRIPTIVES
  /CINTERVAL 95
  /MISSING LISTWISE
  /NOTOTAL.
```

Explore

Notes

Output Created		19-SEP-2026 23:51:11
Comments		
Input	Data	C:\Users\Mosaab Lap\Desktop\SPSS Files\SPSS 419\Data 419.sav
	Active Dataset	DataSet6
	Filter	<none>
	Weight	<none>
	Split File	<none>
	N of Rows in Working Data File	113
Missing Value Handling	Definition of Missing	User-defined missing values for dependent variables are treated as missing.
	Cases Used	Statistics are based on cases with no missing values for any dependent variable or factor used.
Syntax		EXAMINE VARIABLES=Knowledge_score BY Gender Nationality hospital_cat2 /PLOT HISTOGRAM NPPLOT /STATISTICS DESCRIPTIVES /CINTERVAL 95 /MISSING LISTWISE /NOTOTAL.
Resources	Processor Time	00:00:01.39
	Elapsed Time	00:00:01.22

Gender

Case Processing Summary

	Gender	Cases					
		Valid		Missing		Total	
		N	Percent	N	Percent	N	Percent
Knowledge_score	Male	65	100.0%	0	0.0%	65	100.0%
	Female	48	100.0%	0	0.0%	48	100.0%

Descriptives

	Gender			Statistic	Std. Error
Knowledge_score	Male	Mean		9.34	.395
		95% Confidence Interval for Mean	Lower Bound	8.55	
			Upper Bound	10.13	
		5% Trimmed Mean		9.28	
		Median		9.00	
		Variance		10.165	
		Std. Deviation		3.188	
		Minimum		3	
		Maximum		17	
		Range		14	
		Interquartile Range		4	
		Skewness		.368	.297
		Kurtosis		-.252-	.586
	Female	Mean		10.06	.504
		95% Confidence Interval for Mean	Lower Bound	9.05	
			Upper Bound	11.08	
		5% Trimmed Mean		10.04	
		Median		10.00	
		Variance		12.188	
		Std. Deviation		3.491	
		Minimum		3	
		Maximum		18	
		Range		15	
		Interquartile Range		5	
		Skewness		.321	.343
		Kurtosis		-.436-	.674

Tests of Normality

	Gender	Kolmogorov-Smirnov[a]			Shapiro-Wilk		
		Statistic	df	Sig.	Statistic	df	Sig.
Knowledge_score	Male	.124	65	.014	.969	65	.103
	Female	.153	48	.007	.968	48	.219

a. Lilliefors Significance Correction

Knowledge_score

Histograms

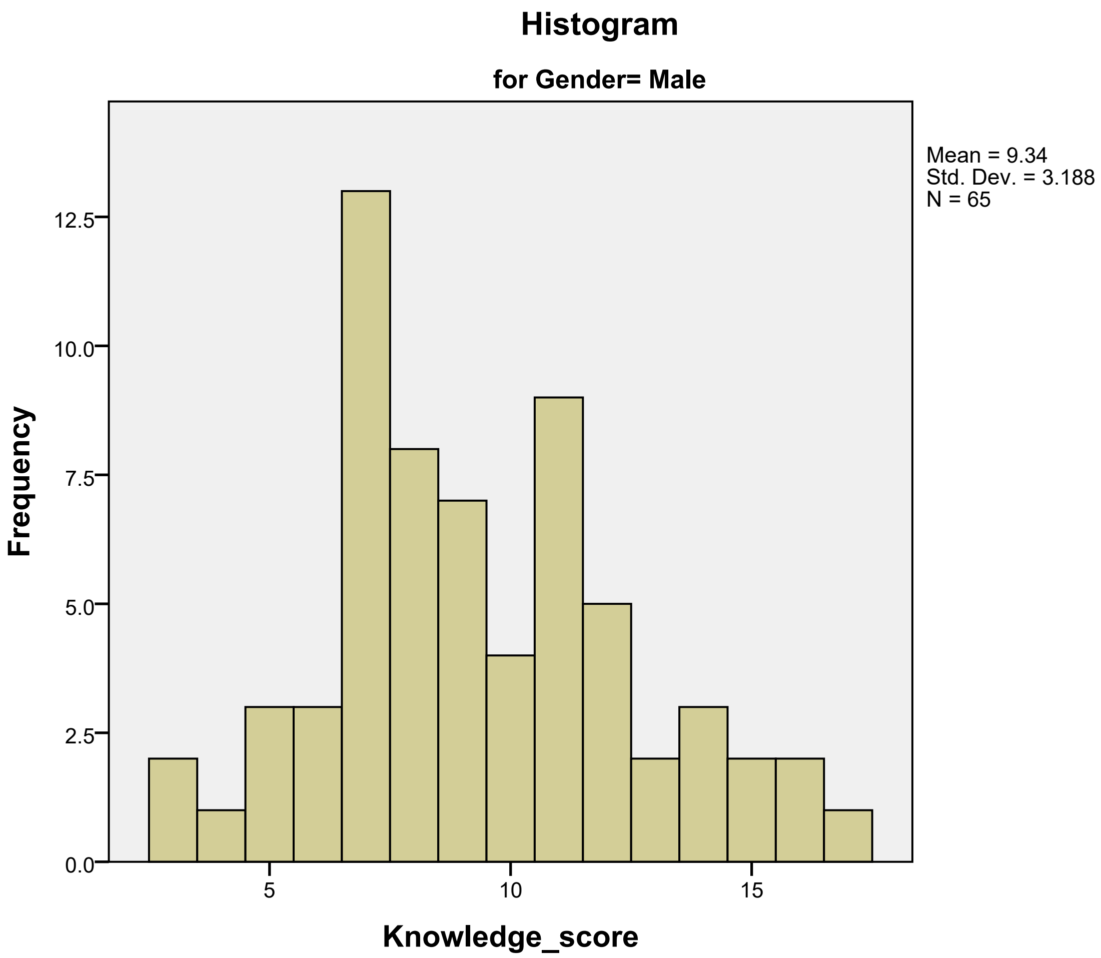

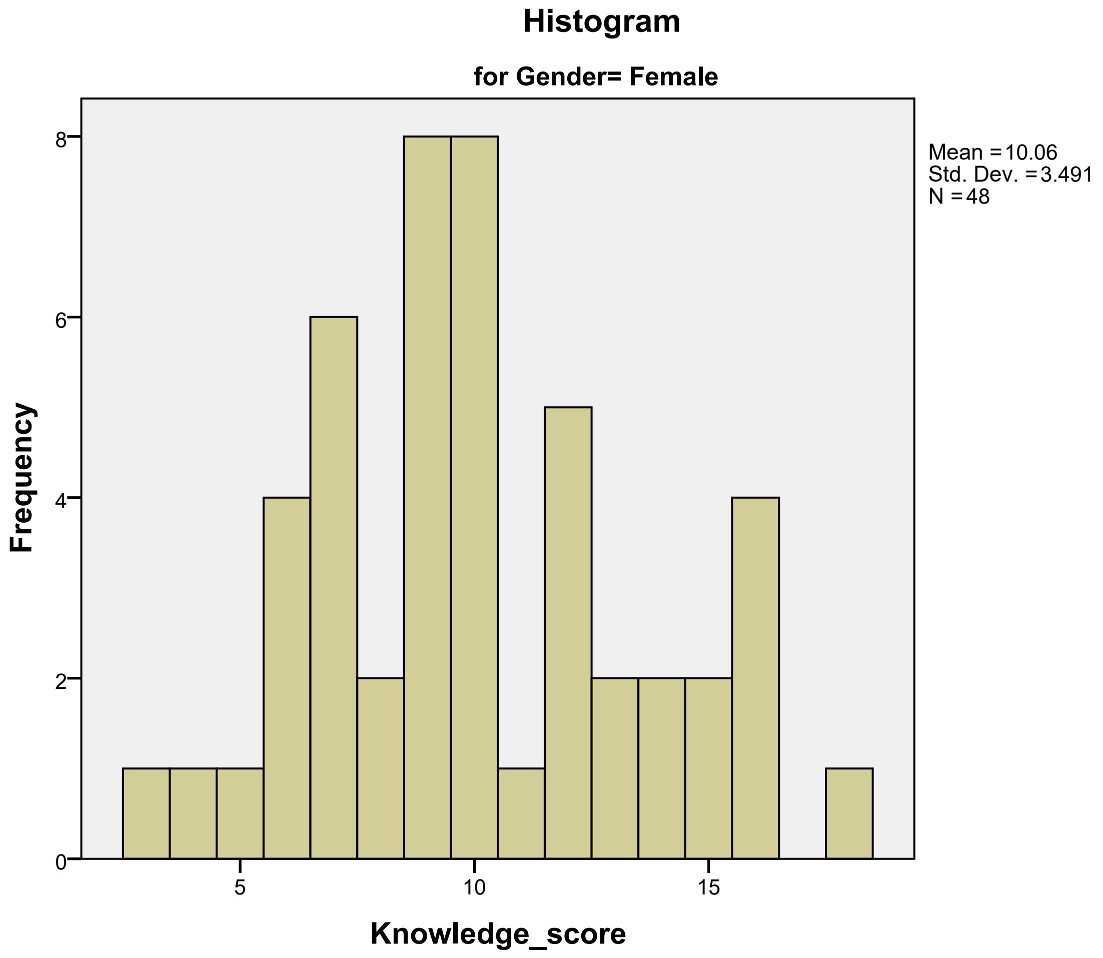

Normal Q-Q Plots

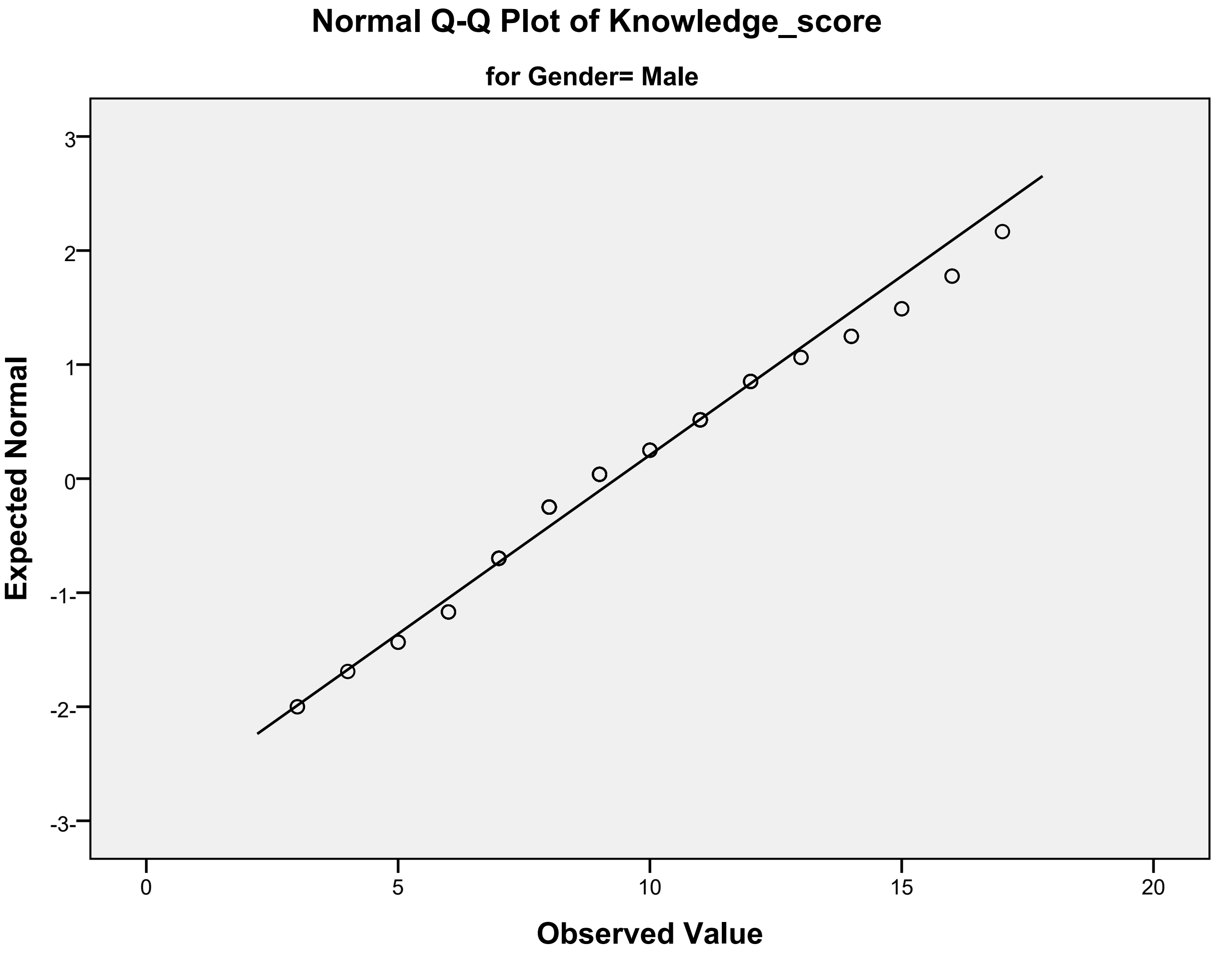

Normal Q-Q Plot of Knowledge_score
for Gender= Male
Expected Normal
3
2
1
0
-1
-2
-3
0
5
10
15
20
Observed Value

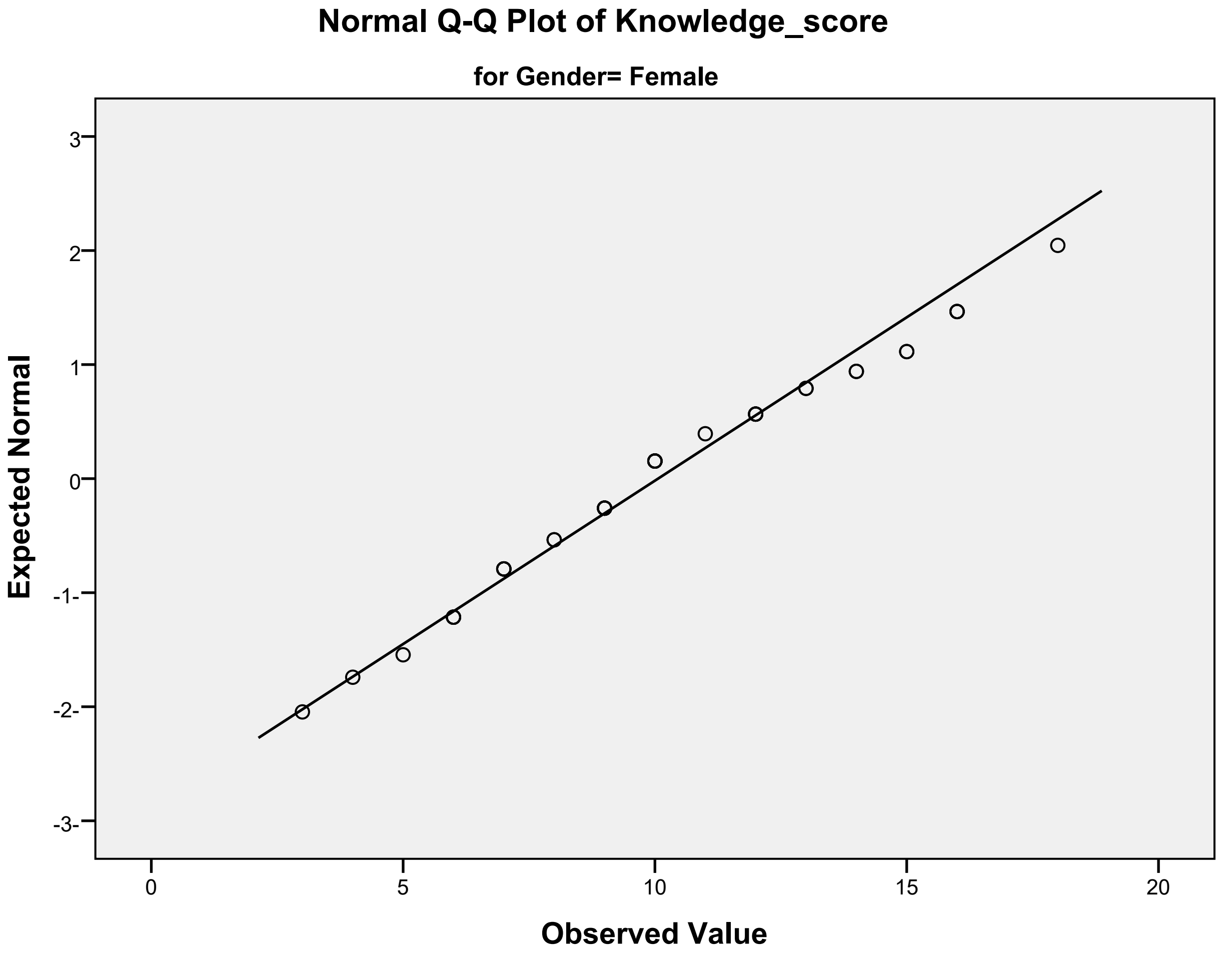

Detrended Normal Q-Q Plots

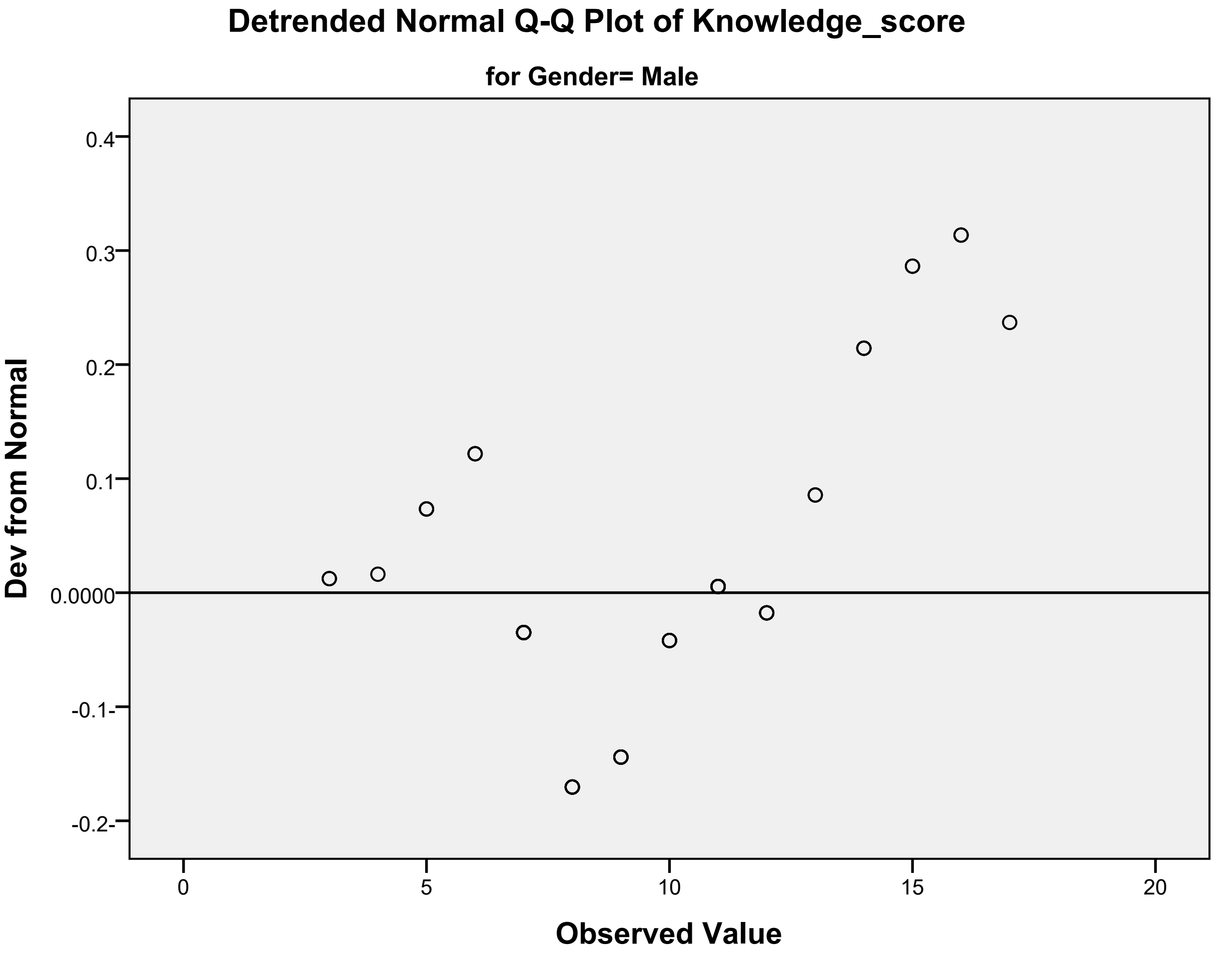

Detrended Normal Q-Q Plot of Knowledge_score
for Gender= Male
Dev from Normal
0.4
0.3
0.2
0.1
0.0000
-0.1
-0.2
0
5
10
15
20
Observed Value

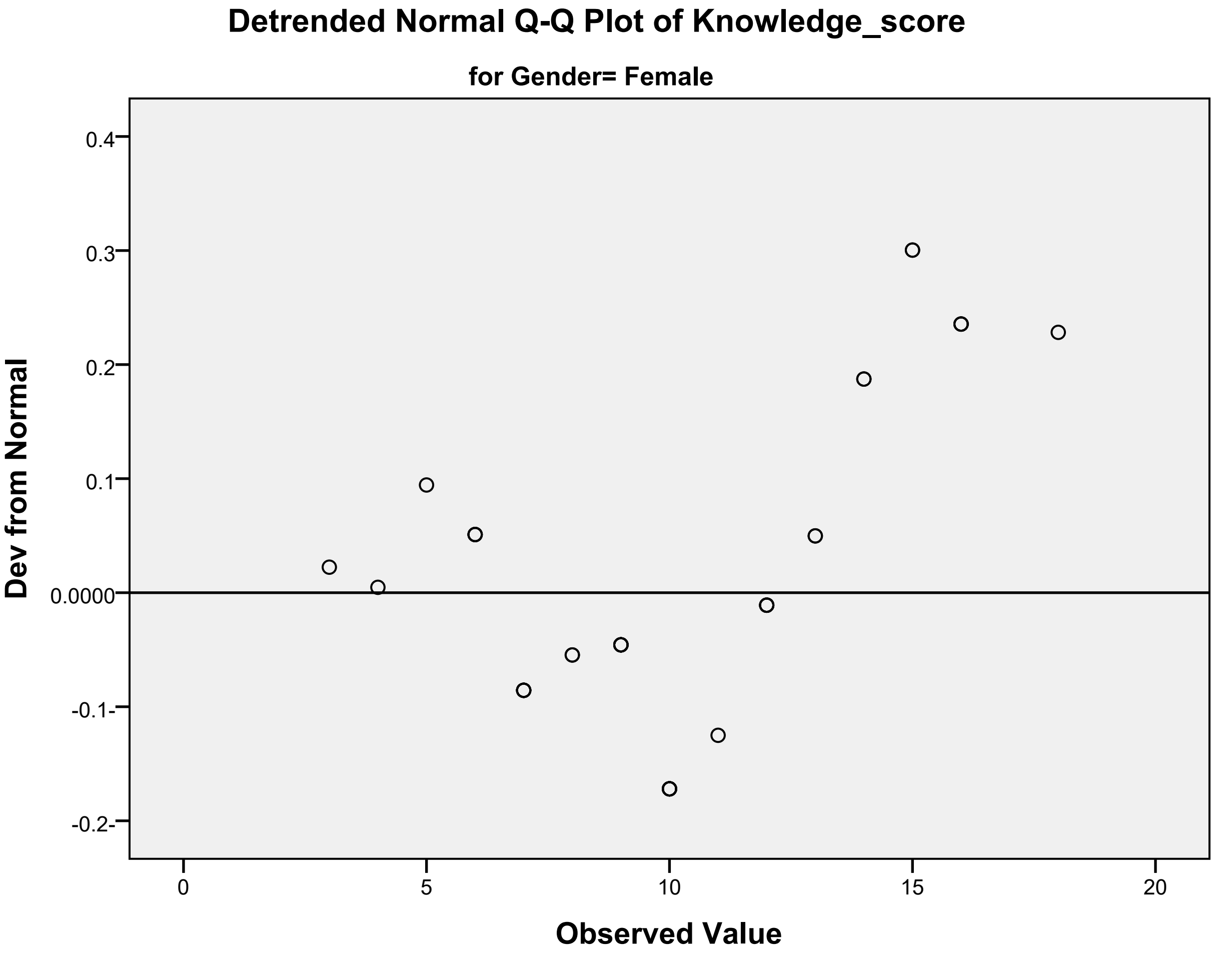

Nationality

Case Processing Summary

	Nationality	Cases					
		Valid		Missing		Total	
		N	Percent	N	Percent	N	Percent
Knowledge_score	Saudi	105	100.0%	0	0.0%	105	100.0%
	Non-Saudi	8	100.0%	0	0.0%	8	100.0%

Descriptives

	Nationality			Statistic	Std. Error
Knowledge_score	Saudi	Mean		9.75	.324
		95% Confidence Interval for Mean	Lower Bound	9.11	
			Upper Bound	10.40	
		5% Trimmed Mean		9.72	
		Median		9.00	
		Variance		11.053	
		Std. Deviation		3.325	
		Minimum		3	
		Maximum		18	
		Range		15	
		Interquartile Range		5	
		Skewness		.312	.236
		Kurtosis		-.318-	.467
	Non-Saudi	Mean		8.25	1.130
		95% Confidence Interval for Mean	Lower Bound	5.58	
			Upper Bound	10.92	
		5% Trimmed Mean		8.06	
		Median		7.50	
		Variance		10.214	
		Std. Deviation		3.196	
		Minimum		5	
		Maximum		15	
		Range		10	
		Interquartile Range		4	
		Skewness		1.484	.752
		Kurtosis		2.497	1.481

Tests of Normality

		Kolmogorov-Smirnov[a]			Shapiro-Wilk		
	Nationality	Statistic	df	Sig.	Statistic	df	Sig.
Knowledge_score	Saudi	.104	105	.007	.973	105	.031
	Non-Saudi	.167	8	.200*	.872	8	.159

*. This is a lower bound of the true significance.

a. Lilliefors Significance Correction

Knowledge_score

Histograms

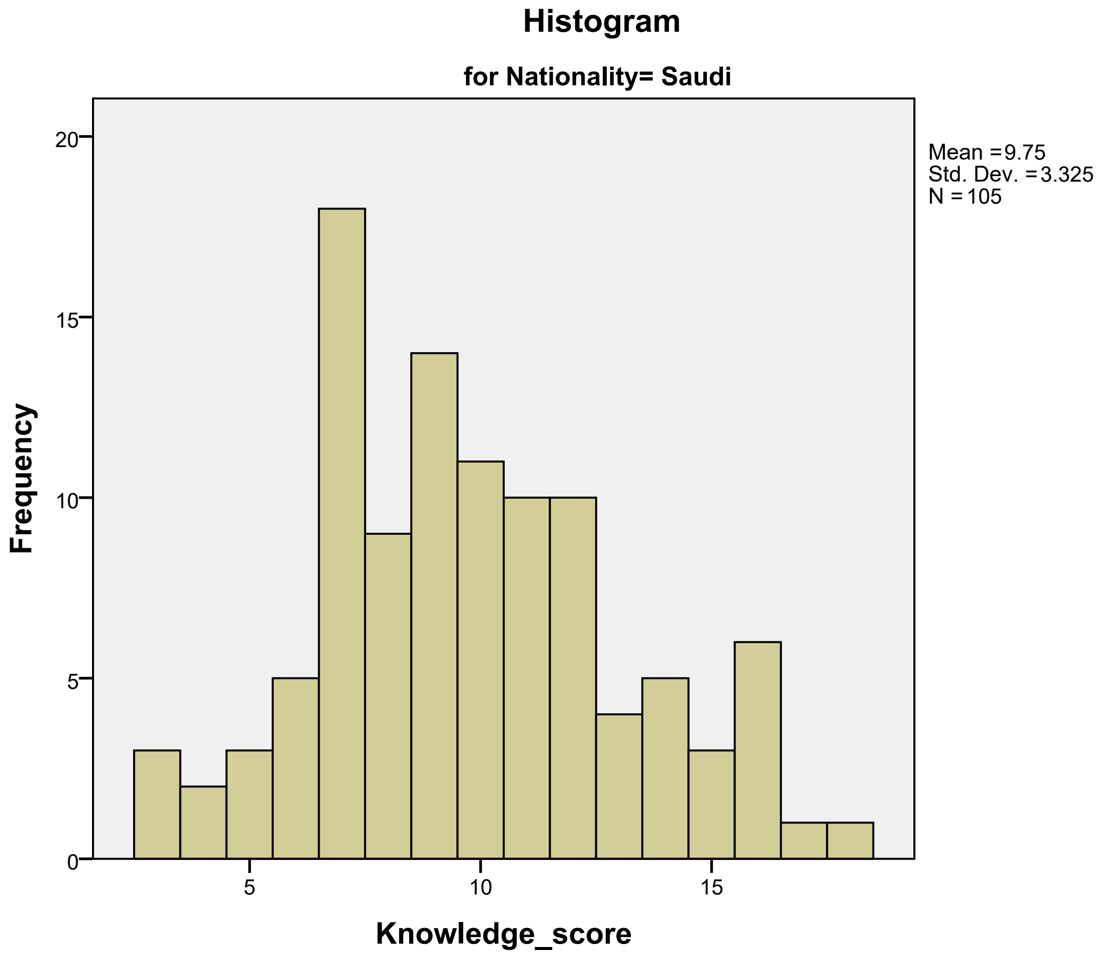
Histogram
for Nationality= Saudi
Mean =9.75
Std. Dev. =3.325
N =105
Frequency
20
15
10
5
0
5
10
15
Knowledge_score

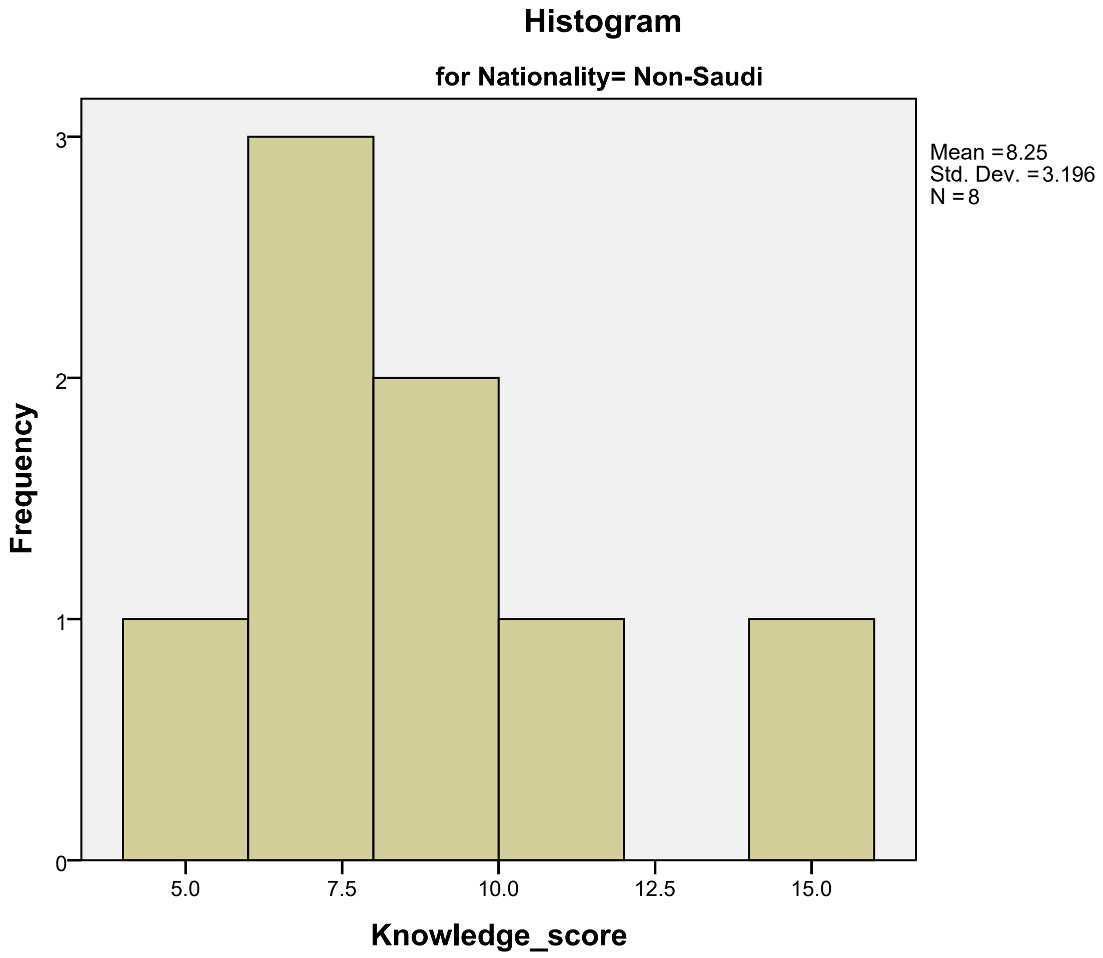

Normal Q-Q Plots

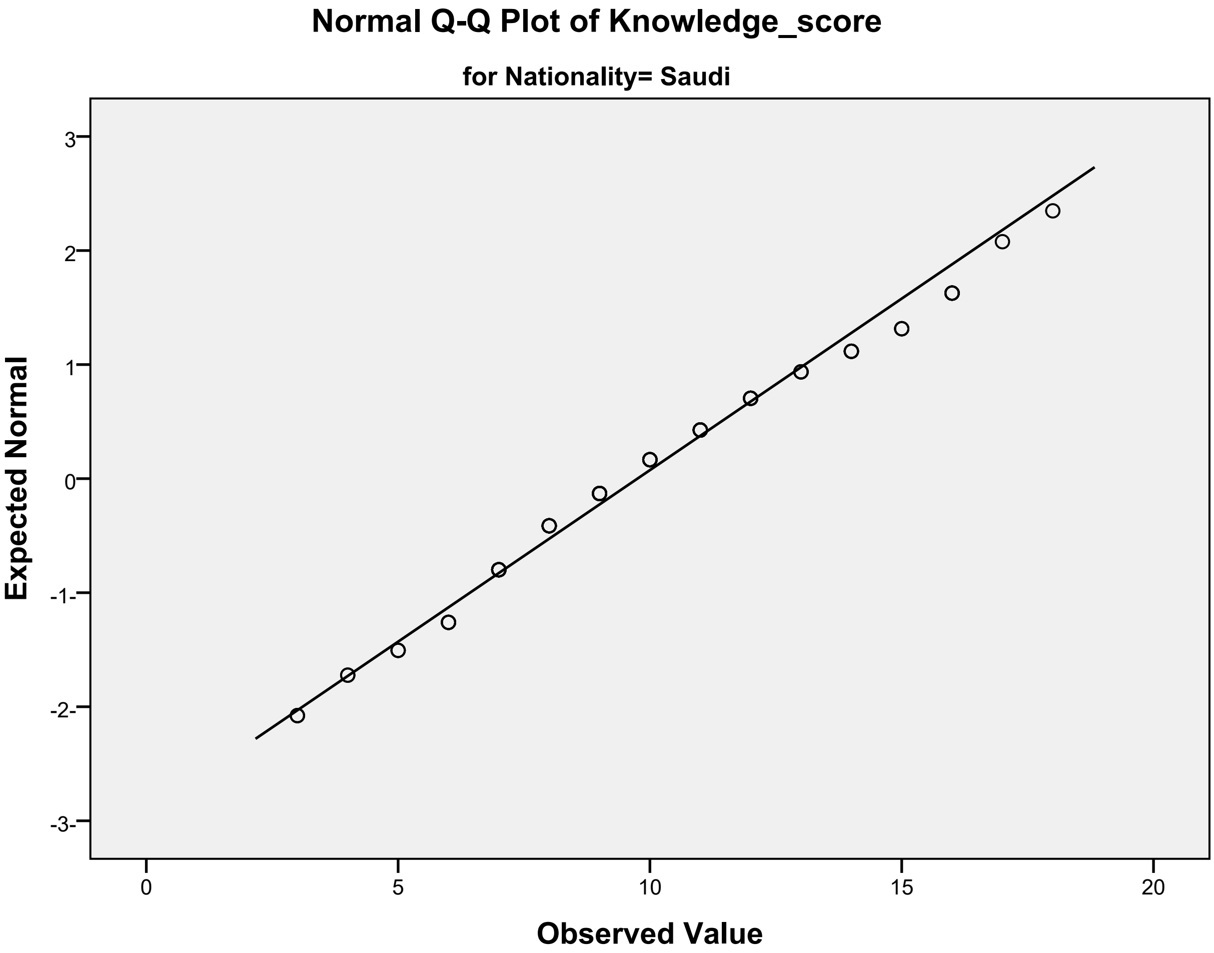
Normal Q-Q Plot of Knowledge_score
for Nationality= Saudi
Expected Normal
3
2
1
0
-1
-2
-3
0
5
10
15
20
Observed Value

Normal Q-Q Plot of Knowledge_score

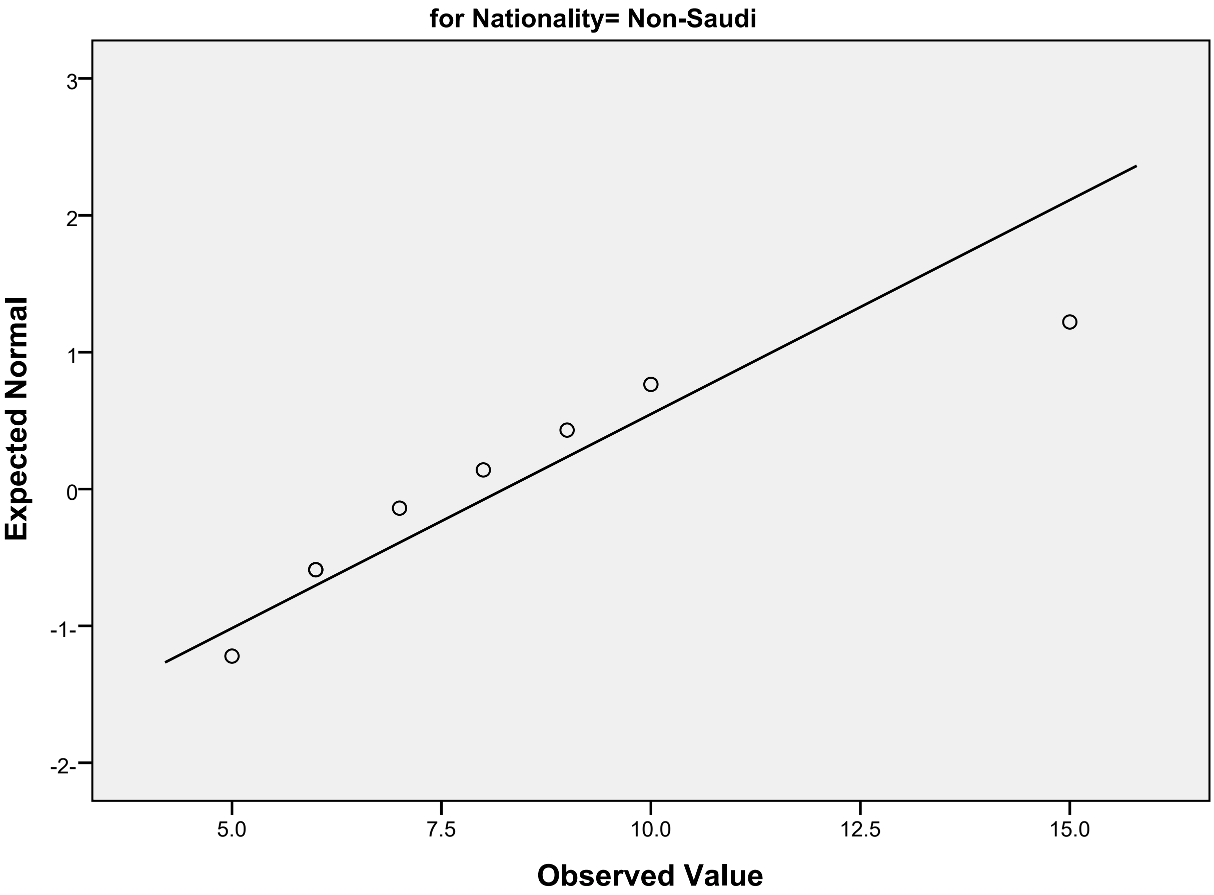

Detrended Normal Q-Q Plots

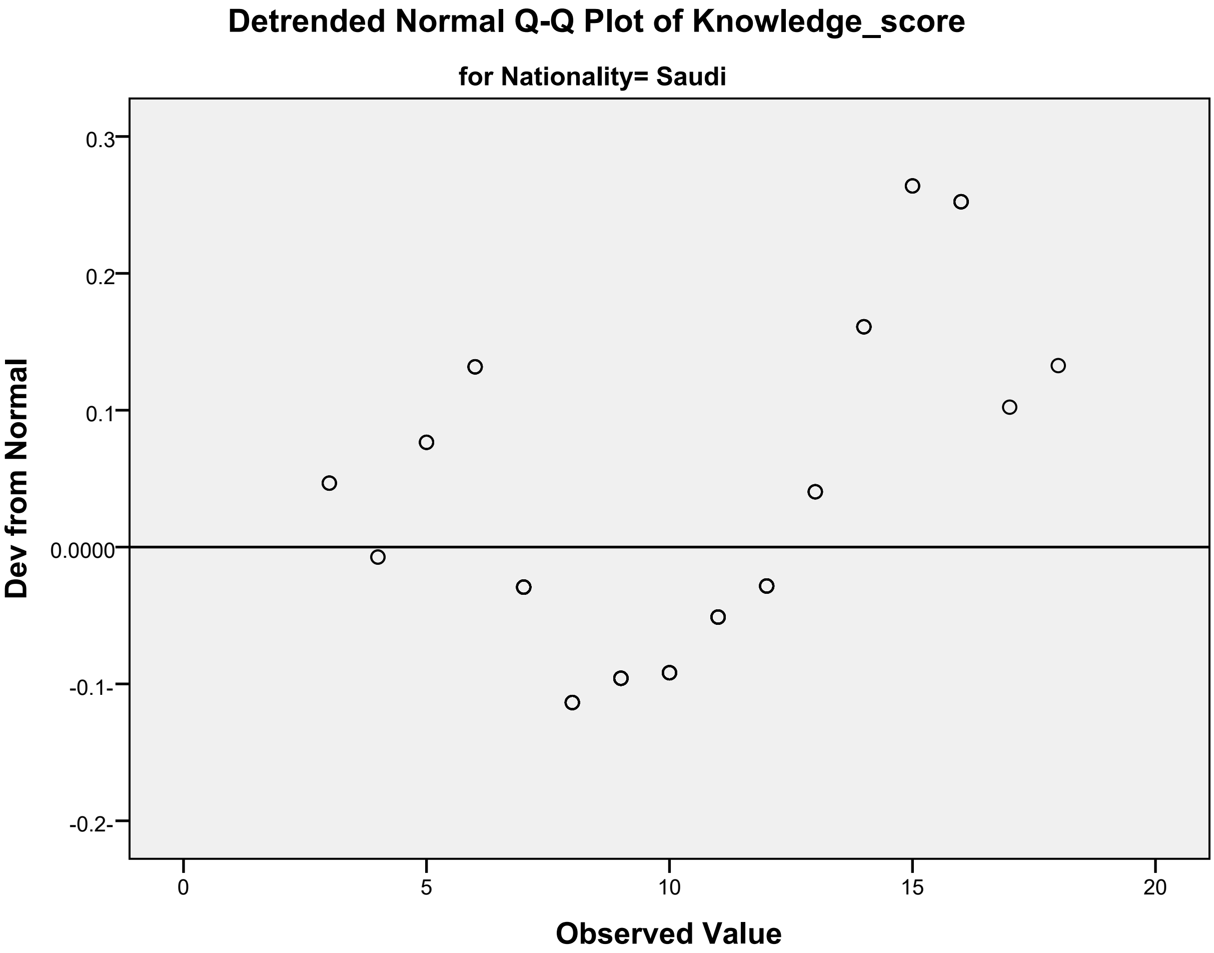
Detrended Normal Q-Q Plot of Knowledge_score
for Nationality= Saudi
Dev from Normal
0.3
0.2
0.1
0.0000
-0.1
-0.2
0
5
10
15
20
Observed Value

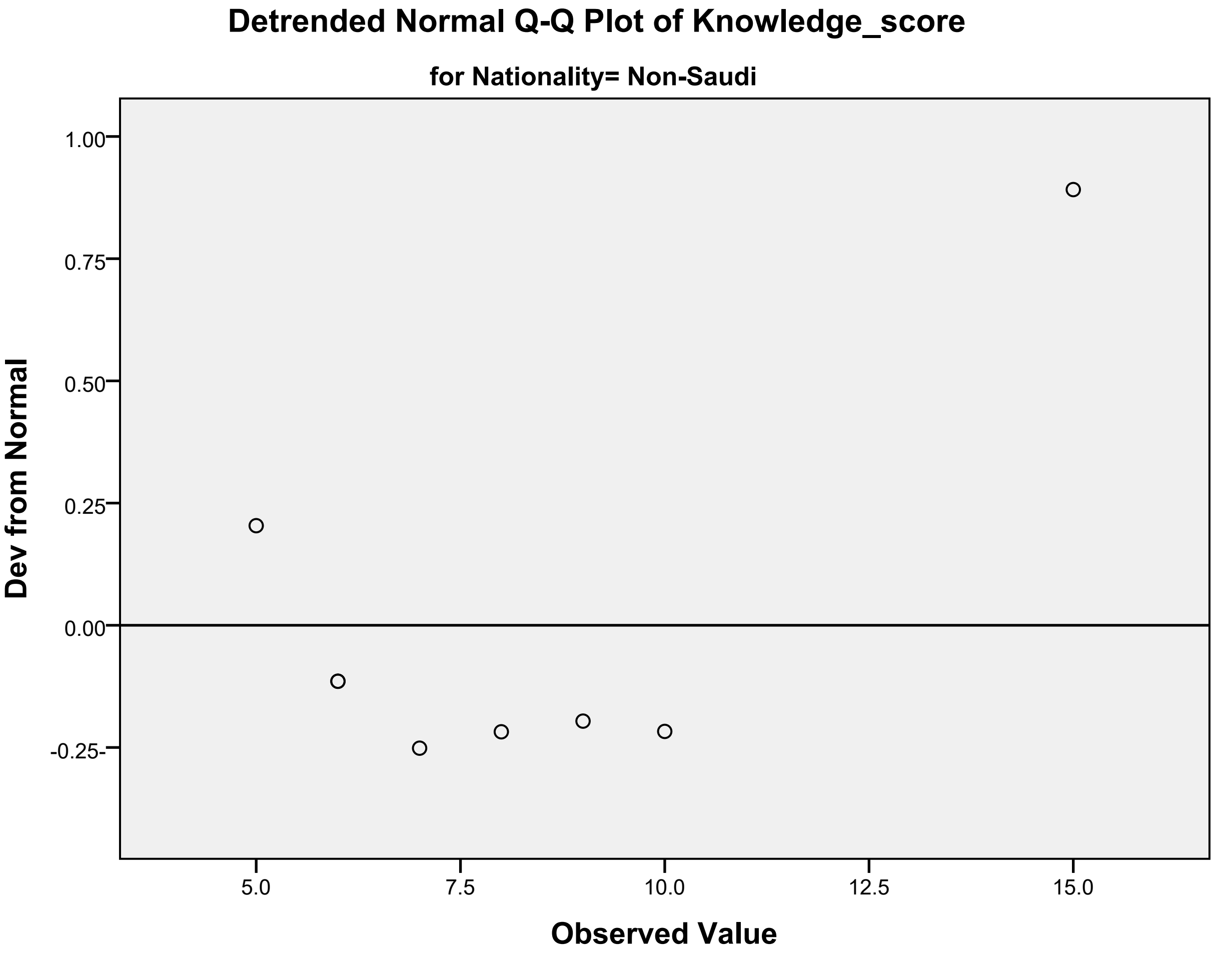

hospital_cat2

Case Processing Summary

		Cases					
		Valid		Missing		Total	
	hospital_cat2	N	Percent	N	Percent	N	Percent
Knowledge_score	Academic & training centers	28	100.0%	0	0.0%	28	100.0%
	Other centers	85	100.0%	0	0.0%	85	100.0%

Descriptives

	hospital_cat2			Statistic	Std. Error
Knowledge_score	Academic & training centers	Mean		10.07	.619
		95% Confidence Interval for Mean	Lower Bound	8.80	
			Upper Bound	11.34	
		5% Trimmed Mean		10.06	
		Median		10.50	
		Variance		10.735	
		Std. Deviation		3.276	
		Minimum		3	
		Maximum		17	
		Range		14	
		Interquartile Range		5	
		Skewness		.095	.441
		Kurtosis		-.024-	.858
	Other centers	Mean		9.51	.363
		95% Confidence Interval for Mean	Lower Bound	8.78	
			Upper Bound	10.23	
		5% Trimmed Mean		9.45	
		Median		9.00	
		Variance		11.205	
		Std. Deviation		3.347	
		Minimum		3	
		Maximum		18	
		Range		15	
		Interquartile Range		5	
		Skewness		.459	.261
		Kurtosis		-.320-	.517

Tests of Normality

		Kolmogorov-Smirnov[a]			Shapiro-Wilk		
	hospital_cat2	Statistic	df	Sig.	Statistic	df	Sig.
Knowledge_score	Academic & training centers	.112	28	.200*	.982	28	.895
	Other centers	.125	85	.002	.963	85	.014

*. This is a lower bound of the true significance.

a. Lilliefors Significance Correction

Knowledge_score

Histograms

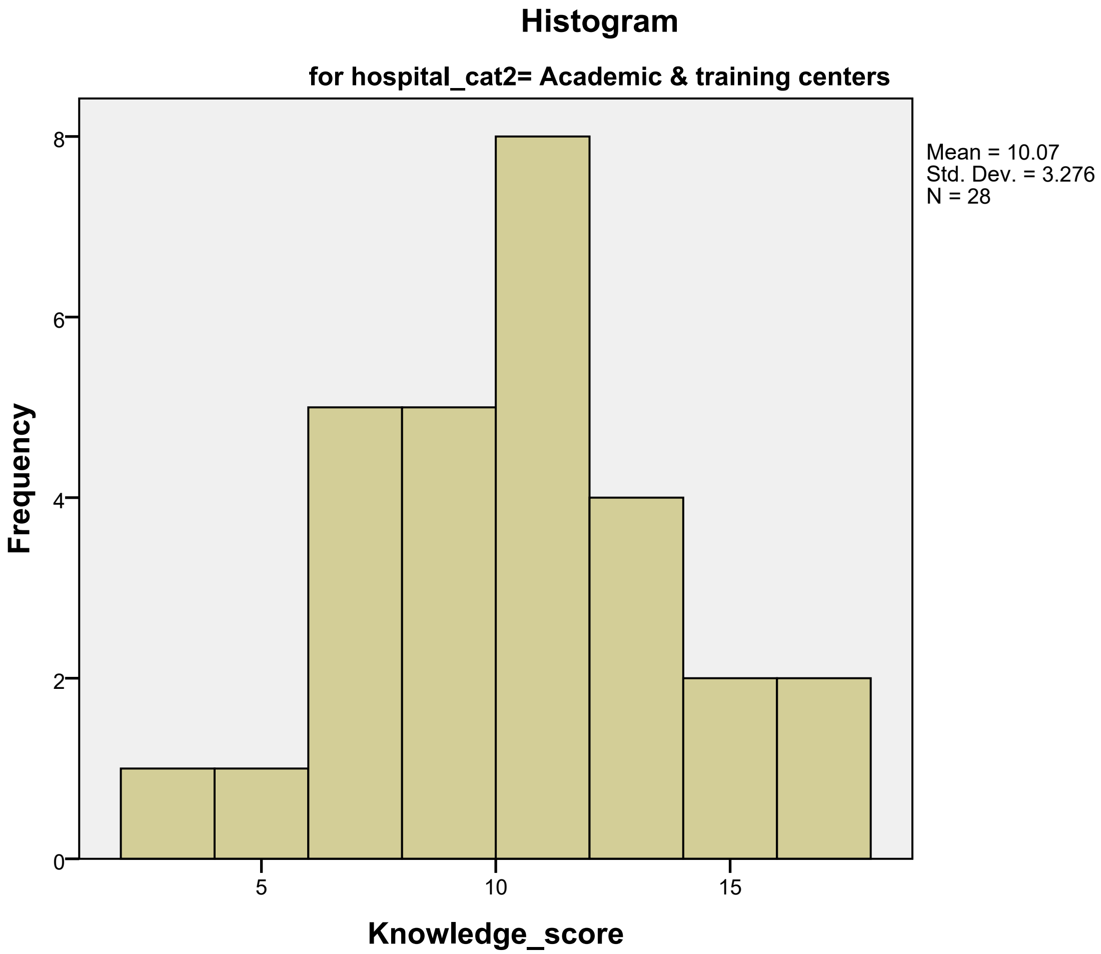
Histogram
for hospital_cat2= Academic & training centers
Mean = 10.07
Std. Dev. = 3.276
N = 28
Frequency
Knowledge_score
0
2
4
6
8
5
10
15

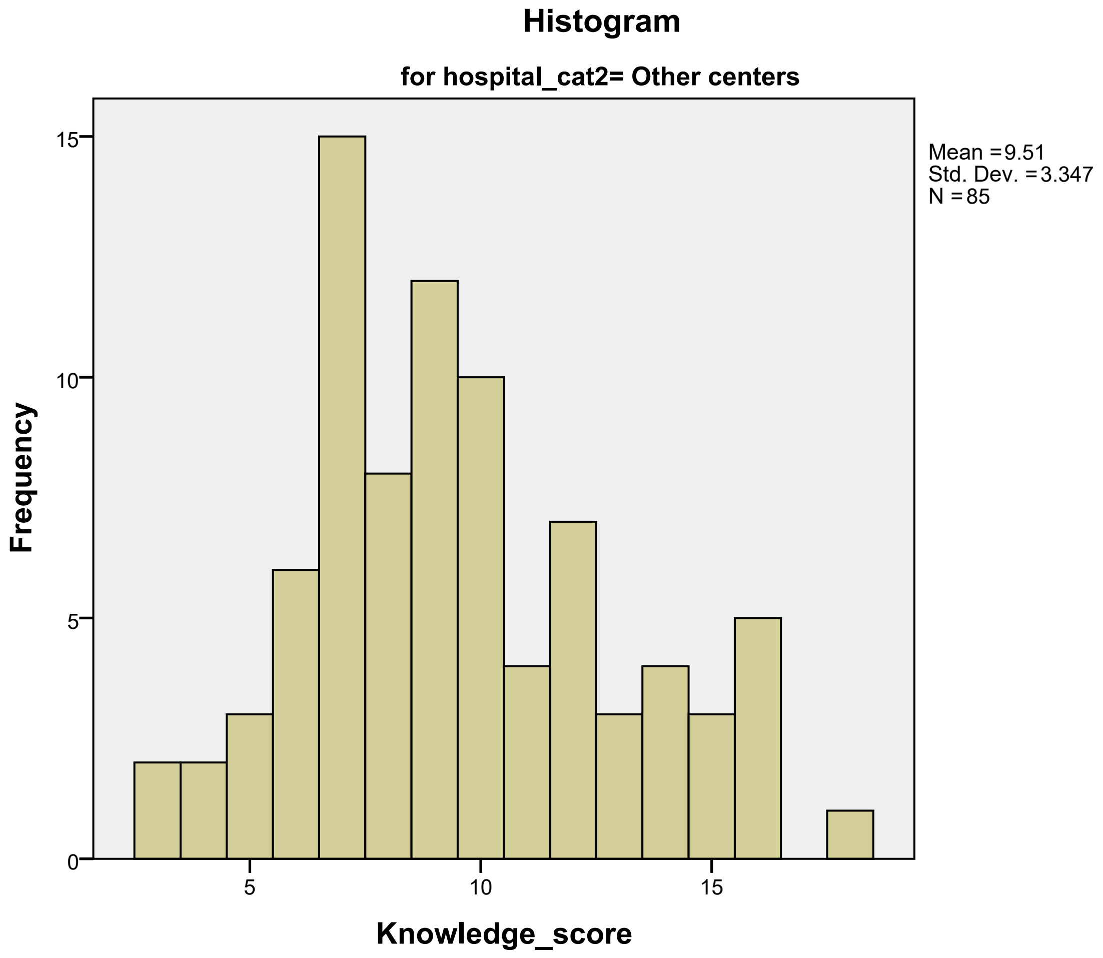

Normal Q-Q Plots

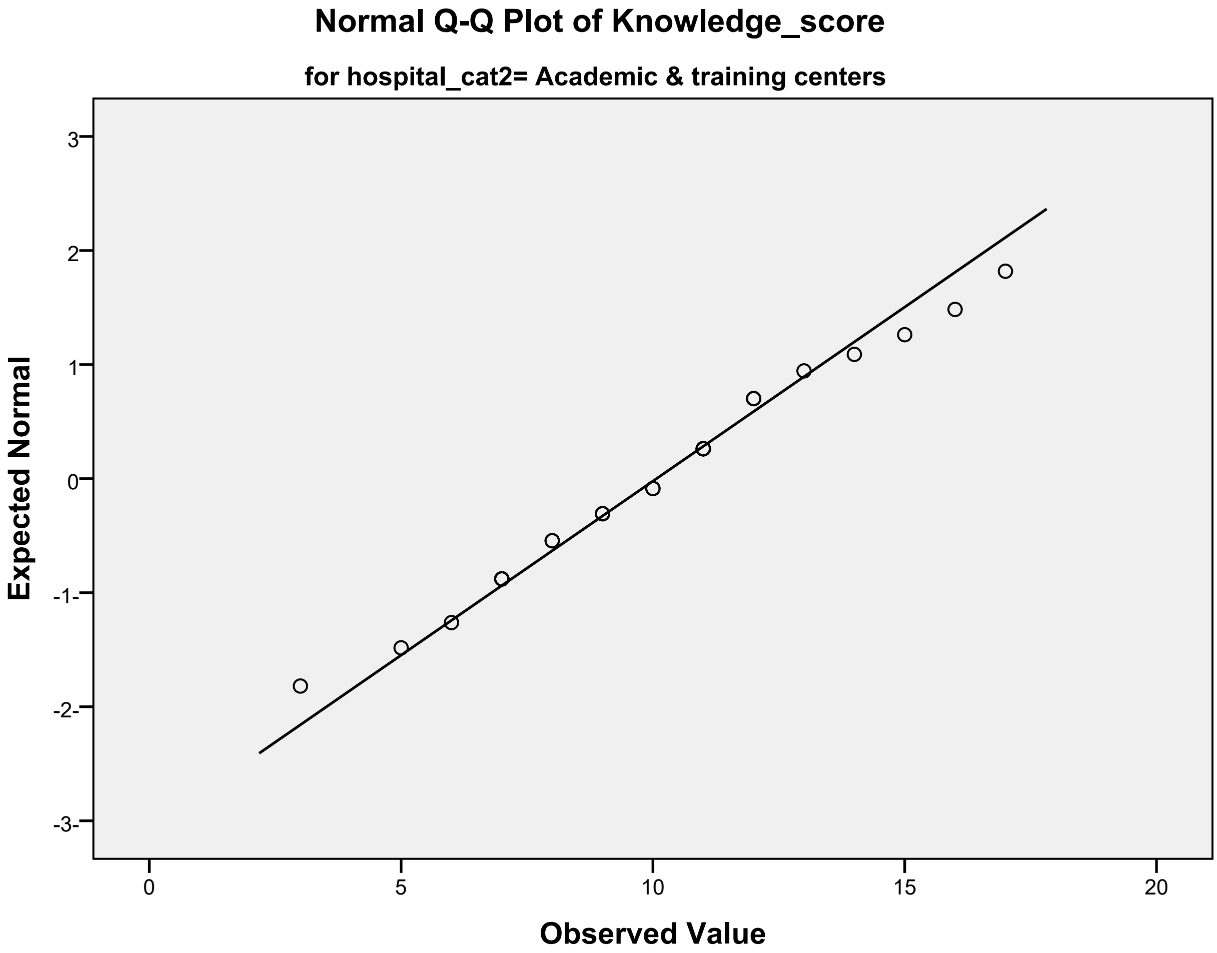
Normal Q-Q Plot of Knowledge_score
for hospital_cat2= Academic & training centers
Expected Normal
3
2
1
0
-1
-2
-3
0
5
10
15
20
Observed Value

Normal Q-Q Plot of Knowledge_score

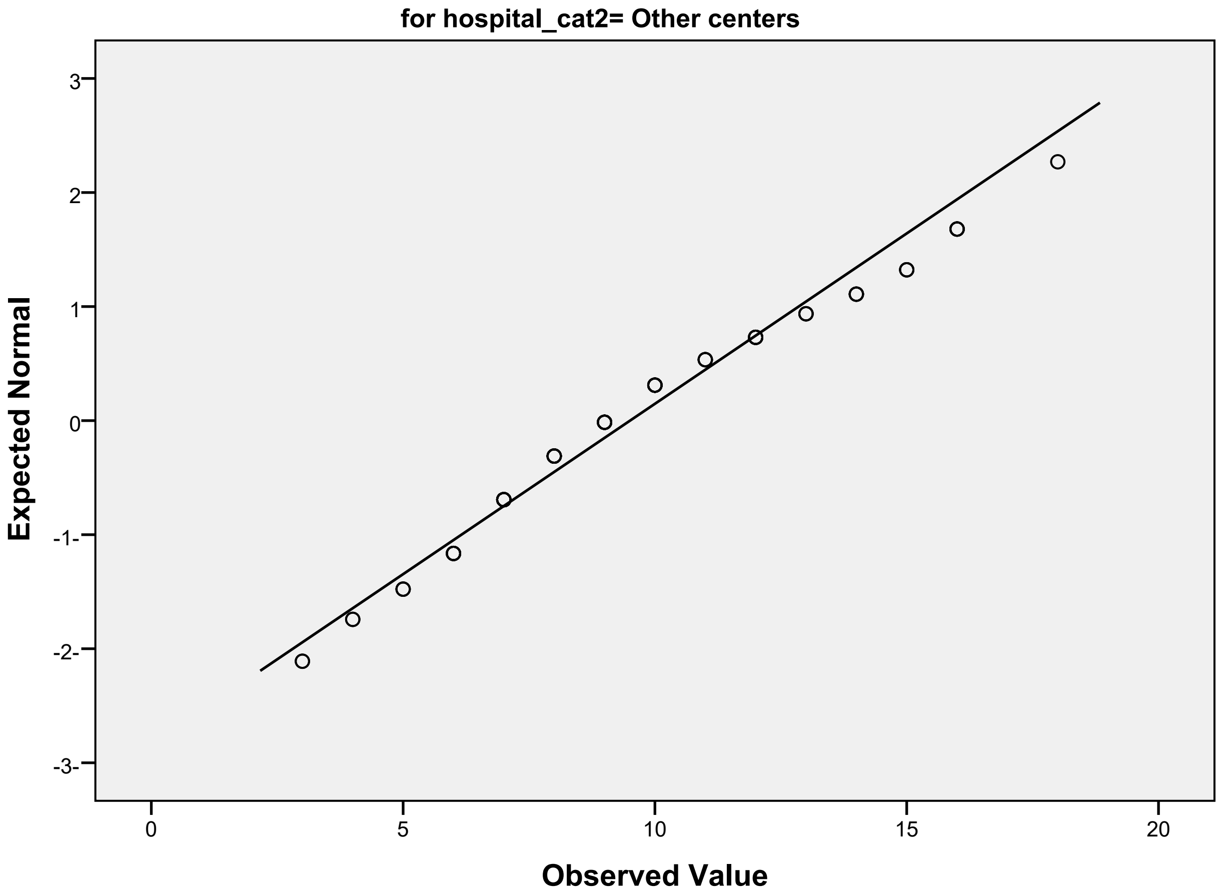

Detrended Normal Q-Q Plots

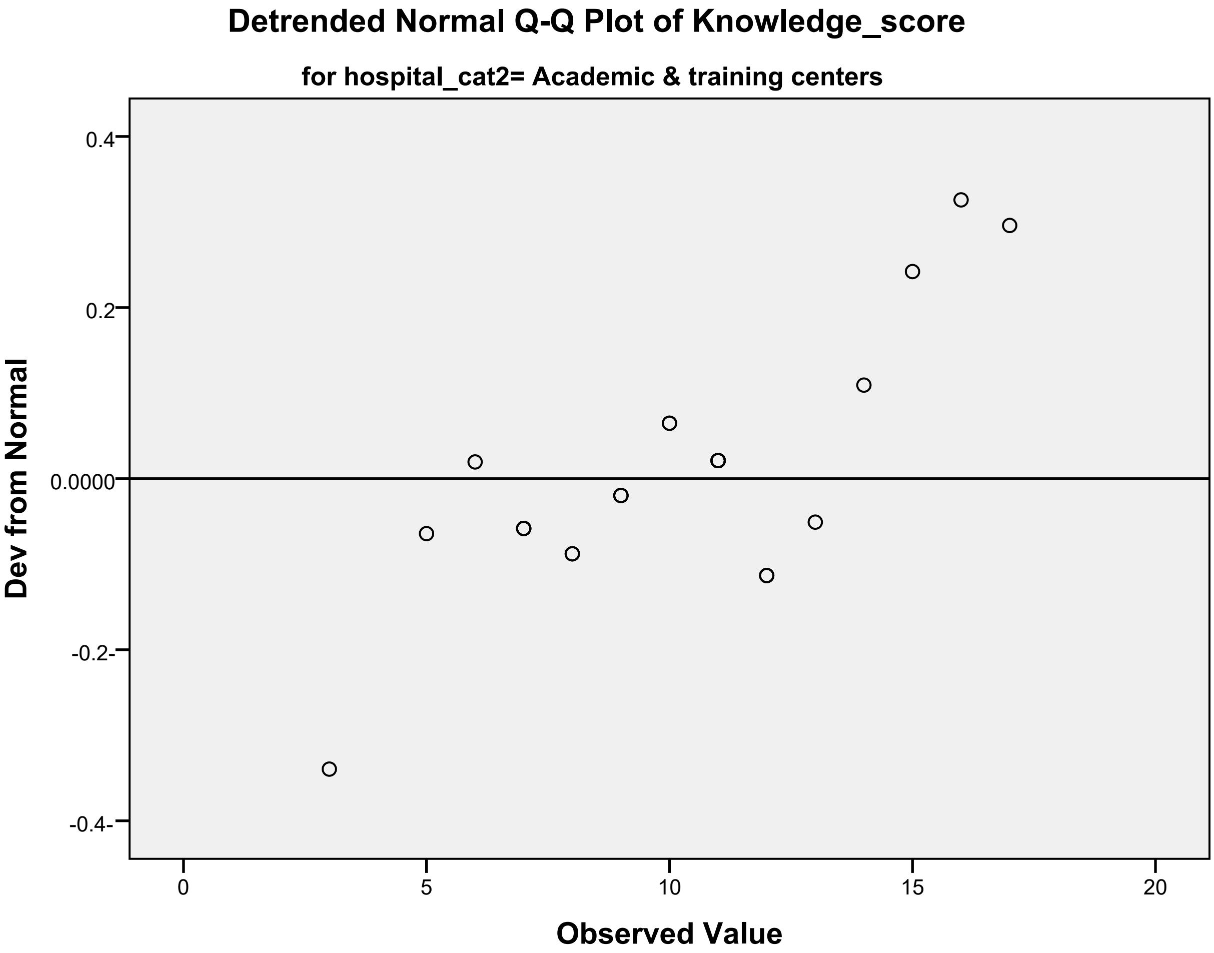
Detrended Normal Q-Q Plot of Knowledge_score
for hospital_cat2= Academic & training centers
0.4
0.2
0.0000
-0.2
-0.4
Dev from Normal
0
5
10
15
20
Observed Value

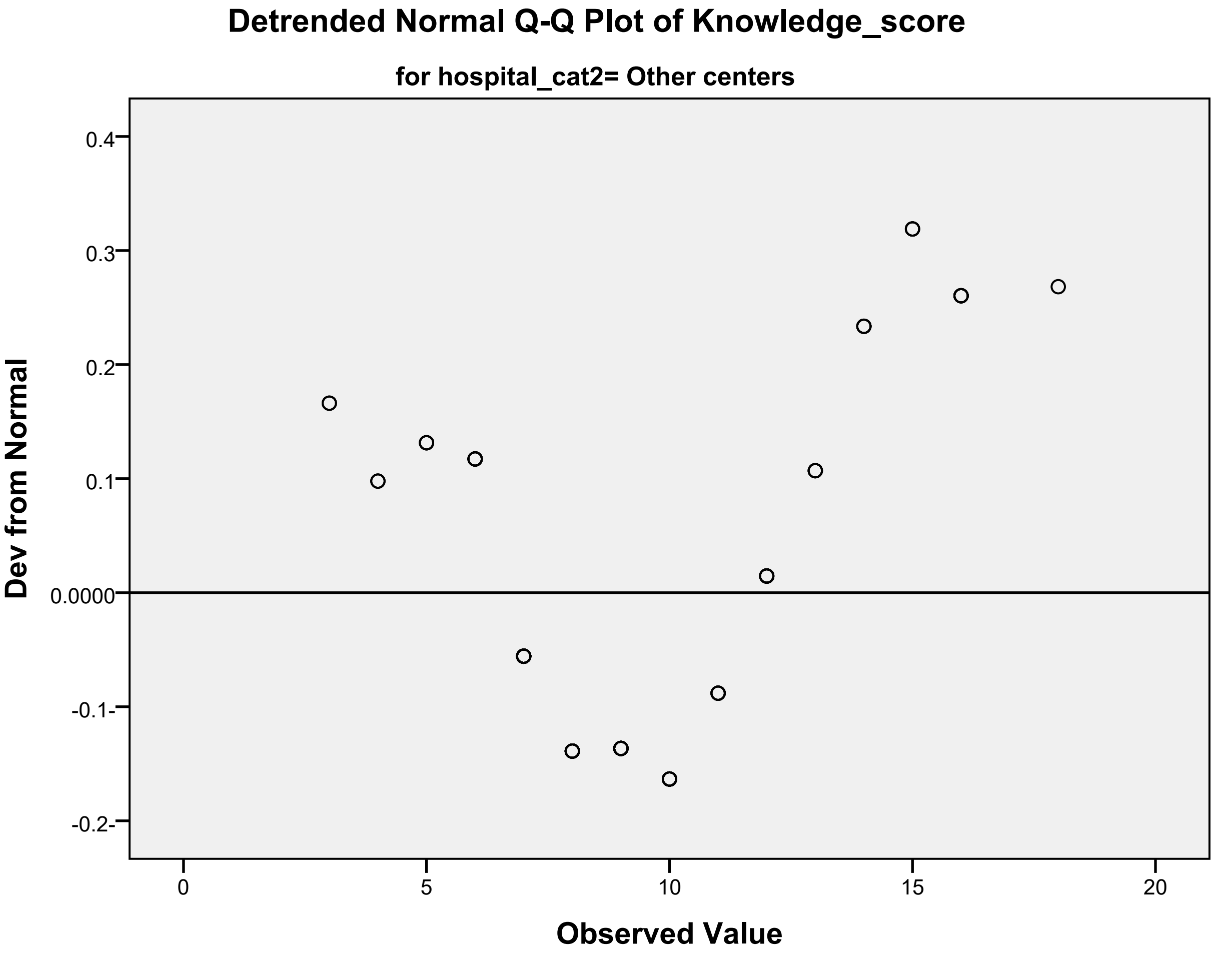


```
EXAMINE VARIABLES=Attitude_score BY Gender Nationality hospital_cat2
 /PLOT HISTOGRAM NPPLOT
 /STATISTICS DESCRIPTIVES
 /CINTERVAL 95
 /MISSING LISTWISE
 /NOTOTAL.
```

Explore

Notes

Output Created		19-SEP-2026 23:51:36
Comments		
Input	Data	C:\Users\Mosaab Lap\Desktop\SPSS Files\SPSS 419\Data 419.sav
	Active Dataset	DataSet6
	Filter	<none>
	Weight	<none>
	Split File	<none>
	N of Rows in Working Data File	113
Missing Value Handling	Definition of Missing	User-defined missing values for dependent variables are treated as missing.
	Cases Used	Statistics are based on cases with no missing values for any dependent variable or factor used.
Syntax		EXAMINE VARIABLES=Attitude_score BY Gender Nationality hospital_cat2<br>/PLOT HISTOGRAM NPPLOT<br>/STATISTICS DESCRIPTIVES<br>/CINTERVAL 95<br>/MISSING LISTWISE<br>/NOTOTAL.
Resources	Processor Time	00:00:01.28
	Elapsed Time	00:00:01.16

Gender

Case Processing Summary

		Cases					
		Valid		Missing		Total	
	Gender	N	Percent	N	Percent	N	Percent
Attitude_score	Male	65	100.0%	0	0.0%	65	100.0%
	Female	48	100.0%	0	0.0%	48	100.0%

Descriptives

	Gender			Statistic	Std. Error
Attitude_score	Male	Mean		4.11	.158
		95% Confidence Interval for Mean	Lower Bound	3.79	
			Upper Bound	4.42	
		5% Trimmed Mean		4.14	
		Median		4.00	
		Variance		1.629	
		Std. Deviation		1.276	
		Minimum		1	
		Maximum		6	
		Range		5	
		Interquartile Range		2	
		Skewness		-.021-	.297
		Kurtosis		-.798-	.586
	Female	Mean		3.98	.170
		95% Confidence Interval for Mean	Lower Bound	3.64	
			Upper Bound	4.32	
		5% Trimmed Mean		3.98	
		Median		4.00	
		Variance		1.383	
		Std. Deviation		1.176	
		Minimum		2	
		Maximum		6	
		Range		4	
		Interquartile Range		2	
		Skewness		.042	.343
		Kurtosis		-.861-	.674

Tests of Normality

		Kolmogorov-Smirnov[a]			Shapiro-Wilk		
	Gender	Statistic	df	Sig.	Statistic	df	Sig.
Attitude_score	Male	.177	65	.000	.913	65	.000
	Female	.173	48	.001	.916	48	.002

a. Lilliefors Significance Correction

Attitude_score

Histograms

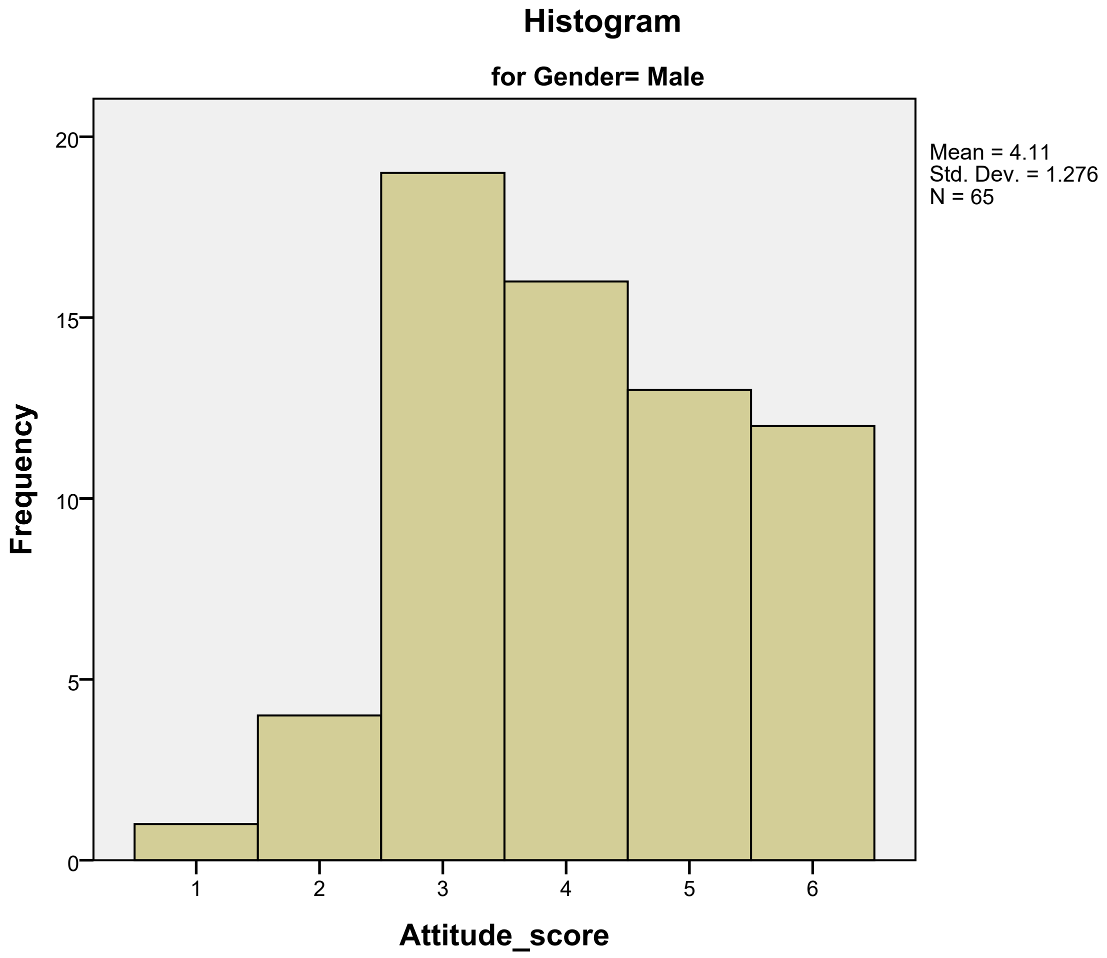
Histogram
for Gender= Male
Mean = 4.11
Std. Dev. = 1.276
N = 65
Frequency
20
15
10
5
0
1
2
3
4
5
6
Attitude_score

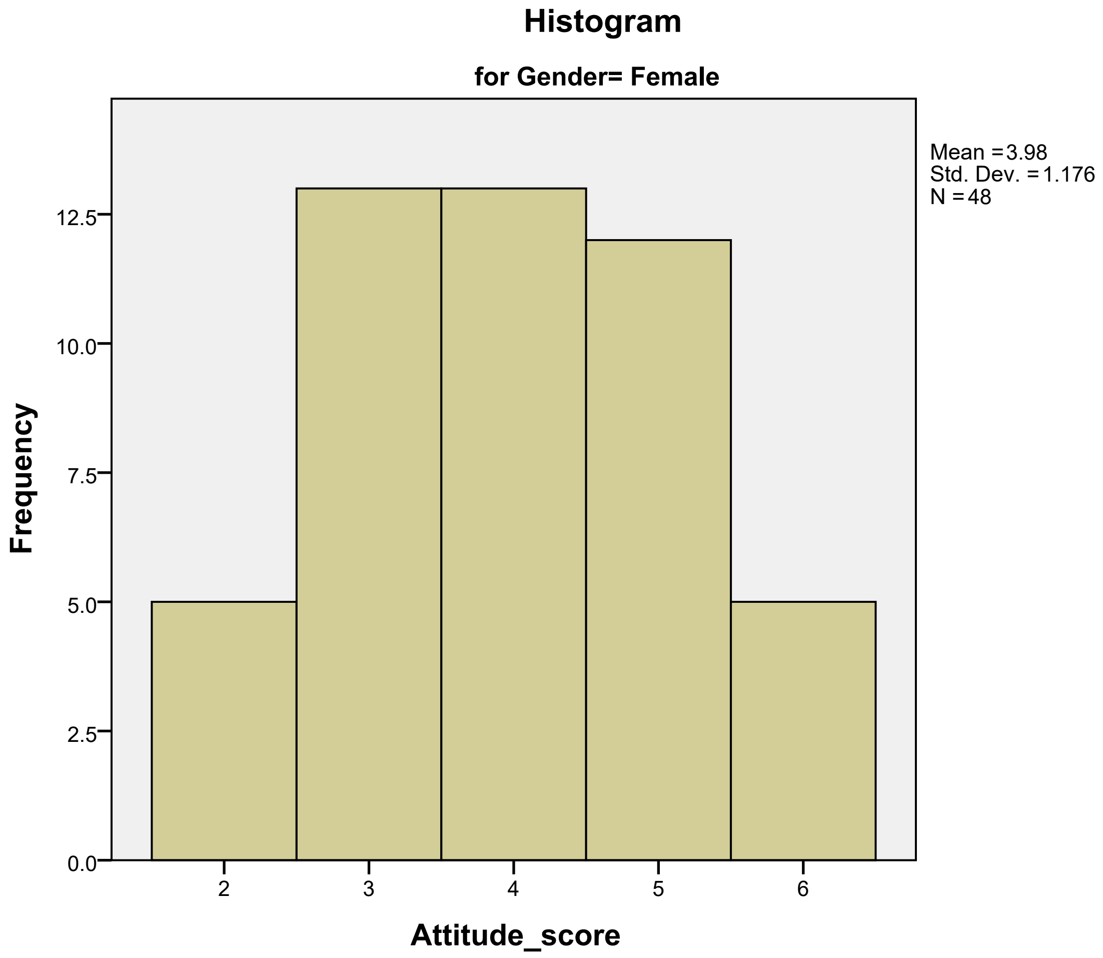

Normal Q-Q Plots

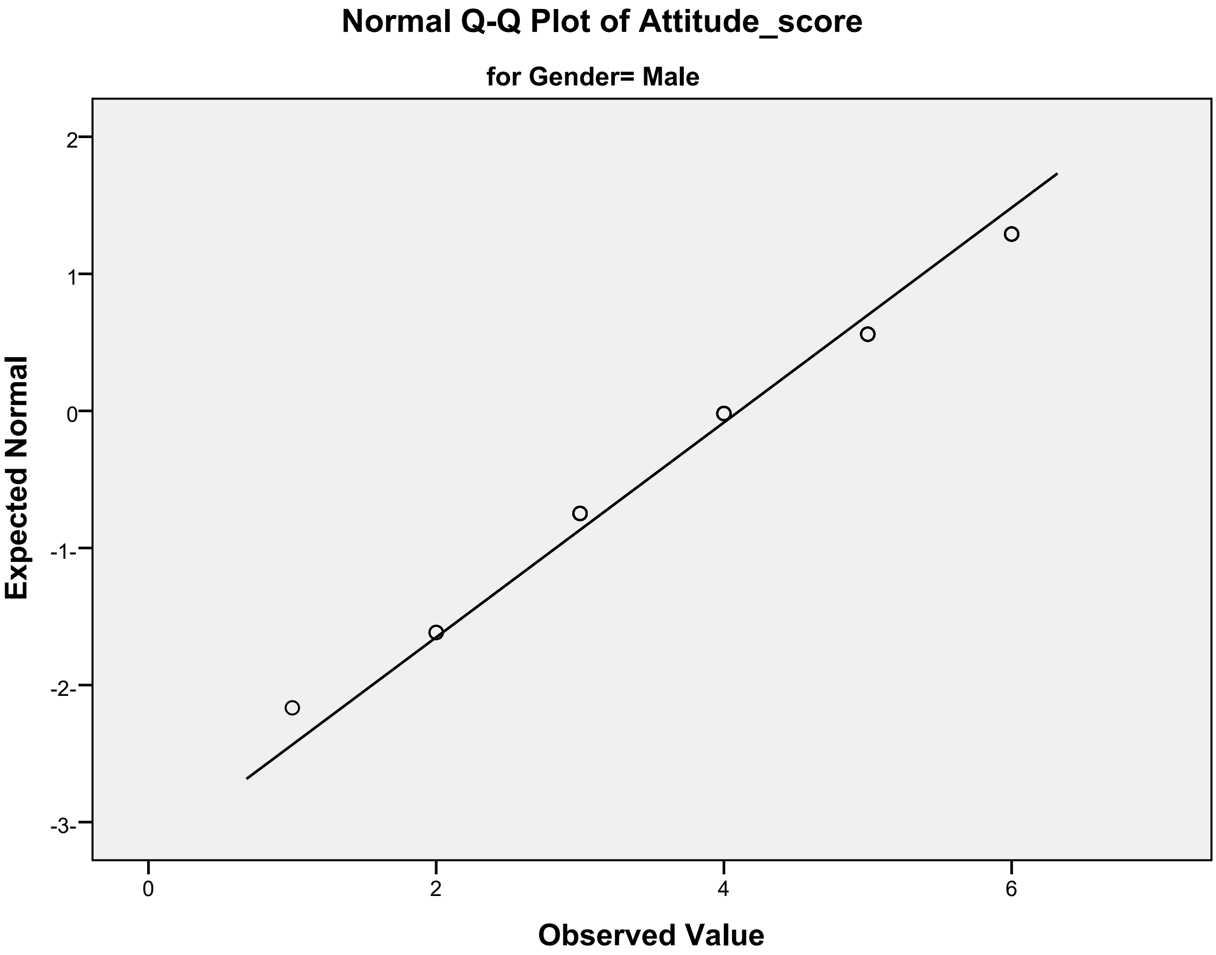

Normal Q-Q Plot of Attitude_score
for Gender= Male
Expected Normal
2
1
0
-1
-2
-3
0
2
4
6
Observed Value

Normal Q-Q Plot of Attitude_score

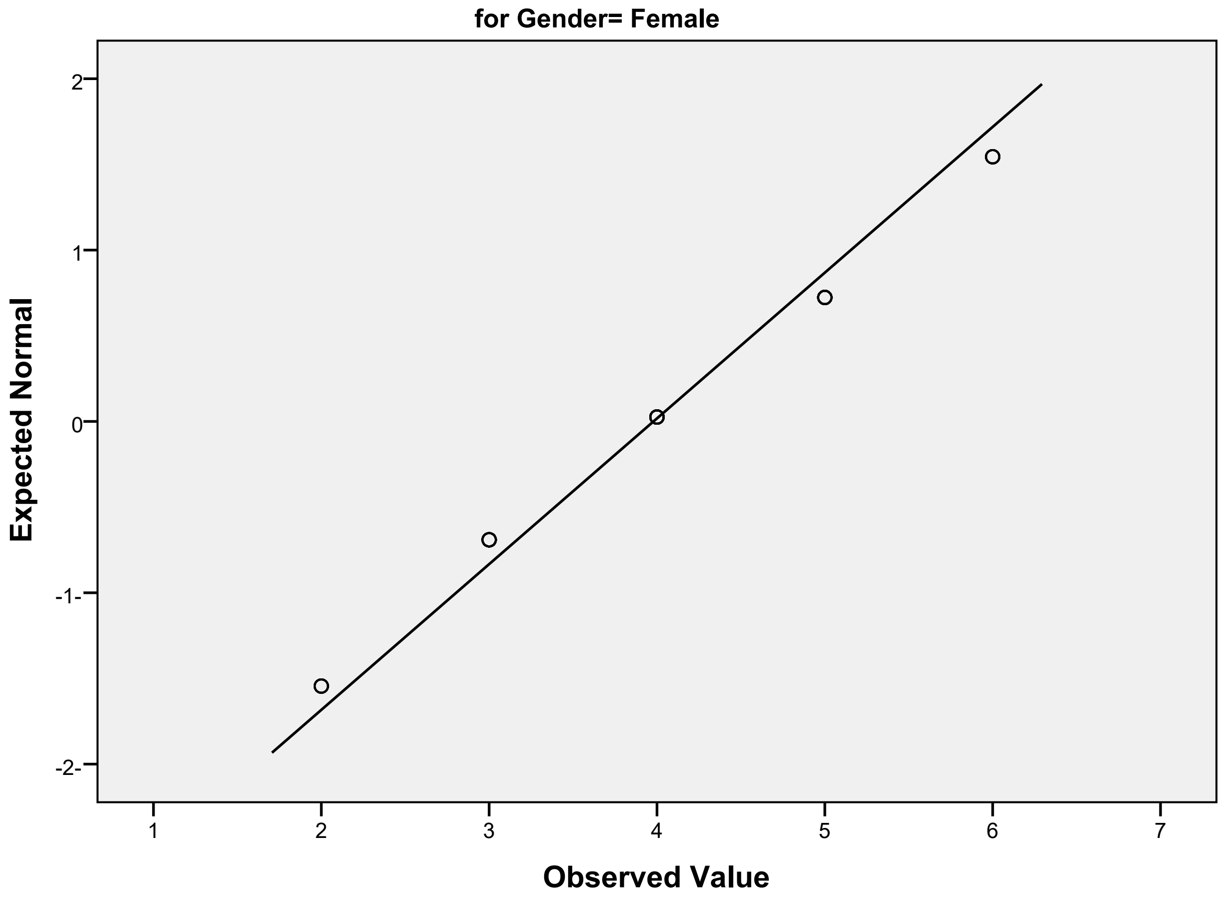

Detrended Normal Q-Q Plots

Detrended Normal Q-Q Plot of Attitude_score

for Gender= Male

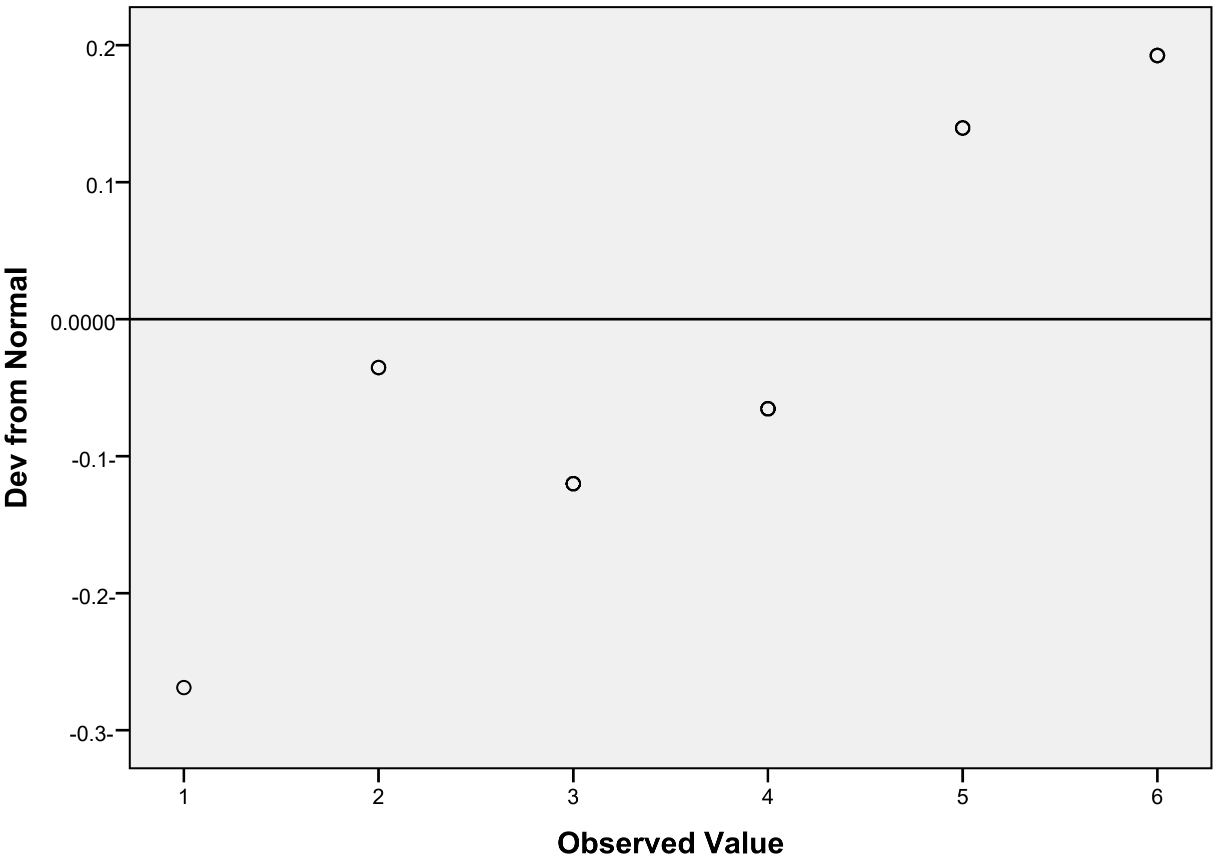

Detrended Normal Q-Q Plot of Attitude_score

for Gender= Female

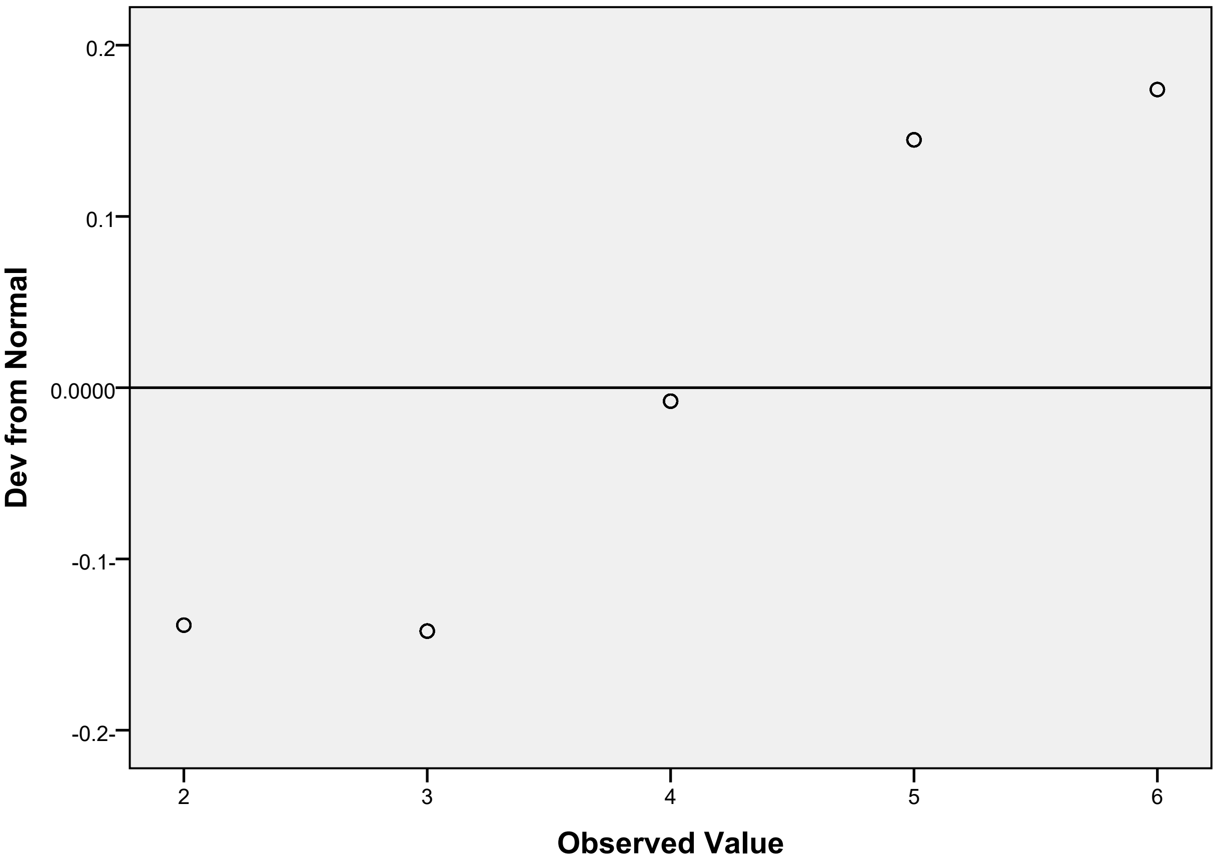

Nationality

Case Processing Summary

		Cases					
		Valid		Missing		Total	
	Nationality	N	Percent	N	Percent	N	Percent
Attitude_score	Saudi	105	100.0%	0	0.0%	105	100.0%
	Non-Saudi	8	100.0%	0	0.0%	8	100.0%

Descriptives

	Nationality			Statistic	Std. Error
Attitude_score	Saudi	Mean		4.02	.120
		95% Confidence Interval for Mean	Lower Bound	3.78	
			Upper Bound	4.26	
		5% Trimmed Mean		4.03	
		Median		4.00	
		Variance		1.519	
		Std. Deviation		1.232	
		Minimum		1	
		Maximum		6	
		Range		5	
		Interquartile Range		2	
		Skewness		.026	.236
		Kurtosis		-.804-	.467
	Non-Saudi	Mean		4.50	.423
		95% Confidence Interval for Mean	Lower Bound	3.50	
			Upper Bound	5.50	
		5% Trimmed Mean		4.50	
		Median		4.50	
		Variance		1.429	
		Std. Deviation		1.195	
		Minimum		3	
		Maximum		6	
		Range		3	
		Interquartile Range		3	
		Skewness		.000	.752
		Kurtosis		-1.456-	1.481

Tests of Normality

		Kolmogorov-Smirnov[a]			Shapiro-Wilk		
	Nationality	Statistic	df	Sig.	Statistic	df	Sig.
Attitude_score	Saudi	.177	105	.000	.921	105	.000
	Non-Saudi	.162	8	.200*	.897	8	.274

*. This is a lower bound of the true significance.

a. Lilliefors Significance Correction

Attitude_score

Histograms

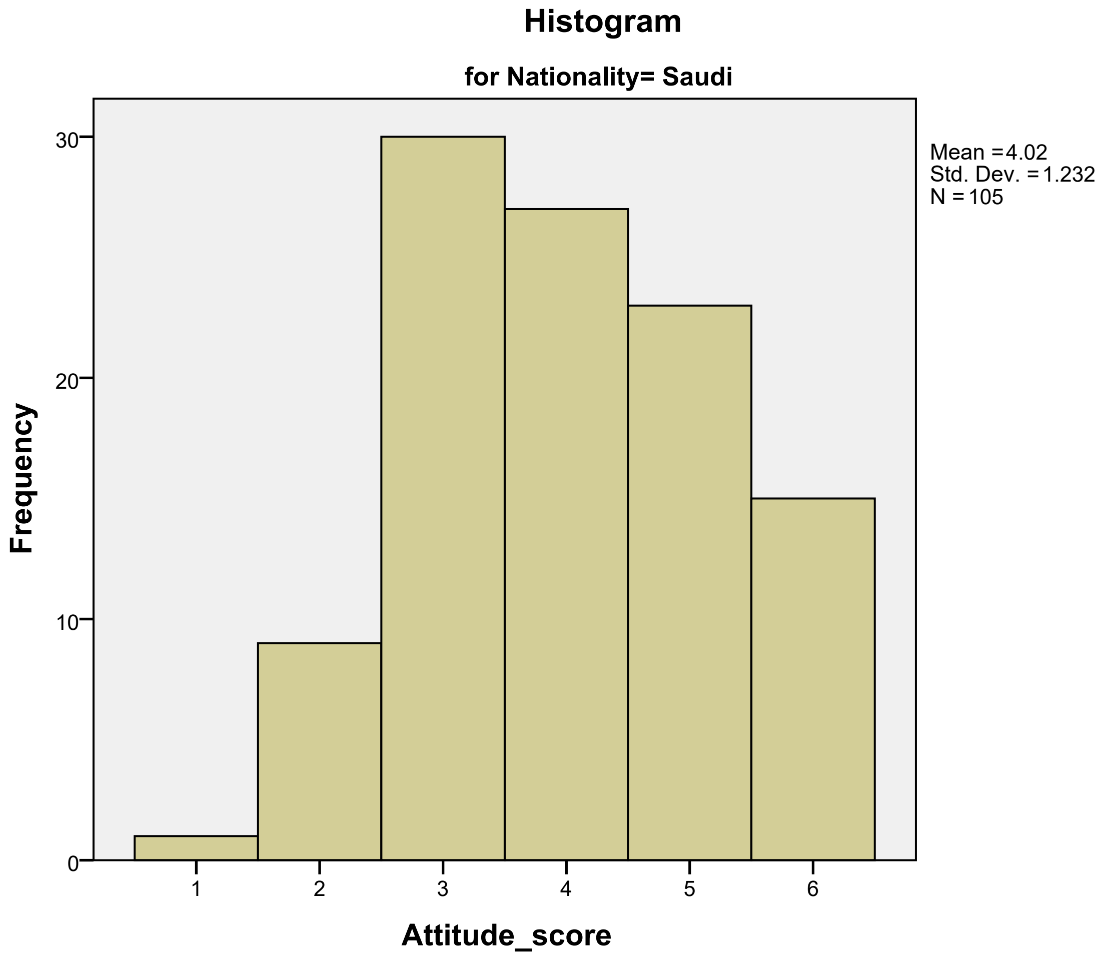
Histogram
for Nationality= Saudi
Mean =4.02
Std. Dev. =1.232
N =105
Frequency
30
20
10
0
1
2
3
4
5
6
Attitude_score

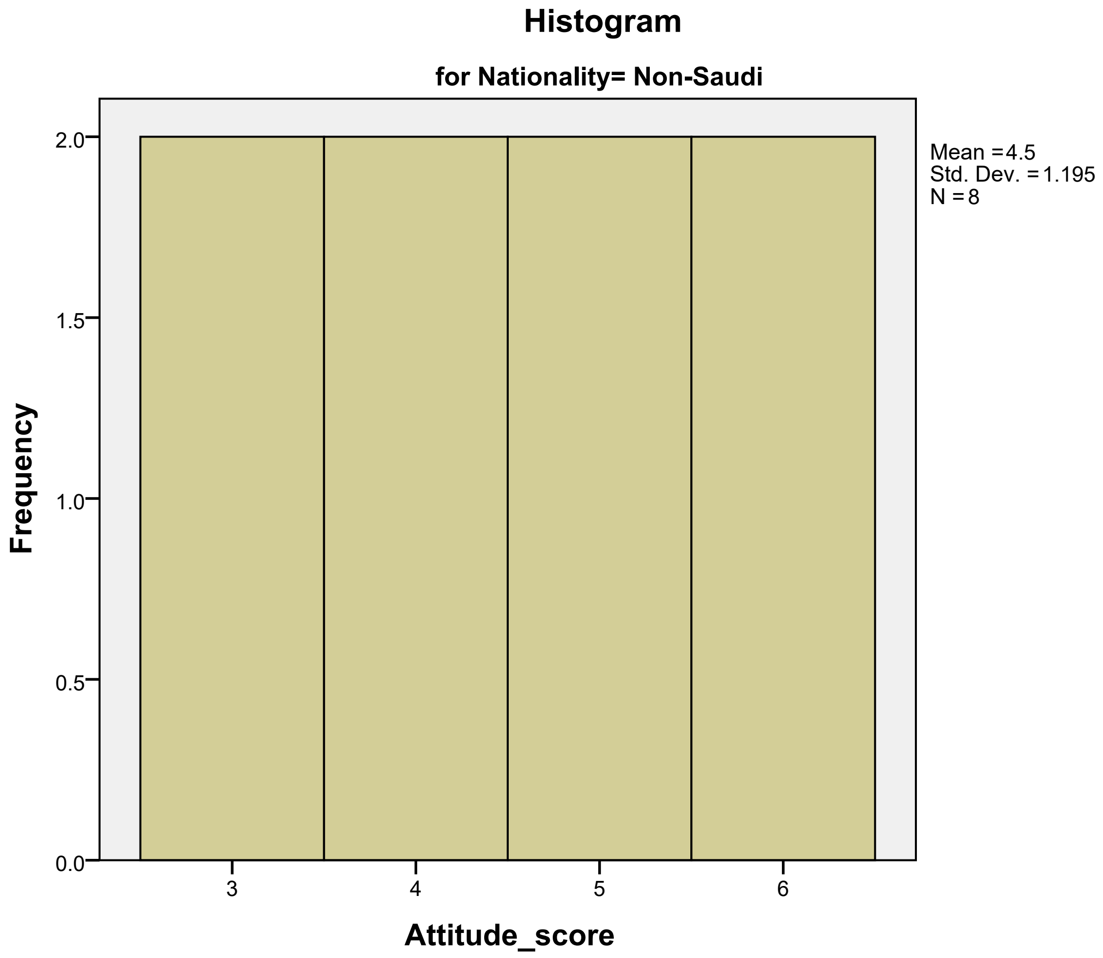

Normal Q-Q Plots

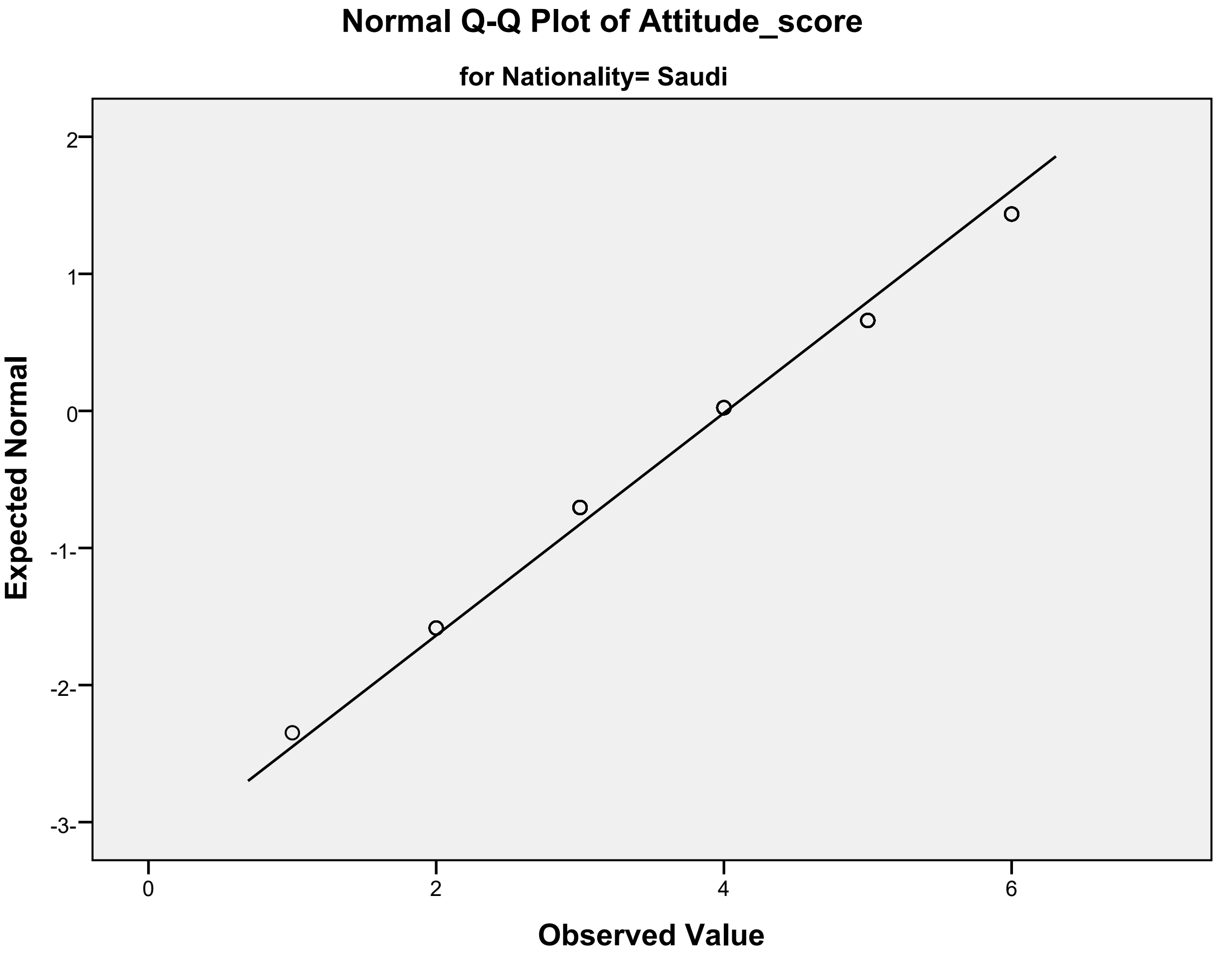
Normal Q-Q Plot of Attitude_score
for Nationality= Saudi
Expected Normal
2
1
0
-1
-2
-3
0
2
4
6
Observed Value

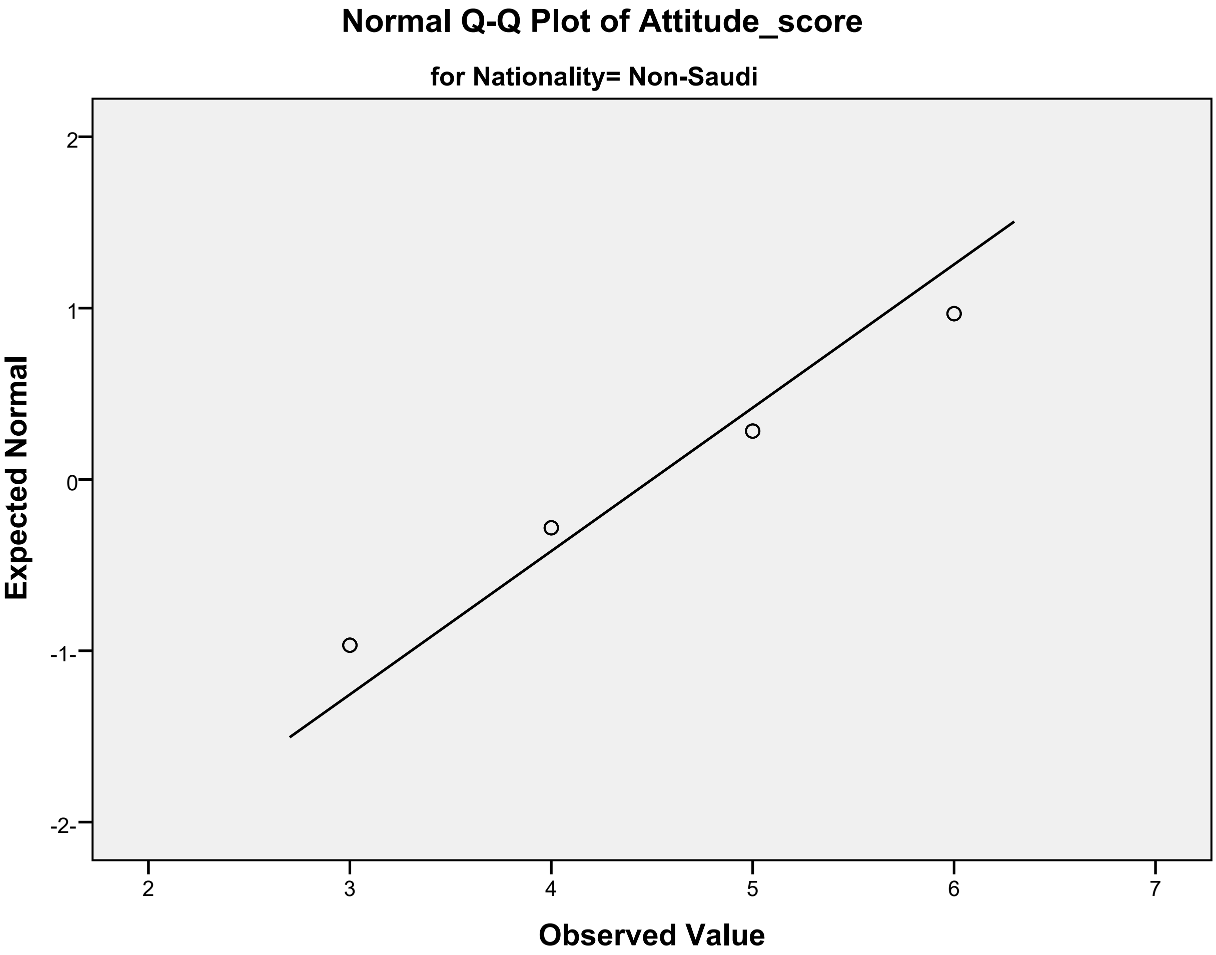

Detrended Normal Q-Q Plots

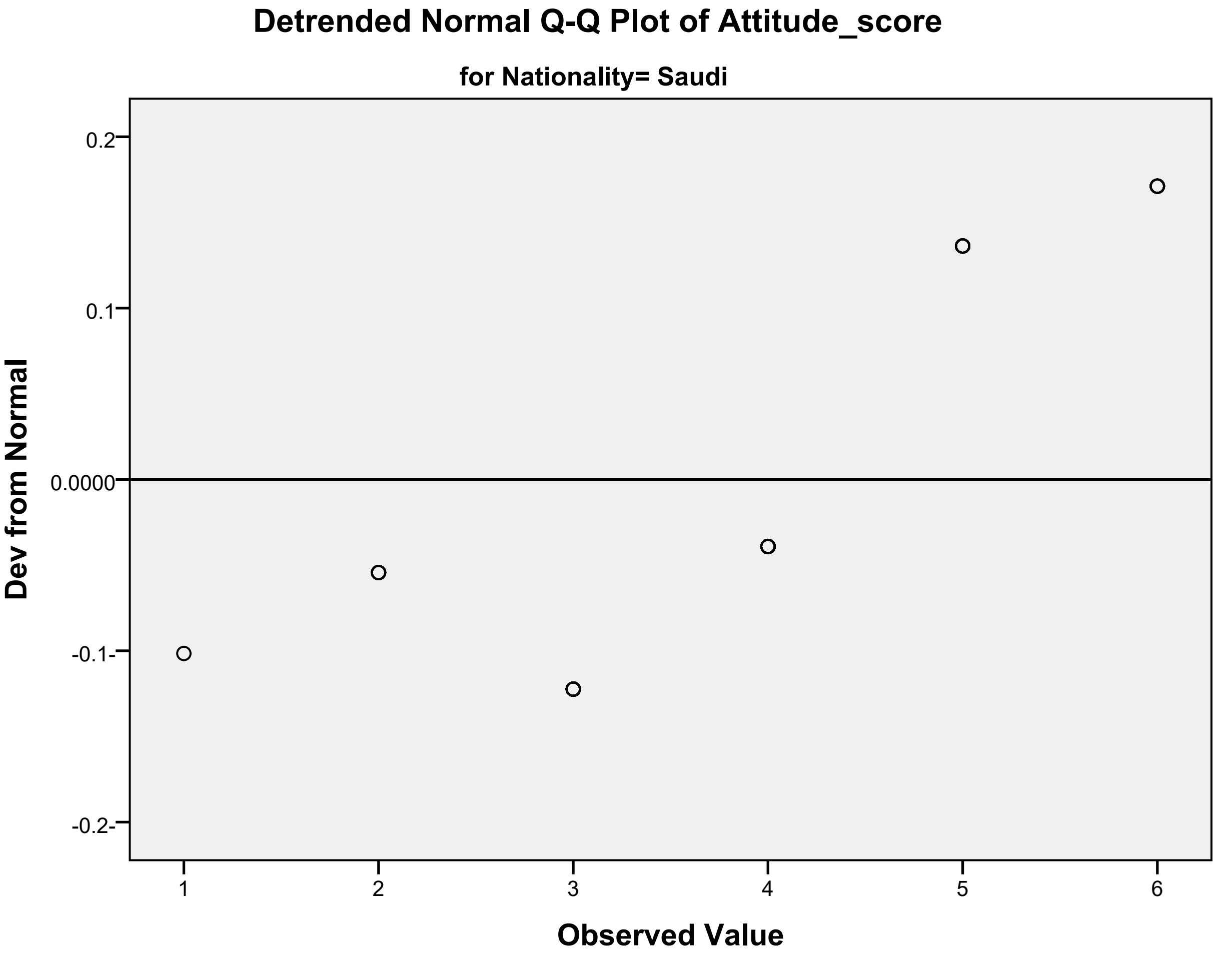

Detrended Normal Q-Q Plot of Attitude_score
for Nationality= Saudi
Dev from Normal
0.2
0.1
0.0000
-0.1
-0.2
1
2
3
4
5
6
Observed Value

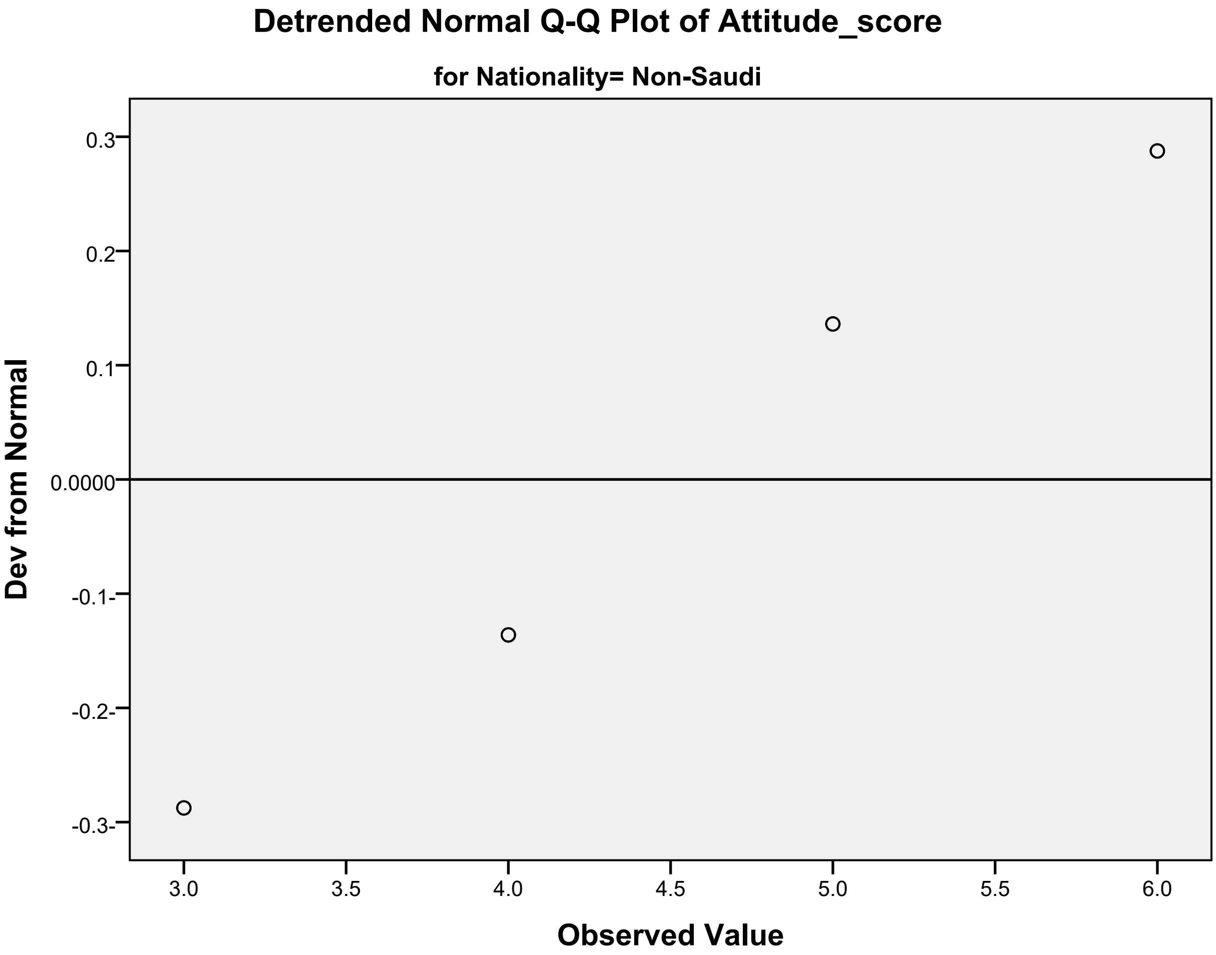

hospital_cat2

Case Processing Summary

		Cases					
		Valid		Missing		Total	
	hospital_cat2	N	Percent	N	Percent	N	Percent
Attitude_score	Academic & training centers	28	100.0%	0	0.0%	28	100.0%
	Other centers	85	100.0%	0	0.0%	85	100.0%

Descriptives

	hospital_cat2			Statistic	Std. Error
Attitude_score	Academic & training centers	Mean		4.68	.225
		95% Confidence Interval for Mean	Lower Bound	4.22	
			Upper Bound	5.14	
		5% Trimmed Mean		4.74	
		Median		5.00	
		Variance		1.411	
		Std. Deviation		1.188	
		Minimum		2	
		Maximum		6	
		Range		4	
		Interquartile Range		2	
		Skewness		-.460-	.441
		Kurtosis		-.758-	.858
	Other centers	Mean		3.85	.128
		95% Confidence Interval for Mean	Lower Bound	3.59	
			Upper Bound	4.10	
		5% Trimmed Mean		3.84	
		Median		4.00	
		Variance		1.393	
		Std. Deviation		1.180	
		Minimum		1	
		Maximum		6	
		Range		5	
		Interquartile Range		2	
		Skewness		.126	.261
		Kurtosis		-.626-	.517

Tests of Normality

		Kolmogorov-Smirnov[a]			Shapiro-Wilk		
	hospital_cat2	Statistic	df	Sig.	Statistic	df	Sig.
Attitude_score	Academic & training centers	.188	28	.012	.880	28	.004
	Other centers	.199	85	.000	.922	85	.000

a. Lilliefors Significance Correction

Attitude_score

Histograms

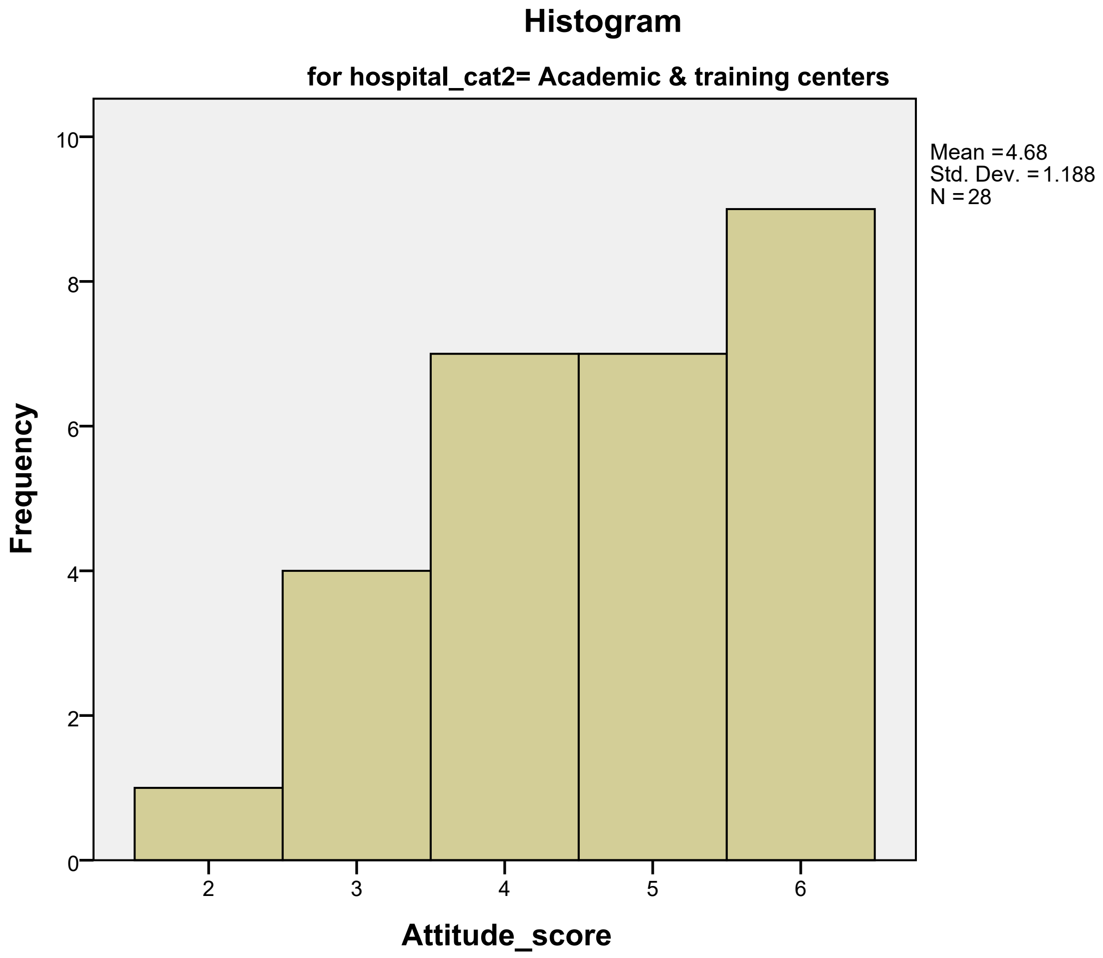

Histogram
for hospital_cat2= Academic & training centers
Mean =4.68
Std. Dev. =1.188
N =28
Frequency
0
2
4
6
8
10
2
3
4
5
6
Attitude_score

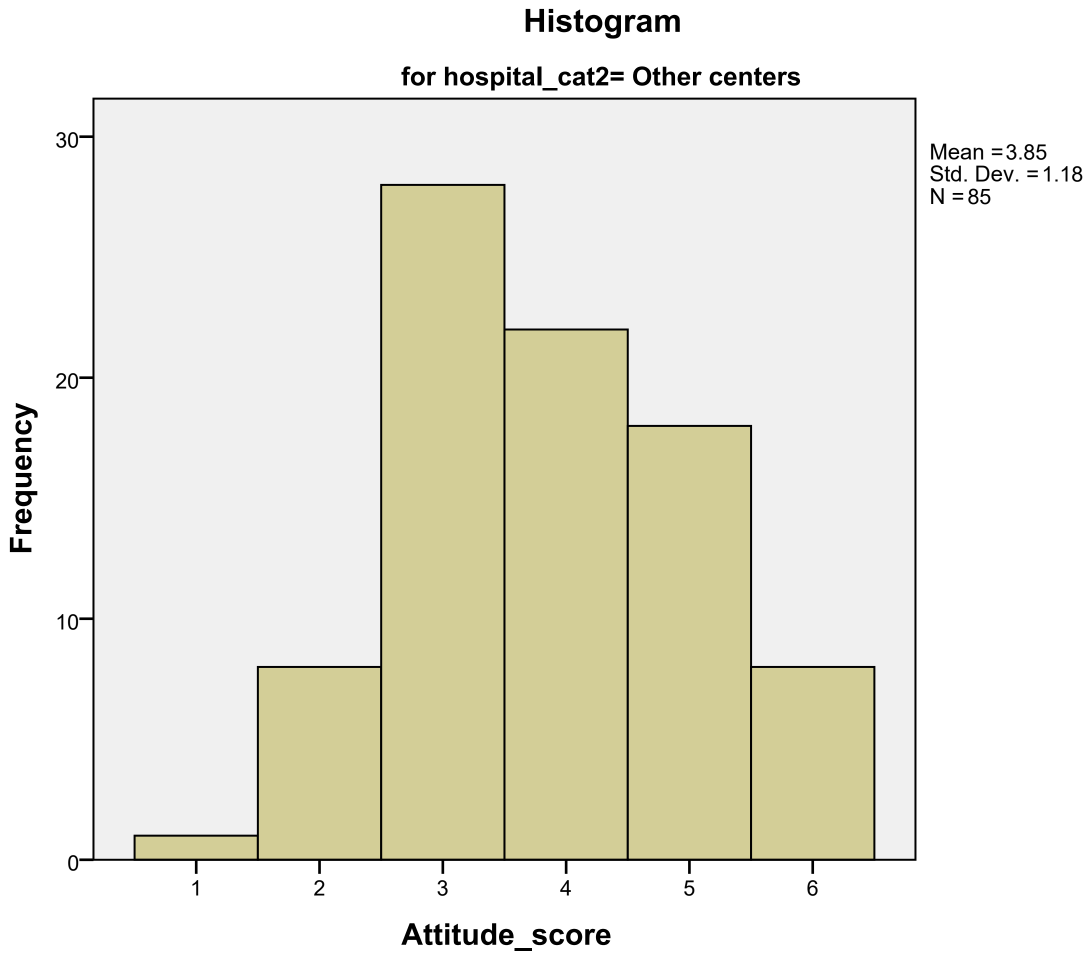

Normal Q-Q Plots

Normal Q-Q Plot of Attitude_score

for hospital_cat2= Academic & training centers

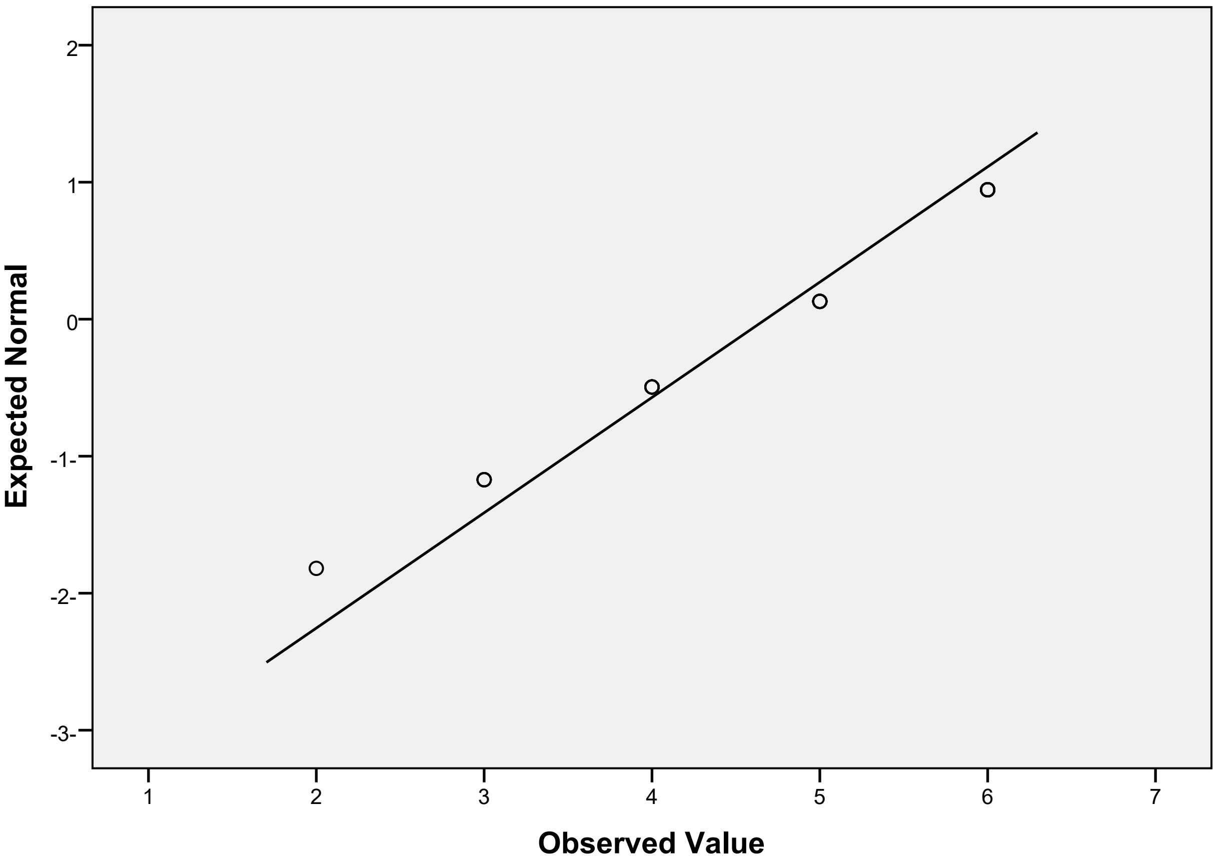

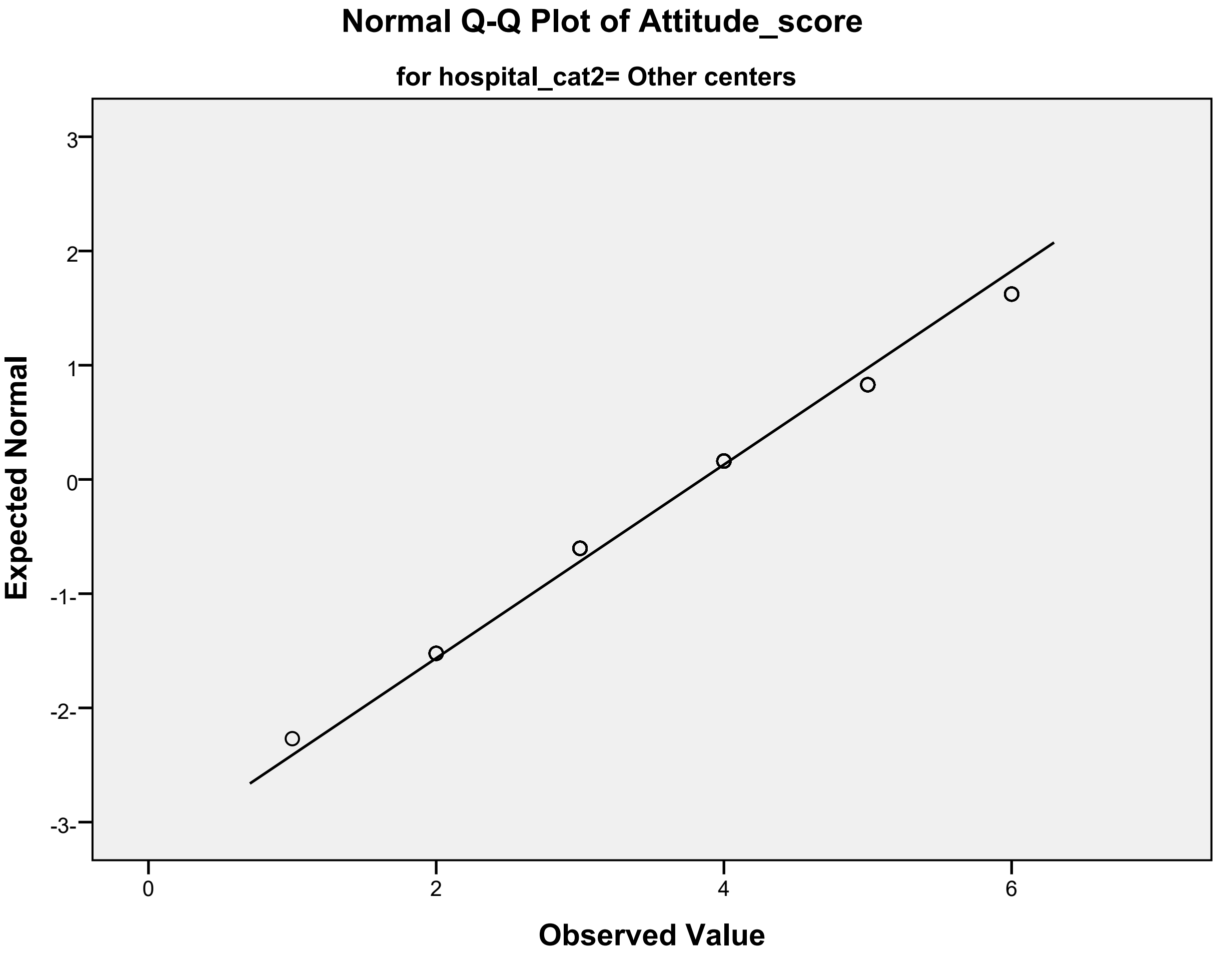

Detrended Normal Q-Q Plots

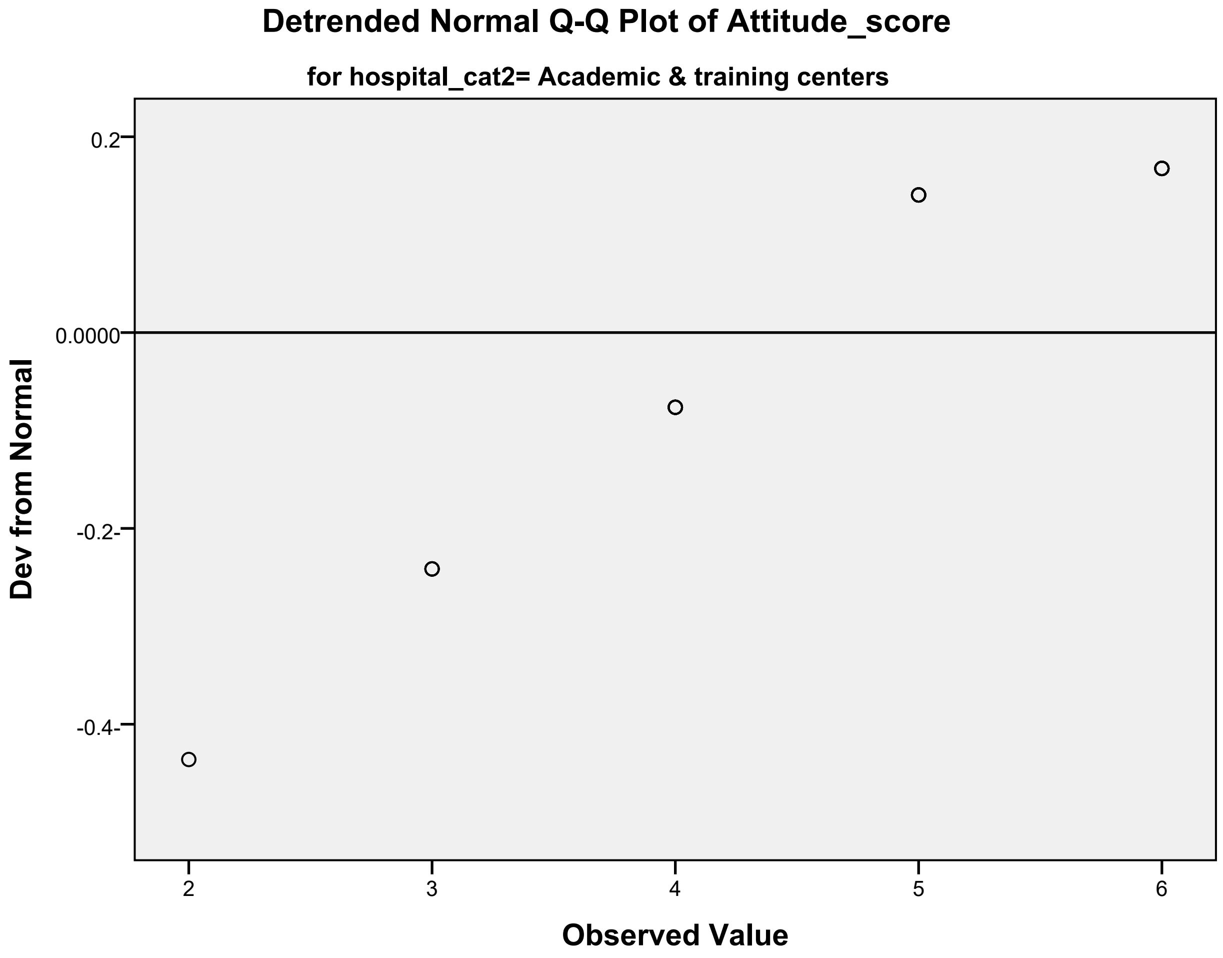
Detrended Normal Q-Q Plot of Attitude_score
for hospital_cat2= Academic & training centers
Dev from Normal
0.2
0.0000
-0.2
-0.4
2
3
4
5
6
Observed Value

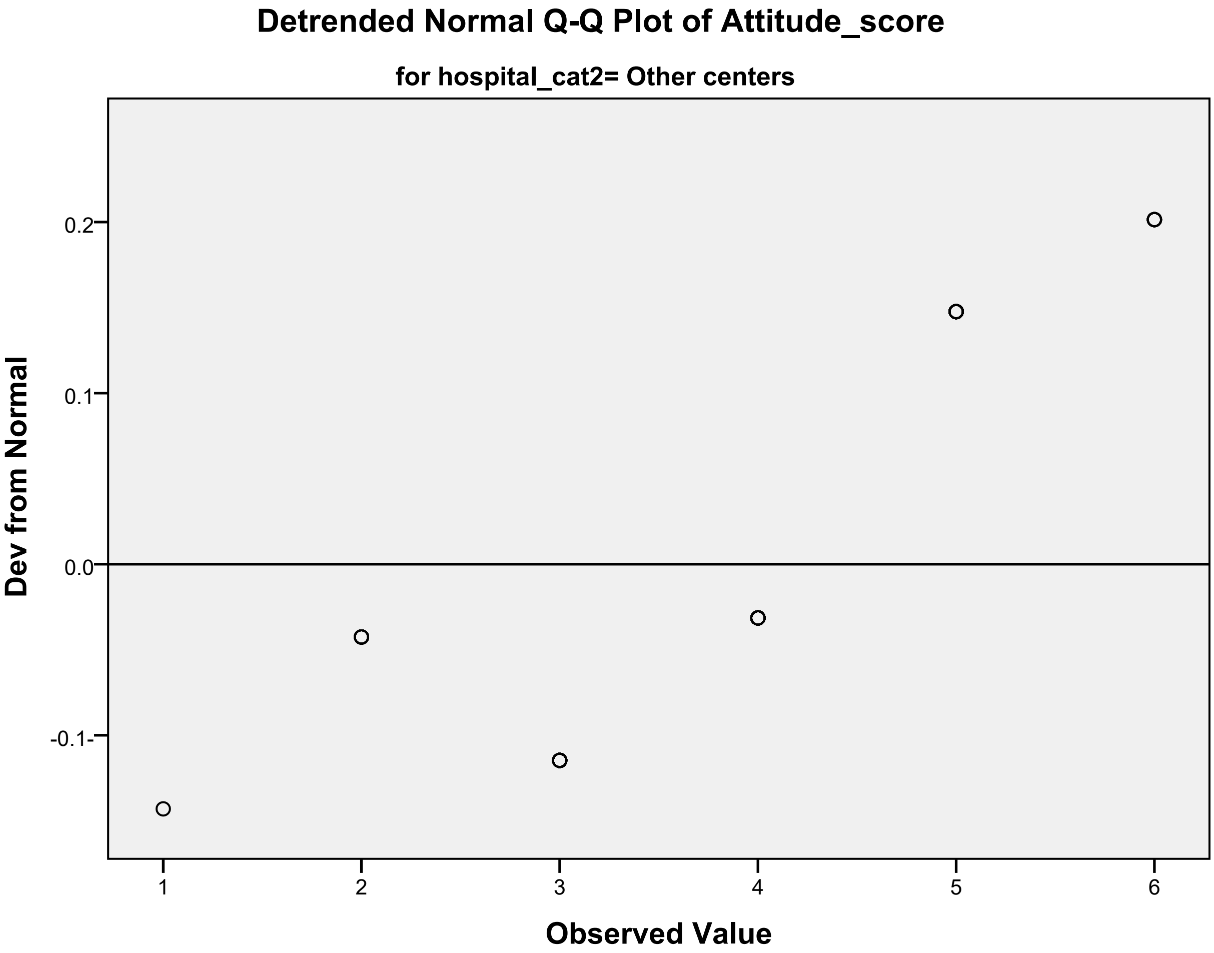
Detrended Normal Q-Q Plot of Attitude_score
for hospital_cat2= Other centers
Dev from Normal
0.2
0.1
0.0
-0.1
1
2
3
4
5
6
Observed Value

```
 GET
  FILE='C:\Users\Mosaab Lap\Desktop\SPSS Files\SPSS 419\Data 419.sav'.
DATASET NAME DataSet6 WINDOW=FRONT.
REGRESSION
  /MISSING LISTWISE
  /STATISTICS COEFF OUTS CI(95) R ANOVA COLLIN TOL
  /CRITERIA=PIN(.05) POUT(.10)
  /NOORIGIN
  /DEPENDENT Knowledge_score
  /METHOD=ENTER Female_gender Age_28_35 Age_36_45 Age_46_55 Age_gt55 Non_saudi
 Junior Senior
    Exp_2_5 Exp_6_10 Exp_11_15 Exp_gt15 Academic_hospitals
  /SCATTERPLOT=(*ZRESID ,*ZPRED)
  /RESIDUALS DURBIN HISTOGRAM(ZRESID) NORMPROB(ZRESID)
  /SAVE ZPRED COOK LEVER ZRESID.
```

Regression

Notes

Output Created		19-SEP-2026 23:34:45
Comments		
Input	Data	C:\Users\Mosaab Lap\Desktop\SPSS Files\SPSS 419\Data 419.sav
	Active Dataset	DataSet6
	Filter	<none>
	Weight	<none>
	Split File	<none>
	N of Rows in Working Data File	113
Missing Value Handling	Definition of Missing	User-defined missing values are treated as missing.
	Cases Used	Statistics are based on cases with no missing values for any variable used.

Notes

Syntax		REGRESSION<br>/MISSING LISTWISE<br>/STATISTICS COEFF OUTS CI(95) R ANOVA COLLIN TOL<br>/CRITERIA=PIN(.05) POUT(.10)<br>/NOORIGIN<br>/DEPENDENT Knowledge_score<br>/METHOD=ENTER Female_gender Age_28_35 Age_36_45 Age_46_55 Age_gt55 Non_saudi Junior Senior<br>Exp_2_5 Exp_6_10 Exp_11_15 Exp_gt15 Academic_hospitals<br>/SCATTERPLOT=(*ZRESID , *ZPRED)<br>/RESIDUALS DURBIN HISTOGRAM(ZRESID) NORMPROB(ZRESID)<br>/SAVE ZPRED COOK LEVER ZRESID.
Resources	Processor Time	00:00:00.30
	Elapsed Time	00:00:00.27
	Memory Required	60320 bytes
	Additional Memory Required for Residual Plots	488 bytes
Variables Created or Modified	ZPR_1	Standardized Predicted Value
	ZRE_1	Standardized Residual
	COO_1	Cook's Distance
	LEV_1	Centered Leverage Value

[DataSet6] C:\Users\Mosaab Lap\Desktop\SPSS Files\SPSS 419\Data 419.sav

Variables Entered/Removed[a]

Model	Variables Entered	Variables Removed	Method
1	Academic hospital (ref: Other centers), Senior trainee (ref: Consultant), Experience 11–15 years (ref: <2), Female (ref: Male), Age 46–55 (ref: <28), Experience 6–10 years (ref: <2), Non-Saudi (ref: Saudi), Experience 2–5 years (ref: <2), Age >55 (ref: <28), Age 28–35 (ref: <28), Experience >15 years (ref: <2), Junior trainee (ref: Consultant), Age 36–45 (ref: <28)[b]	.	Enter

a. Dependent Variable: Knowledge_score

b. All requested variables entered.

Model Summary[b]

Model	R	R Square	Adjusted R Square	Std. Error of the Estimate	Durbin-Watson
1	.403[a]	.162	.052	3.236	2.024

a. Predictors: (Constant), Academic hospital (ref: Other centers), Senior trainee (ref: Consultant), Experience 11–15 years (ref: <2), Female (ref: Male), Age 46–55 (ref: <28), Experience 6–10 years (ref: <2), Non-Saudi (ref: Saudi), Experience 2–5 years (ref: <2), Age >55 (ref: <28), Age 28–35 (ref: <28), Experience >15 years (ref: <2), Junior trainee (ref: Consultant), Age 36–45 (ref: <28)

b. Dependent Variable: Knowledge_score

ANOVA[a]

Model		Sum of Squares	df	Mean Square	F	Sig.
1	Regression	200.928	13	15.456	1.476	.140[b]
	Residual	1036.912	99	10.474		
	Total	1237.841	112			

a. Dependent Variable: Knowledge_score

b. Predictors: (Constant), Academic hospital (ref: Other centers), Senior trainee (ref: Consultant), Experience 11–15 years (ref: <2), Female (ref: Male), Age 46–55 (ref: <28), Experience 6–10 years (ref: <2), Non-Saudi (ref: Saudi), Experience 2–5 years (ref: <2), Age >55 (ref: <28), Age 28–35 (ref: <28), Experience >15 years (ref: <2), Junior trainee (ref: Consultant), Age 36–45 (ref: <28)

Coefficients[a]

Model		Unstandardized Coefficients		Standardized Coefficients	t	Sig.	95.0% Confidence Interval for B		Collinearity Statistics	
		B	Std. Error	Beta			Lower Bound	Upper Bound	Tolerance	VIF
1	(Constant)	12.919	1.827		7.072	.000	9.295	16.544		
	Female (ref: Male)	1.217	.689	.182	1.767	.080	-.150-	2.584	.799	1.251
	Age 28–35 (ref: <28)	.007	1.108	.001	.007	.995	-2.191-	2.205	.320	3.120
	Age 36–45 (ref: <28)	1.340	2.078	.163	.645	.520	-2.783-	5.464	.132	7.553
	Age 46–55 (ref: <28)	3.984	2.806	.222	1.420	.159	-1.584-	9.551	.345	2.900
	Age >55 (ref: <28)	1.743	3.095	.118	.563	.575	-4.397-	7.884	.192	5.195
	Non-Saudi (ref: Saudi)	-.963-	1.583	-.075-	-.609-	.544	-4.104-	2.177	.562	1.778
	Junior trainee (ref: Consultant)	-4.243-	1.671	-.628-	-2.539-	.013	-7.558-	-.927-	.139	7.218
	Senior trainee (ref: Consultant)	-.656-	1.461	-.065-	-.449-	.655	-3.555-	2.243	.400	2.500
	Experience 2–5 years (ref: <2)	-.154-	1.100	-.021-	-.140-	.889	-2.336-	2.027	.378	2.648
	Experience 6–10 years (ref: <2)	-3.367-	1.790	-.345-	-1.881-	.063	-6.918-	.184	.251	3.978
	Experience 11–15 years (ref: <2)	-4.527-	2.515	-.489-	-1.800-	.075	-9.519-	.464	.115	8.725
	Experience >15 years (ref: <2)	-6.866-	2.995	-.615-	-2.293-	.024	-12.808-	-.923-	.118	8.503
	Academic hospital (ref: Other centers)	.982	.894	.128	1.098	.275	-.793-	2.757	.622	1.609

a. Dependent Variable: Knowledge_score

Collinearity Diagnostics[a]

Model	Dimension	Eigenvalue	Condition Index	Variance Proportions													
				(Constant)	Female (ref: Male)	Age 28–35 (ref: <28)	Age 36–45 (ref: <28)	Age 46–55 (ref: <28)	Age >55 (ref: <28)	Non-Saudi (ref: Saudi)	Junior trainee (ref: Consultant)	Senior trainee (ref: Consultant)	Experience 2–5 years (ref: <2)	Experience 6–10 years (ref: <2)	Experience 11–15 years (ref: <2)	Experience >15 years (ref: <2)	Academic hospital (ref: Other centers)
1	1	4.167	1.000	.00	.01	.00	.00	.00	.00	.01	.00	.00	.00	.00	.00	.00	.01
	2	2.425	1.311	.00	.00	.01	.00	.00	.01	.02	.00	.00	.01	.00	.00	.01	.02
	3	1.760	1.539	.00	.00	.00	.02	.00	.01	.01	.00	.00	.00	.00	.02	.00	.00
	4	1.320	1.777	.00	.00	.00	.00	.03	.00	.03	.00	.07	.00	.05	.01	.00	.00
	5	1.006	2.035	.00	.05	.00	.01	.15	.02	.02	.00	.00	.02	.02	.00	.00	.00
	6	.918	2.130	.00	.07	.02	.00	.09	.00	.00	.01	.08	.03	.01	.00	.00	.00
	7	.740	2.372	.00	.05	.01	.00	.00	.00	.21	.00	.05	.00	.06	.00	.00	.16
	8	.509	2.863	.00	.23	.02	.00	.00	.00	.36	.00	.12	.01	.01	.00	.02	.00
	9	.380	3.312	.00	.03	.02	.01	.03	.03	.04	.01	.06	.03	.06	.00	.02	.47
	10	.321	3.605	.00	.04	.02	.07	.00	.15	.03	.00	.00	.03	.00	.06	.06	.01
	11	.248	4.095	.01	.40	.00	.00	.02	.02	.04	.04	.12	.20	.00	.00	.00	.30
	12	.157	5.150	.00	.04	.56	.00	.04	.09	.05	.00	.04	.42	.10	.01	.05	.00
	13	.034	11.052	.04	.06	.33	.89	.63	.66	.00	.02	.02	.19	.27	.50	.49	.01
	14	.016	15.989	.94	.01	.00	.00	.01	.01	.19	.91	.43	.03	.41	.40	.33	.02

a. Dependent Variable: Knowledge_score

Residuals Statistics[a]

	Minimum	Maximum	Mean	Std. Deviation	N
Predicted Value	5.32	14.57	9.65	1.339	113
Std. Predicted Value	-3.232-	3.680	.000	1.000	113
Standard Error of Predicted Value	.620	2.278	1.056	.430	113
Adjusted Predicted Value	4.74	13.95	9.65	1.458	113
Residual	-8.103-	8.261	.000	3.043	113
Std. Residual	-2.504-	2.553	.000	.940	113
Stud. Residual	-2.810-	2.717	.000	1.006	113
Deleted Residual	-10.204-	9.361	-.001-	3.505	113
Stud. Deleted Residual	-2.914-	2.810	.001	1.016	113
Mahal. Distance	3.119	54.506	12.885	11.562	113
Cook's Distance	.000	.146	.011	.022	113
Centered Leverage Value	.028	.487	.115	.103	113

a. Dependent Variable: Knowledge_score

Charts

Histogram

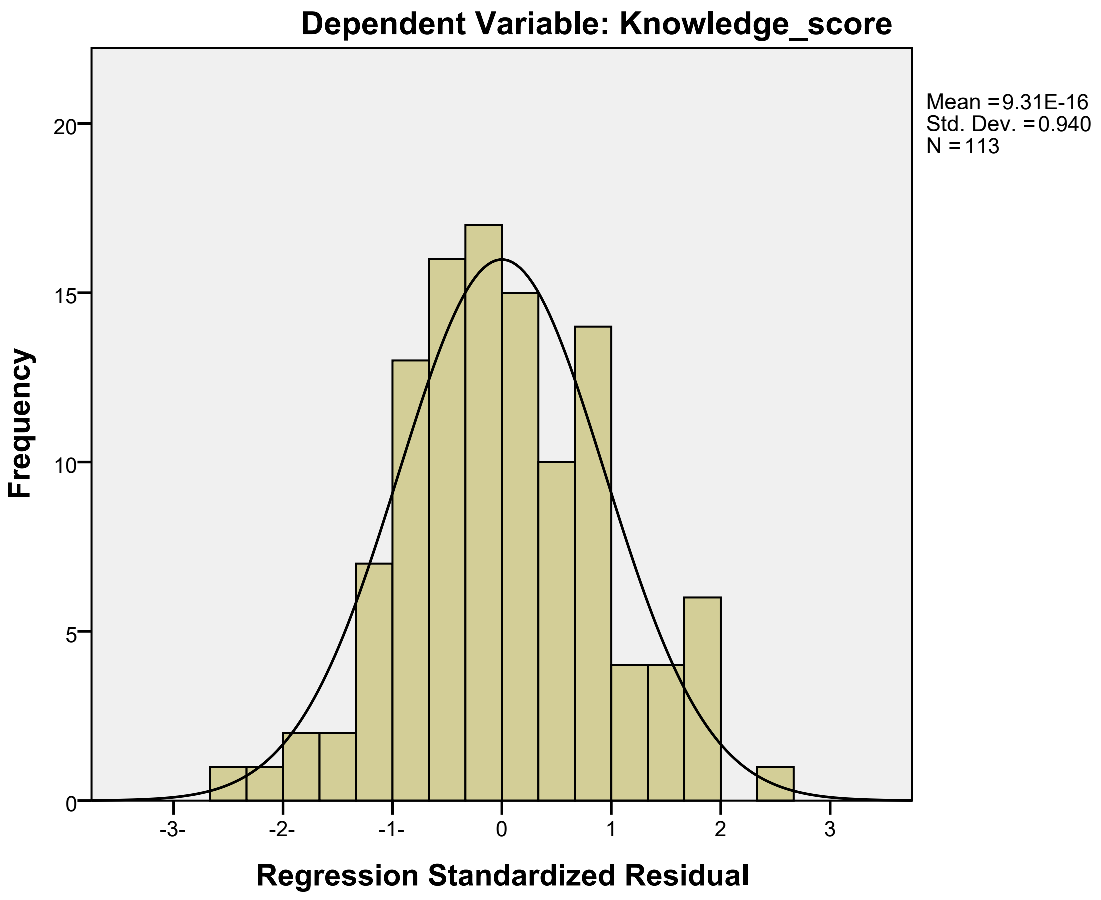

Dependent Variable: Knowledge_score
Mean = 9.31E-16
Std. Dev. = 0.940
N = 113
Frequency
0
5
10
15
20
-3
-2
-1
0
1
2
3
Regression Standardized Residual

Normal P-P Plot of Regression Standardized Residual

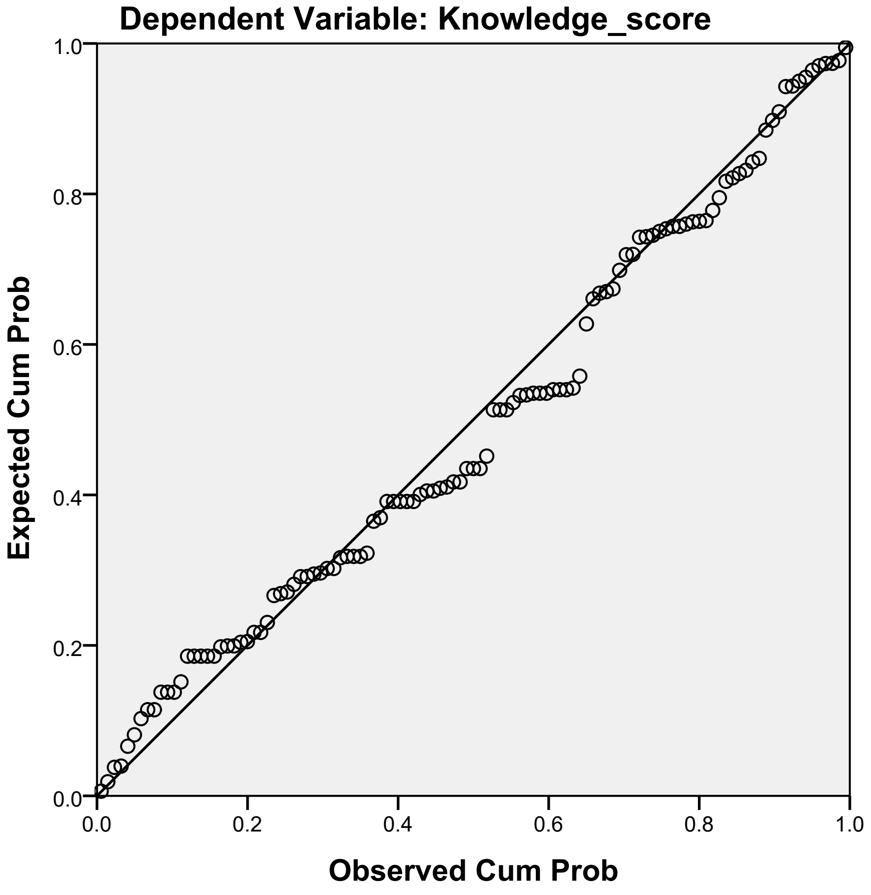

Scatterplot

Dependent Variable: Knowledge_score

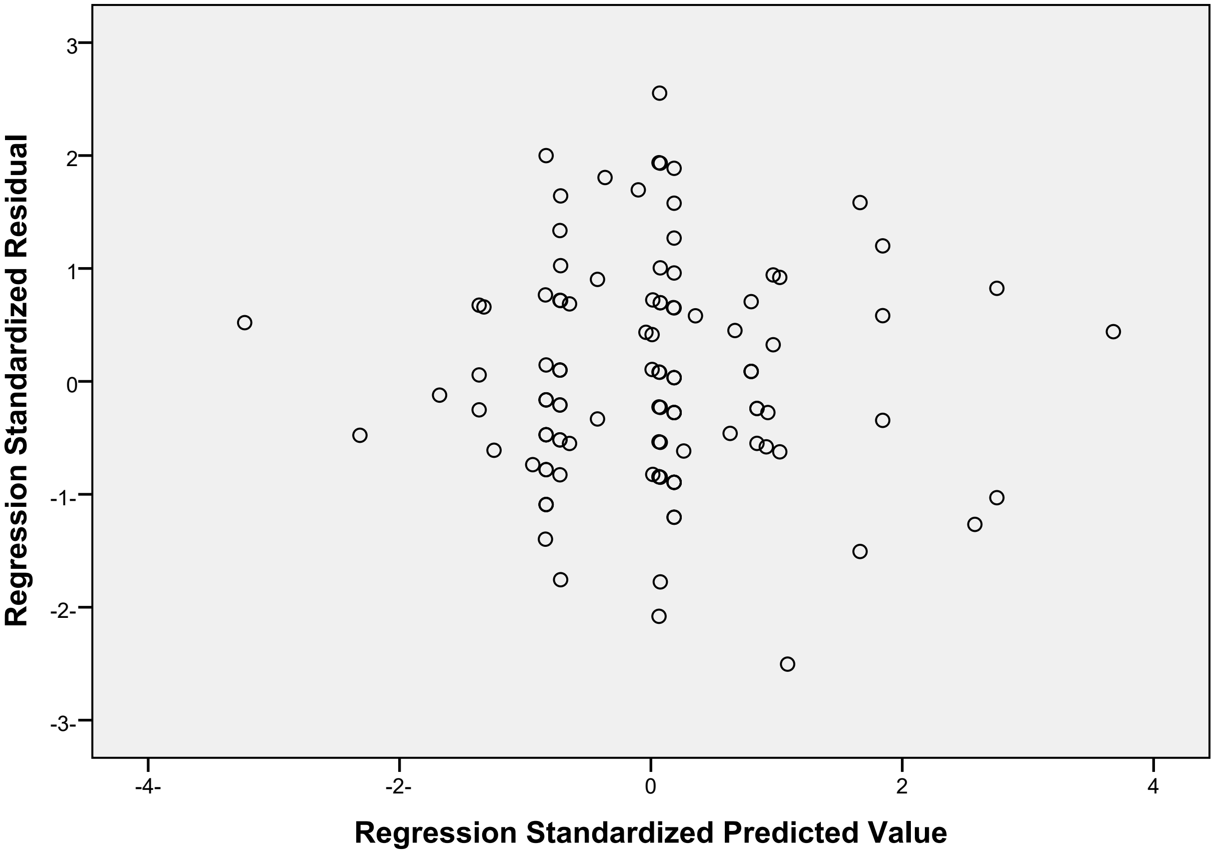

```
REGRESSION
 /MISSING LISTWISE
 /STATISTICS COEFF OUTS CI(95) R ANOVA COLLIN TOL
 /CRITERIA=PIN(.05) POUT(.10)
 /NOORIGIN
 /DEPENDENT Knowledge_score
 /METHOD=ENTER Female_gender Age_28_35 Age_36_45 Age_46_55 Age_gt55 Non_saudi
Junior Senior
   Academic_hospitals
 /SCATTERPLOT=(*ZRESID ,*ZPRED)
 /RESIDUALS DURBIN HISTOGRAM(ZRESID) NORMPROB(ZRESID)
 /SAVE ZPRED COOK LEVER ZRESID.
```

Regression

Notes

Output Created		19-SEP-2026 23:46:46
Comments		
Input	Data	C:\Users\Mosaab Lap\Desktop\SPSS Files\SPSS 419\Data 419.sav
	Active Dataset	DataSet6
	Filter	<none>
	Weight	<none>
	Split File	<none>
	N of Rows in Working Data File	113
Missing Value Handling	Definition of Missing	User-defined missing values are treated as missing.
	Cases Used	Statistics are based on cases with no missing values for any variable used.

Notes

Syntax		REGRESSION<br>/MISSING LISTWISE<br>/STATISTICS COEFF OUTS CI(95) R ANOVA COLLIN TOL<br>/CRITERIA=PIN(.05) POUT(.10)<br>/NOORIGIN<br>/DEPENDENT Knowledge_score<br>/METHOD=ENTER Female_gender Age_28_35 Age_36_45 Age_46_55 Age_gt55 Non_saudi Junior Senior Academic_hospitals<br>/SCATTERPLOT=(*ZRESID ,*ZPRED)<br>/RESIDUALS DURBIN HISTOGRAM(ZRESID) NORMPROB(ZRESID)<br>/SAVE ZPRED COOK LEVER ZRESID.
Resources	Processor Time	00:00:00.34
	Elapsed Time	00:00:00.26
	Memory Required	56192 bytes
	Additional Memory Required for Residual Plots	552 bytes
Variables Created or Modified	ZPR_2	Standardized Predicted Value
	ZRE_2	Standardized Residual
	COO_2	Cook's Distance
	LEV_2	Centered Leverage Value

Variables Entered/Removed[a]

Model	Variables Entered	Variables Removed	Method
1	Academic hospital (ref: Other centers), Senior trainee (ref: Consultant), Age 36–45 (ref: <28), Female (ref: Male), Age 46–55 (ref: <28), Non-Saudi (ref: Saudi), Age 28–35 (ref: <28), Age >55 (ref: <28), Junior trainee (ref: Consultant)[b]	.	Enter

a. Dependent Variable: Knowledge_score

b. All requested variables entered.

Model Summary[b]

Model	R	R Square	Adjusted R Square	Std. Error of the Estimate	Durbin-Watson
1	.331[a]	.109	.032	3.272	2.033

a. Predictors: (Constant), Academic hospital (ref: Other centers), Senior trainee (ref: Consultant), Age 36–45 (ref: <28), Female (ref: Male), Age 46–55 (ref: <28), Non-Saudi (ref: Saudi), Age 28–35 (ref: <28), Age >55 (ref: <28), Junior trainee (ref: Consultant)

b. Dependent Variable: Knowledge_score

ANOVA[a]

Model		Sum of Squares	df	Mean Square	F	Sig.
1	Regression	135.362	9	15.040	1.405	.196[b]
	Residual	1102.479	103	10.704		
	Total	1237.841	112			

a. Dependent Variable: Knowledge_score

b. Predictors: (Constant), Academic hospital (ref: Other centers), Senior trainee (ref: Consultant), Age 36–45 (ref: <28), Female (ref: Male), Age 46–55 (ref: <28), Non-Saudi (ref: Saudi), Age 28–35 (ref: <28), Age >55 (ref: <28), Junior trainee (ref: Consultant)

Coefficients[a]

Model		Unstandardized Coefficients		Standardized Coefficients	t	Sig.	95.0% Confidence Interval for B		Collinearity Statistics	
		B	Std. Error	Beta			Lower Bound	Upper Bound	Tolerance	VIF
1	(Constant)	10.781	1.551		6.949	.000	7.705	13.858		
	Female (ref: Male)	.952	.681	.142	1.398	.165	-.399-	2.302	.836	1.196
	Age 28–35 (ref: <28)	-.265-	.827	-.039-	-.320-	.749	-1.905-	1.375	.588	1.702
	Age 36–45 (ref: <28)	-1.085-	1.460	-.132-	-.743-	.459	-3.981-	1.811	.274	3.650
	Age 46–55 (ref: <28)	.594	2.070	.033	.287	.774	-3.510-	4.699	.648	1.544
	Age >55 (ref: <28)	-1.922-	2.242	-.130-	-.857-	.393	-6.368-	2.524	.375	2.667
	Non-Saudi (ref: Saudi)	-2.237-	1.471	-.173-	-1.520-	.131	-5.155-	.681	.665	1.504
	Junior trainee (ref: Consultant)	-1.972-	1.336	-.292-	-1.476-	.143	-4.622-	.677	.221	4.515
	Senior trainee (ref: Consultant)	.478	1.374	.048	.348	.729	-2.248-	3.204	.462	2.165
	Academic hospital (ref: Other centers)	.601	.871	.078	.690	.492	-1.126-	2.328	.670	1.492

a. Dependent Variable: Knowledge_score

Collinearity Diagnostics[a]

Model	Dimension	Eigenvalue	Condition Index	Variance Proportions									
				(Constant)	Female (ref: Male)	Age 28–35 (ref: <28)	Age 36–45 (ref: <28)	Age 46–55 (ref: <28)	Age >55 (ref: <28)	Non-Saudi (ref: Saudi)	Junior trainee (ref: Consultant)	Senior trainee (ref: Consultant)	Academic hospital (ref: Other centers)
1	1	3.399	1.000	.00	.02	.01	.00	.00	.00	.01	.00	.01	.02
	2	1.668	1.428	.00	.01	.02	.00	.01	.06	.07	.01	.00	.05
	3	1.156	1.715	.00	.00	.00	.05	.07	.02	.00	.01	.13	.00
	4	1.029	1.818	.00	.00	.03	.04	.26	.07	.02	.00	.01	.01
	5	.939	1.903	.00	.00	.02	.06	.18	.02	.04	.00	.11	.01
	6	.767	2.105	.00	.17	.09	.00	.01	.00	.23	.00	.00	.15
	7	.446	2.762	.00	.40	.07	.02	.00	.05	.42	.01	.05	.03
	8	.330	3.210	.00	.01	.04	.04	.18	.30	.08	.00	.00	.65
	9	.244	3.734	.01	.29	.48	.02	.00	.01	.04	.09	.32	.01
	10	.024	11.972	.98	.09	.25	.76	.28	.47	.09	.87	.36	.07

a. Dependent Variable: Knowledge_score

Residuals Statistics[a]

	Minimum	Maximum	Mean	Std. Deviation	N
Predicted Value	7.22	12.93	9.65	1.099	113
Std. Predicted Value	-2.204-	2.986	.000	1.000	113
Standard Error of Predicted Value	.601	2.126	.908	.352	113
Adjusted Predicted Value	6.43	14.14	9.66	1.177	113
Residual	-9.547-	8.239	.000	3.137	113
Std. Residual	-2.918-	2.518	.000	.959	113
Stud. Residual	-3.152-	2.562	-.002-	1.004	113
Deleted Residual	-11.138-	8.527	-.016-	3.449	113
Stud. Deleted Residual	-3.300-	2.635	-.001-	1.015	113
Mahal. Distance	2.788	46.302	8.920	8.418	113
Cook's Distance	.000	.165	.010	.021	113
Centered Leverage Value	.025	.413	.080	.075	113

a. Dependent Variable: Knowledge_score

Charts

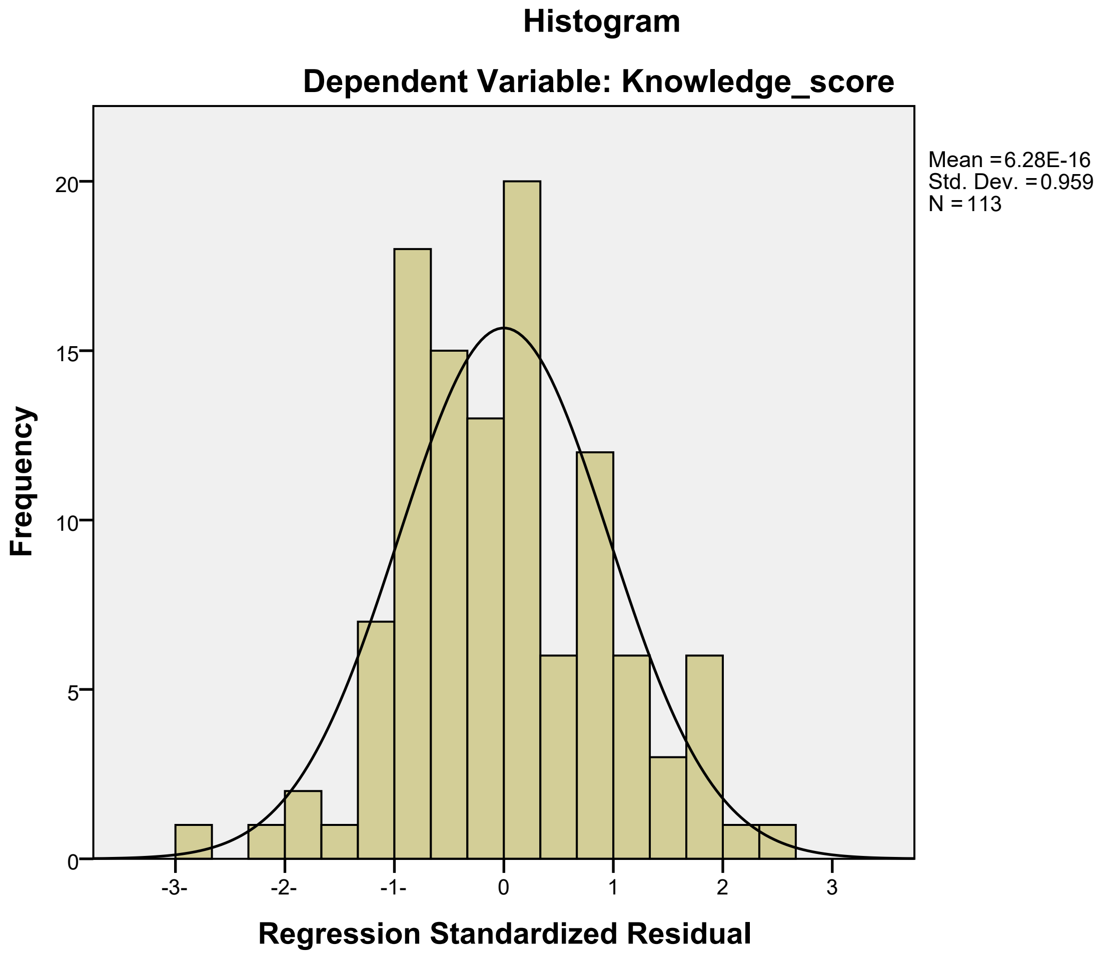
Histogram
Dependent Variable: Knowledge_score
Mean =6.28E-16
Std. Dev. =0.959
N =113
Frequency
0
5
10
15
20
-3-
-2-
-1-
0
1
2
3
Regression Standardized Residual

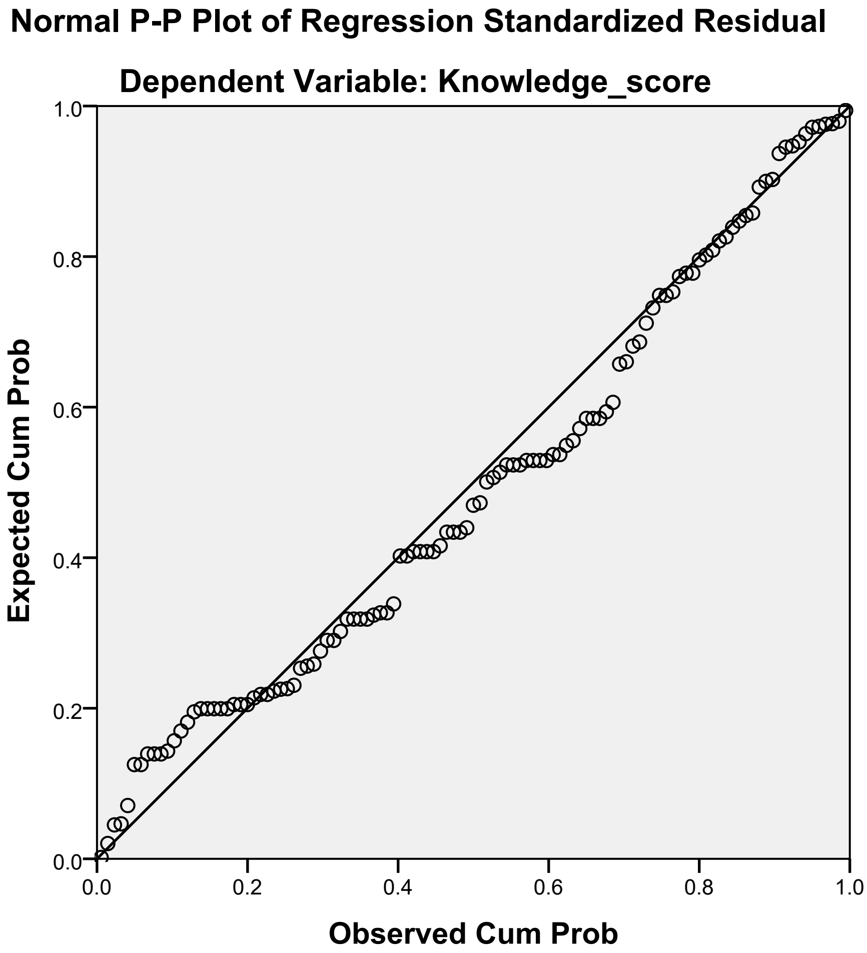

Normal P-P Plot of Regression Standardized Residual
Dependent Variable: Knowledge_score
Expected Cum Prob
Observed Cum Prob
0.0
0.2
0.4
0.6
0.8
1.0

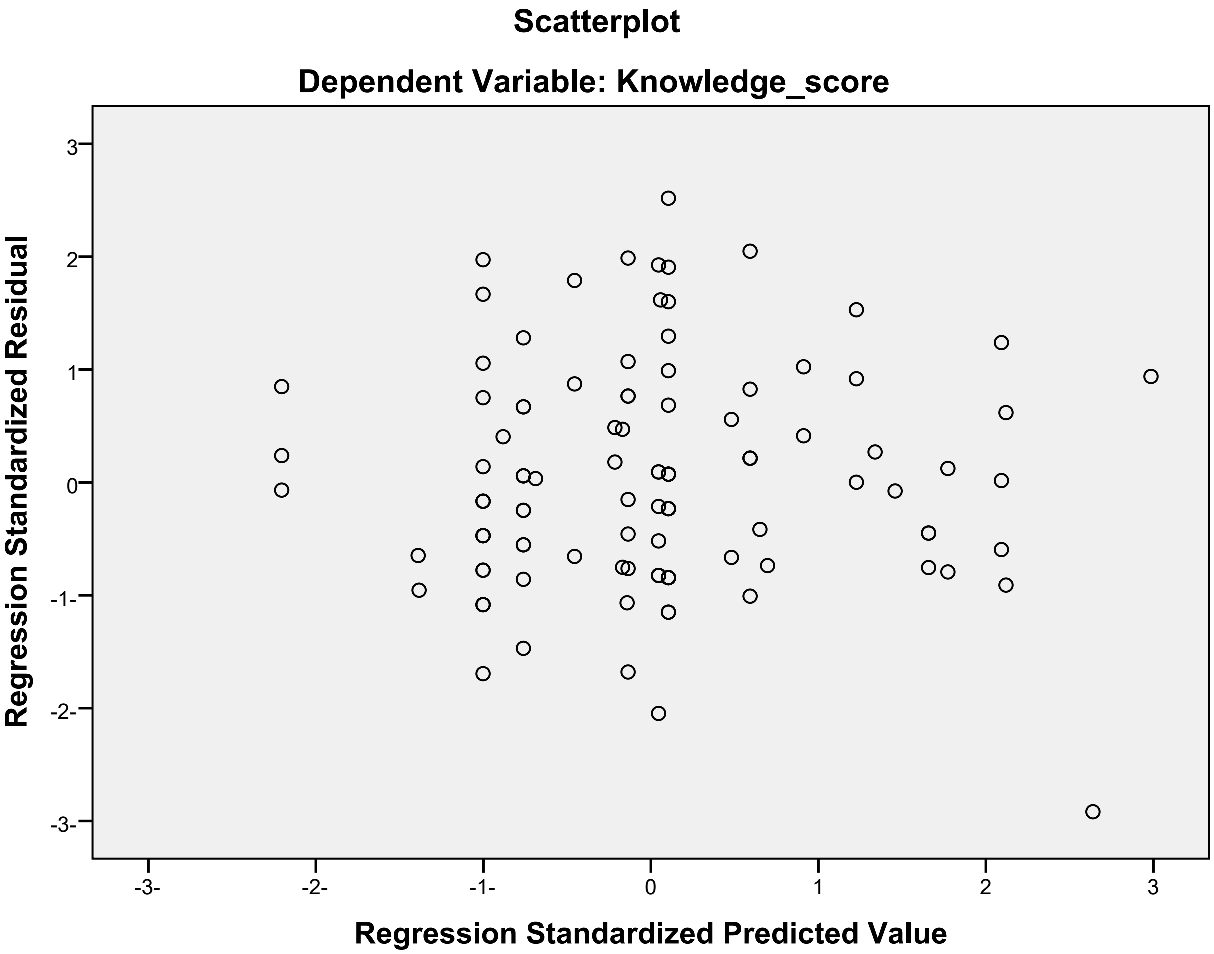


```
REGRESSION
  /MISSING LISTWISE
  /STATISTICS COEFF OUTS CI(95) R ANOVA COLLIN TOL
  /CRITERIA=PIN(.05) POUT(.10)
  /NOORIGIN
  /DEPENDENT Knowledge_score
  /METHOD=ENTER Female_gender Age_28_35 Age_36_45 Age_46_55 Age_gt55 Non_saudi
Academic_hospitals
    Exp_2_5 Exp_6_10 Exp_11_15 Exp_gt15
  /SCATTERPLOT=(*ZRESID ,*ZPRED)
  /RESIDUALS DURBIN HISTOGRAM(ZRESID) NORMPROB(ZRESID)
  /SAVE ZPRED COOK LEVER ZRESID.
```

Regression

Notes

Output Created		19-SEP-2026 23:47:41
Comments		
Input	Data	C:\Users\Mosaab Lap\Desktop\SPSS Files\SPSS 419\Data 419.sav
	Active Dataset	DataSet6
	Filter	<none>
	Weight	<none>
	Split File	<none>
	N of Rows in Working Data File	113
Missing Value Handling	Definition of Missing	User-defined missing values are treated as missing.
	Cases Used	Statistics are based on cases with no missing values for any variable used.
Syntax		REGRESSION<br>/MISSING LISTWISE<br>/STATISTICS COEFF OUTS CI(95) R ANOVA COLLIN TOL<br>/CRITERIA=PIN(.05) POUT(.10)<br>/NOORIGIN<br>/DEPENDENT Knowledge_score<br>/METHOD=ENTER Female_gender Age_28_35 Age_36_45 Age_46_55 Age_gt55 Non_saudi Academic_hospitals<br>Exp_2_5 Exp_6_10 Exp_11_15 Exp_gt15<br>/SCATTERPLOT=(*ZRESID , *ZPRED)<br>/RESIDUALS DURBIN HISTOGRAM(ZRESID) NORMPROB(ZRESID)<br>/SAVE ZPRED COOK LEVER ZRESID.
Resources	Processor Time	00:00:00.25
	Elapsed Time	00:00:00.22
	Memory Required	58368 bytes
	Additional Memory Required for Residual Plots	520 bytes
Variables Created or Modified	ZPR_3	Standardized Predicted Value
	ZRE_3	Standardized Residual
	COO_3	Cook's Distance
	LEV_3	Centered Leverage Value

Variables Entered/Removed[a]

Model	Variables Entered	Variables Removed	Method
1	Experience >15 years (ref: <2), Age 36–45 (ref: <28), Experience 6–10 years (ref: <2), Female (ref: Male), Age 46–55 (ref: <28), Experience 2–5 years (ref: <2), Non-Saudi (ref: Saudi), Academic hospital (ref: Other centers), Age 28–35 (ref: <28), Age >55 (ref: <28), Experience 11–15 years (ref: <2)[b]	.	Enter

a. Dependent Variable: Knowledge_score

b. All requested variables entered.

Model Summary[b]

Model	R	R Square	Adjusted R Square	Std. Error of the Estimate	Durbin-Watson
1	.279[a]	.078	-.022-	3.361	1.950

a. Predictors: (Constant), Experience >15 years (ref: <2), Age 36–45 (ref: <28), Experience 6–10 years (ref: <2), Female (ref: Male), Age 46–55 (ref: <28), Experience 2–5 years (ref: <2), Non-Saudi (ref: Saudi), Academic hospital (ref: Other centers), Age 28–35 (ref: <28), Age >55 (ref: <28), Experience 11–15 years (ref: <2)

b. Dependent Variable: Knowledge_score

ANOVA[a]

Model		Sum of Squares	df	Mean Square	F	Sig.
1	Regression	96.692	11	8.790	.778	.661[b]
	Residual	1141.149	101	11.299		
	Total	1237.841	112			

a. Dependent Variable: Knowledge_score

b. Predictors: (Constant), Experience >15 years (ref: <2), Age 36–45 (ref: <28), Experience 6–10 years (ref: <2), Female (ref: Male), Age 46–55 (ref: <28), Experience 2–5 years (ref: <2), Non-Saudi (ref: Saudi), Academic hospital (ref: Other centers), Age 28–35 (ref: <28), Age >55 (ref: <28), Experience 11–15 years (ref: <2)

Coefficients[a]

Model		Unstandardized Coefficients		Standardized Coefficients			95.0% Confidence Interval for B		Collinearity Statistics	
		B	Std. Error	Beta	t	Sig.	Lower Bound	Upper Bound	Tolerance	VIF
1	(Constant)	8.540	.731		11.689	.000	7.091	9.989		
	Female (ref: Male)	1.240	.714	.185	1.737	.085	-.176-	2.656	.803	1.245
	Age 28–35 (ref: <28)	.515	1.134	.075	.454	.651	-1.736-	2.765	.330	3.033
	Age 36–45 (ref: <28)	1.935	2.140	.235	.904	.368	-2.310-	6.180	.135	7.426
	Age 46–55 (ref: <28)	3.526	2.891	.197	1.219	.226	-2.210-	9.261	.350	2.854
	Age >55 (ref: <28)	2.295	3.121	.156	.735	.464	-3.896-	8.486	.204	4.898
	Non-Saudi (ref: Saudi)	-1.996-	1.449	-.155-	-1.378-	.171	-4.870-	.877	.724	1.381
	Academic hospital (ref: Other centers)	1.321	.922	.172	1.433	.155	-.508-	3.150	.631	1.584
	Experience 2–5 years (ref: <2)	.181	1.136	.025	.159	.874	-2.073-	2.436	.381	2.622
	Experience 6–10 years (ref: <2)	-.500-	1.541	-.051-	-.324-	.746	-3.557-	2.557	.366	2.735
	Experience 11–15 years (ref: <2)	-.989-	2.264	-.107-	-.437-	.663	-5.481-	3.502	.153	6.554
	Experience >15 years (ref: <2)	-2.865-	2.758	-.257-	-1.038-	.302	-8.336-	2.607	.150	6.686

a. Dependent Variable: Knowledge_score

Collinearity Diagnostics[a]

Model	Dimension	Eigenvalue	Condition Index	Variance Proportions											
				(Constant)	Female (ref: Male)	Age 28–35 (ref: <28)	Age 36–45 (ref: <28)	Age 46–55 (ref: <28)	Age >55 (ref: <28)	Non-Saudi (ref: Saudi)	Academic hospital (ref: Other centers)	Experience 2–5 years (ref: <2)	Experience 6–10 years (ref: <2)	Experience 11–15 years (ref: <2)	Experience >15 years (ref: <2)
1	1	3.648	1.000	.01	.01	.00	.00	.00	.00	.01	.02	.00	.00	.00	.00
	2	2.161	1.299	.00	.01	.02	.00	.00	.01	.02	.01	.02	.00	.00	.01
	3	1.724	1.455	.00	.00	.01	.02	.00	.01	.01	.00	.01	.00	.03	.00
	4	1.131	1.796	.00	.01	.00	.00	.05	.00	.05	.00	.03	.14	.01	.00
	5	1.002	1.908	.00	.03	.00	.01	.21	.02	.01	.00	.01	.01	.00	.00
	6	.739	2.221	.00	.20	.02	.00	.01	.00	.17	.17	.00	.05	.00	.00
	7	.596	2.474	.01	.22	.02	.00	.01	.00	.53	.02	.03	.00	.01	.01
	8	.345	3.250	.00	.02	.00	.04	.03	.00	.06	.56	.01	.07	.01	.11
	9	.319	3.382	.00	.00	.03	.03	.02	.22	.09	.14	.04	.05	.07	.03
	10	.167	4.669	.08	.02	.35	.00	.03	.08	.04	.03	.63	.15	.00	.04
	11	.134	5.210	.86	.42	.23	.00	.01	.00	.01	.05	.00	.00	.05	.04
	12	.034	10.409	.03	.05	.33	.89	.63	.64	.00	.00	.21	.51	.82	.76

a. Dependent Variable: Knowledge_score

Residuals Statistics[a]

	Minimum	Maximum	Mean	Std. Deviation	N
Predicted Value	7.30	13.64	9.65	.929	113
Std. Predicted Value	-2.530-	4.295	.000	1.000	113
Standard Error of Predicted Value	.643	2.269	1.014	.416	113
Adjusted Predicted Value	5.66	13.94	9.66	1.105	113
Residual	-8.115-	8.039	.000	3.192	113
Std. Residual	-2.414-	2.392	.000	.950	113
Stud. Residual	-2.611-	2.545	-.002-	1.010	113
Deleted Residual	-9.495-	9.344	-.018-	3.640	113
Stud. Deleted Residual	-2.691-	2.618	-.001-	1.020	113
Mahal. Distance	3.104	50.062	10.903	10.482	113
Cook's Distance	.000	.212	.013	.029	113
Centered Leverage Value	.028	.447	.097	.094	113

a. Dependent Variable: Knowledge_score

Charts

Histogram

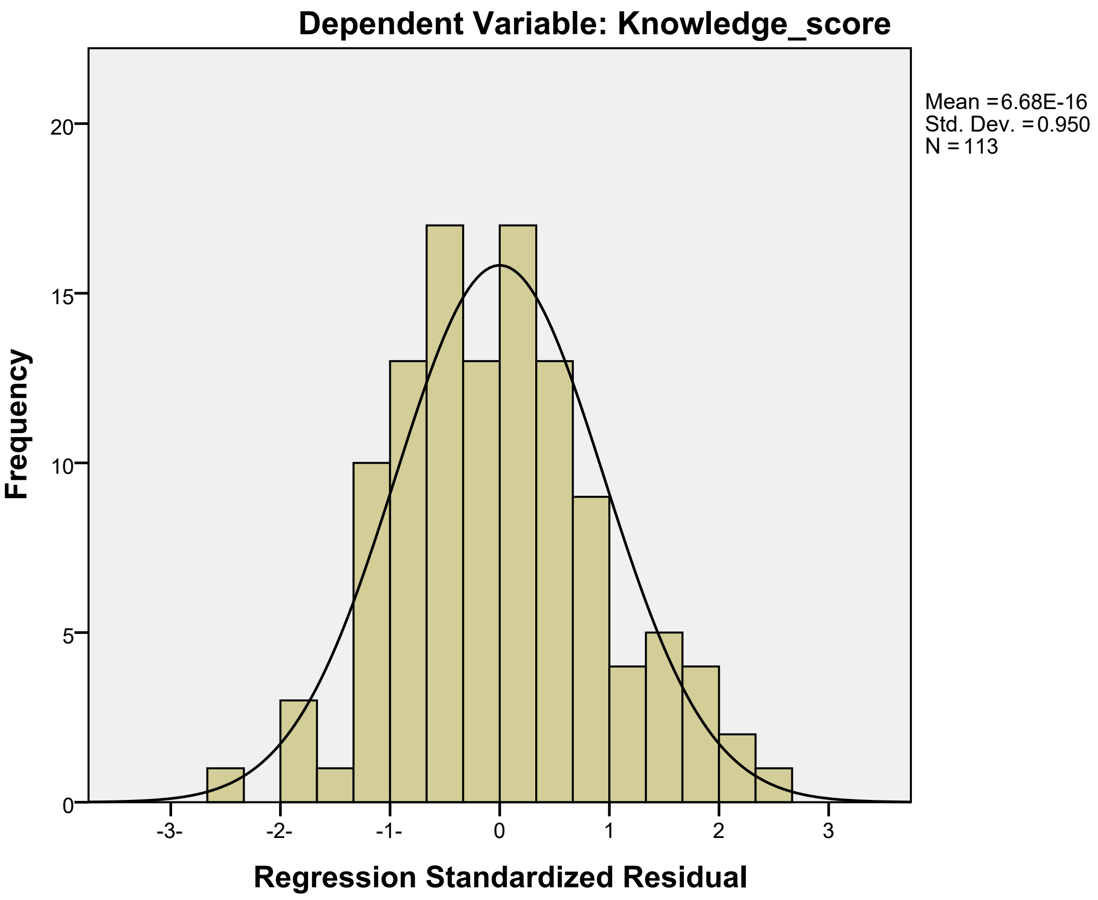

Dependent Variable: Knowledge_score
Mean =6.68E-16
Std. Dev. =0.950
N =113
Frequency
20
15
10
5
0
-3-
-2-
-1-
0
1
2
3
Regression Standardized Residual

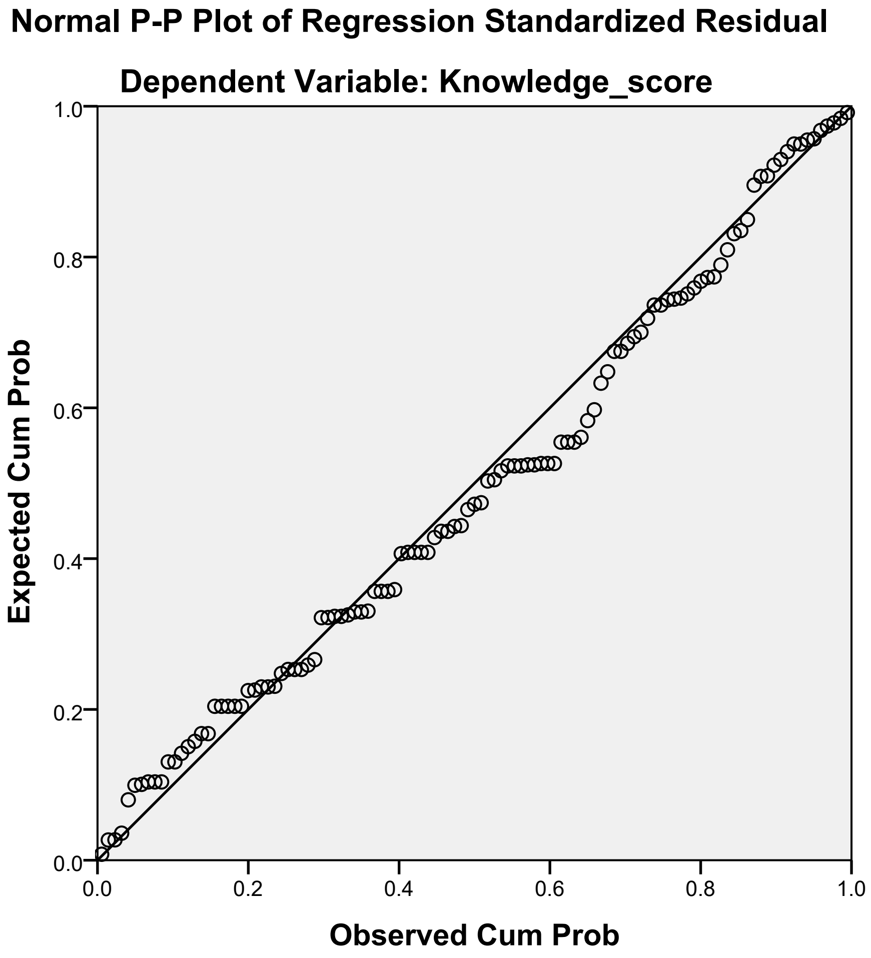
Normal P-P Plot of Regression Standardized Residual
Dependent Variable: Knowledge_score
Expected Cum Prob
Observed Cum Prob
0.0
0.2
0.4
0.6
0.8
1.0

Scatterplot

Dependent Variable: Knowledge_score

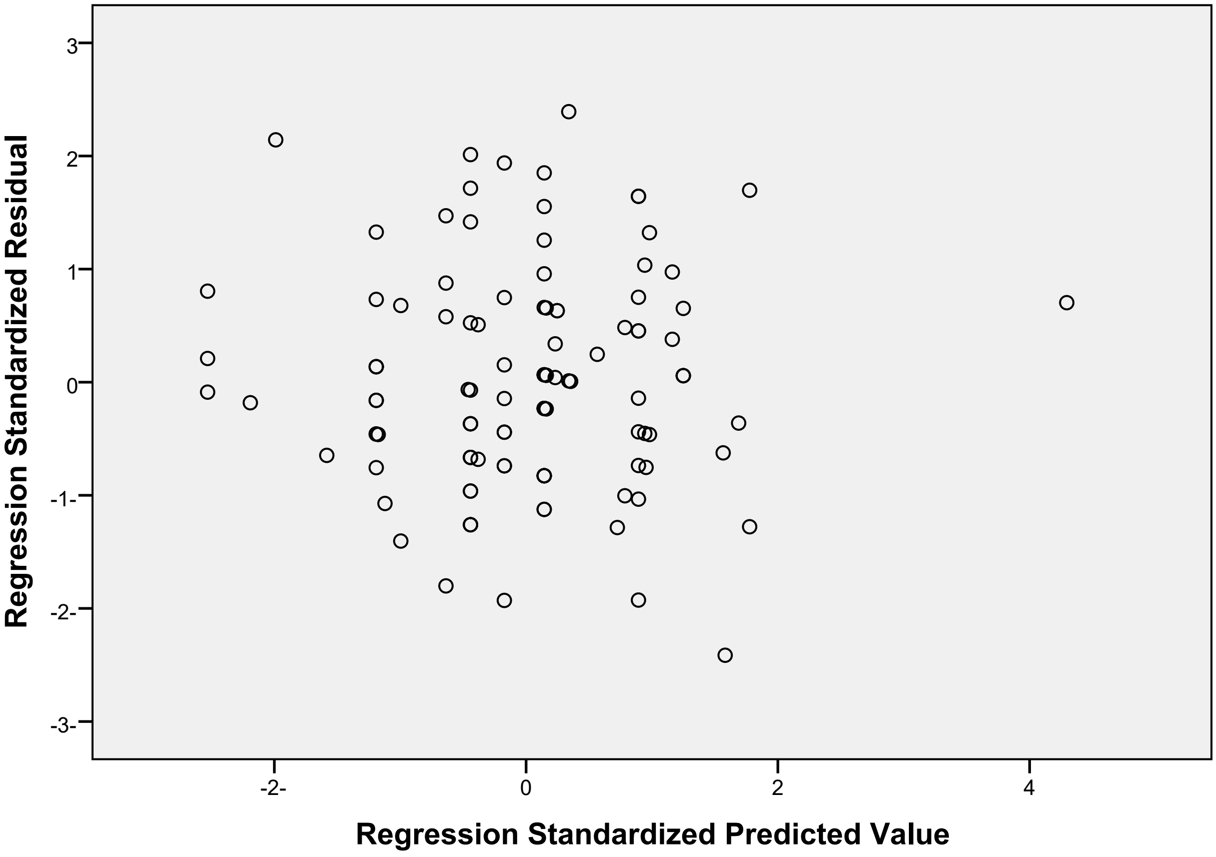

Supplement: Supplementary file 1 [file healthcare-14-03201-s001.zip › healthcare-4543679-supplementary.pdf]
